# 90-day oral toxicity study of a salmon nasal cartilage extract containing undenatured collagen and proteoglycan in Sprague-Dawley rats

**DOI:** 10.1371/journal.pone.0340675

**Published:** 2026-01-23

**Authors:** Kenji Takada, Hideharu Nakano

**Affiliations:** LINISE Co., Ltd., Sapporo, Hokkaido, Japan; Dr Anjali Chatterji Regional Research Institute for Homoeopathy, INDIA

## Abstract

Salmon nasal cartilage is a rich source of proteoglycan and collagen that is widely used in food products. In Japan, a novel extraction method has been developed and patented that enable the simultaneous production of proteoglycan and undenatured collagen from salmon nasal cartilage. This study evaluated the subchronic oral toxicity of this extract mixture (SCP Complex-LS, containing 40% proteoglycan and 40% undenatured collagen) in a 90-day repeated toxicity study in Sprague-Dawley rats, following by a 14-day recovery period. Rats (20/group; 10 males and 10 females) were administered SCP Complex-LS once daily by oral gavage at doses of 0 (vehicle), 10.3, 20.6, or 41.2 mg/kg body weight/day, with additional recovery groups (10/groups; 5 males and 5 females) receiving vehicle or the high dose. Clinical endpoints included mortality, clinical observations, body weight, food consumption, estrous cyclicity, ophthalmoscopy, clinical pathology, organ weights, gross pathology, and histopathology. No deaths or test item-related clinical signs were observed. Sporadic changes in hematological, biochemical, urinary, or organ weight parameters occurred in some dose groups but were small in magnitude, showed no consistent dose-response relationship, and were not corroborated by histopathological alterations. Histopathology revealed only minimal findings, such as mild inflammation or congestion in the liver, kidneys, and lungs, occurring at low incidence and with similar frequency in both control and high-dose groups. Estrous cycles remained within normal limits, and recovery groups showed no evidence of delayed or irreversible toxicity. Based on these findings, the No Observed Adverse Effect Level (NOAEL) for SCP Complex-LS was determined to be 41.2 mg/kg body weight/day in Sprague-Dawley rats under the conditions of this study, supporting its safety for use as a food ingredient within the expected range of human intake.

## Introduction

In Japan, salmon (*Oncorhynchus keta*) has traditionally been regarded as a fish of which “nothing is wasted”, and its various parts have been utilized in diverse ways since ancient times. [1,2] For over 200 years, salmon has been consumed in preparations such as salted grilled fillets, Izushi (fermented sushi), and simmered backbone, while the viscera are eaten as Mefun, the roe as Ikura, and the head region (Hizu) as Hizunamasu. In this way, each part of the salmon, including the flesh, entrails, roe, and Hizu, has been prepared according to its specific characteristics. Hizunamasu is prepared by slicing Hizu, sprinkling it with salt, draining the exuded moisture, and then marinating it in vinegar. This dish is traditionally eaten during the New Year period and as a side dish with alcoholic beverages. Anatomically, Hizu corresponds to the nasal cartilage of the salmon.

Salmon nasal cartilage contains proteoglycan (PG) and collagen. PG is a glycoprotein complex in which sulfated polysaccharides, known as glycosaminoglycans-such as chondroitin sulfate, dermatan sulfate, heparan sulfate, heparin, and keratan sulfate-are covalently bound to core proteins to form a macromolecular structure. Collagen is a protein widely distributed in animals, including mammals, birds, and fish, and serves as a major component of the extracellular matrix. In humans, collagen is estimated to account for approximately 25–30% of total body protein, and its molecular structure consists of three polypeptide chains, each with a molecular weight of approximately 100,000, that form a triple-helical structure stabilized by hydrogen bonds. The triple-helical structure of collagen is further stabilized by cross-links within the telopeptide regions located at the ends of the collagen molecules, thereby facilitating the formation of higher-order structures such as fibers and reticula. Undenatured collagen is defined as collagen in which this triple-helical structure remains intact, in contrast to gelatin or collagen peptides, in which the triple helix has been disrupted.

Conventional methods for extracting PG from salmon nasal cartilage involve the use of chaotropic agents such as guanidine hydrochloride (HN:C(NH_2_)_2_・HCl) or acidic solvents such as acetic acid (CH_3_COOH). Collagen can be extracted by heat-induced denaturation or by solubilization with acidic or alkaline solutions. However, alternative extraction methods that do not rely on such denaturing conditions have recently been developed. [3,4] SCP Complex-LS is a mixture containing approximately 40% PG and 40% undenatured collagen produced from salmon nasal cartilage using a newly patented extraction method in Japan. This material was supplied by LINISE Co., Ltd. The SCP Complex-LS used in the present study is the same preparation that was previously evaluated by Kuriyama et al. for its effects on knee joint pain in healthy volunteers. [5]

Several studies have evaluated the safety of cartilage-derived materials. Kudo et al. conducted a 90-day repeated toxicity study of salmon nasal cartilage powder containing approximately 40% PG in rats at a single dose of 1000 mg/kg/day and reported no treatment-related abnormalities. [6] However, that study did not assess dose-dependent effects across multiple dose levels. Similarly, Marone et al. evaluated the safety of undenatured type II collagen derived from chicken sternum cartilage in a 90-day subchronic toxicity study in rats and found no significant pathological changes at doses up to 400 mg/kg/day. [7] In contrast to these previous studies, which used single-dose or limited-dose designs and investigated either PG-rich powder or undenatured collagen alone, the present study focused on a salmon nasal cartilage extract containing both PG and undenatured collagen (SCP Complex-LS) and employed a dose-response design with four dose groups (0, 10.3, 20.6, and 41.2 mg/kg/day) over 90 days in male and female Sprague-Dawley rats. This design allowed a more detailed assessment of potential dose-dependent effects and enabled the establishment of No Observed Adverse Effect Level (NOAEL) for this specific extract composition.

Therefore, the objective of this study was to assess the subchronic oral safety of SCP Complex-LS, a mixture of PG and undenatured collagen produced by a recently developed extraction method from salmon nasal cartilage, and to evaluate its NOAEL in rats in order to verify the safety margin of the recommended intake levels for use in food products.

## Materials and methods

### Safety study

#### Compliance and guidelines.

This study was conducted in compliance with the OECD Principles of Good Laboratory Practice (GLP) (Revised 1997; issued January 1998; ENV/MC/CHEM(98)17). The study was conducted according to the mutually agreed study plan between LINISE Co., Ltd. and Accuprec Research Labs and accordance with the testing facility’s standard operating procedures (SOPs). The study was performed in accordance with OECD Guideline for the Testing of Chemicals No. 408: Repeated Dose 90- Day Oral Toxicity Study in Rodents (adopted 25 June 2018). The study was designed to use the minimum number of animals necessary to achieve the scientific objectives while considering applicable regulatory requirements. The protocol for animal use and general procedures was reviewed and approved by the Institutional Animal Ethics Committee (IAEC; approval no. ARL/PT/1198/2023). All animal procedures were performed in accordance with the recommendations of the Guide for the Care and Use of Laboratory Animals and the Committee for Control and Supervision of Experiments on Animals (CCSEA) guidelines, following ethical practices for animal care. All study personnel, including the Study Director, received comprehensive theoretical and hands-on training in animal handling, test item administration, and general animal care. Additional external training was provided as needed to support animal welfare and data integrity.

#### Animals and housing conditions.

Sprague-Dawley male and female rats (6–7 weeks old) were purchased from Rodent Research India Pvt. Ltd. (Haryana, India). Before randomization and the start of dosing, all animals were acclimatized to the laboratory conditions; male rats were acclimatized for 6 days and female rats for 7 days under the same environment conditions as during the study. During the acclimatization period, animals were monitored once daily for clinical signs and at least twice daily for mortality and morbidity to confirm their health status before treatment. No test substance was administered during this period. A total of 100 animals (50 males and 50 females) were selected from 110 animals and randomized by manual method based on body weight. Animals were allocated to different groups ensuring that the mean body weight variation across groups was minimal and did not exceed ±20% of the mean body weight for each sex. Rats were housed two to three per cage, and the animal rooms was maintained at a temperature of 20.0–22.9°C, a relative humidity of 40–65%, and 12 hour light/dark cycle. Animals were fed ad libitum with a standard pelleted laboratory animal diet manufactured by Nutrivet Life Sciences. Fresh potable drinking water processed through a reverse osmosis system was provided ad libitum to the animals in water bottles.

#### Study design and grouping.

**Fig 1:**
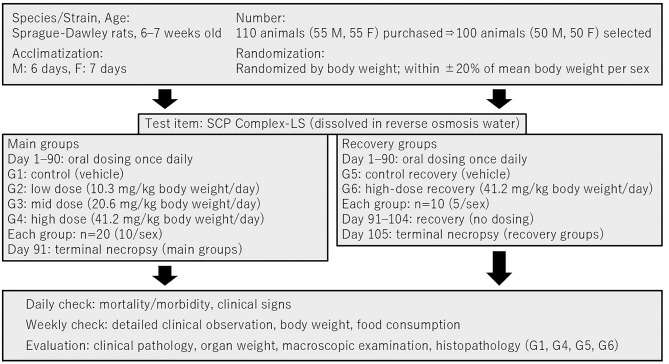
Schematic representation of the experimental design. Sprague-Dawley rats were randomized into six groups. Main groups (G1-G4; 10 males and 10 females per groups) received once-daily oral gavage of vehicle (G1), 10.3 mg/kg body weight/day (G2), 20.6 mg/kg body weight/day (G3), 41.2 mg/kg body weight/day (G4) for 90 consecutive days, followed by terminal necropsy on Day 91. Recovery groups (G5 and G6; 5 males and 5 females per groups) received vehicle (G5) or 41.2 mg/kg body weight/day (G6) for 90 days, followed by a 14-day recovery period without treatment and necropsy on Day 105. M, male; F, female.

The doses of the test substance were set to 0 (control group), 10.3 (low dose), 20.6 (mid dose), and 41.2 (high dose) mg/kg body weight (B.wt.)/day. Rats were divided into six groups: G1 (control group), G2 (low dose), G3 (mid dose), G4 (high dose), G5 (control recovery group), and G6 (high-dose recovery group). Each main group contained 20 rats (10 males, 10 females), and each recovery group contained 10 rats (5 males, 5 females). The dose levels were selected based on the anticipated human intake of SCP Complex-LS (100 mg/day, approximately 1.67 mg/kg/day for a 60 kg adult). Applying a safety factor of 6.2 to account for interspecies differences and individual variability, the high dose was set at 41.2 mg/kg B.wt./day in rats. The mid and low doses were established as one-half (20.6 mg/kg B.wt./day) and one-quarter (10.3 mg/kg B.wt./day) of the high dose, respectively. These dose levels were further supported by the absence of treatment-related adverse effects in a preceding substance toxicity study and were considered appropriate to evaluate potential subchronic toxicity in accordance with OECD Test Guideline 408. This study was conducted in accordance with OECD GLP principles and institutional SOPs. While full blinding was not feasible during dosing and clinical observations due to the practical requirements of dose preparation and administration, steps were taken to minimize potential bias (e.g., standardized procedures and predefined endpoints). Histopathological evaluations were performed by a veterinary pathologist at the contract testing facility, who was not blinded to the treatment groups. To support transparent interpretation, findings are reported organ-wise with incidence (x/total) and summarized in the corresponding tables. A schematic overview of the study design and grouping is shown in Fig 1.

#### Test substance preparation and administration.

SCP Complex-LS was dissolved in reverse osmosis (R.O.) water and administered orally by oral gavage using a 16-gauge gavage cannula attached to a syringe. The dosing volume was 10 mL/kg B.wt./day and was adjusted weekly based on the most recent body weight. Animals were handled and restrained appropriately during each administration. Dosing of male and female animals was initiated one day apart. The control group received R.O. water. Dosing was performed once daily for 90 consecutive days in the main groups. The recovery groups were dosed for 90 days, followed by a recovery period without dosing. The oral route was selected because it reflects the intended human route of test item administration and is consistent with OECD Test Guideline 408.

#### Animal welfare and humane endpoints.

Throughout the study period, beginning with the first administration of the test substance, animals were closely monitored for clinical signs suggestive of distress or declining health status, including prostration, impaired circulation, convulsive-like activity, and other severe abnormalities. Animals exhibiting such conditions were considered moribund and were humanely euthanized in accordance with pre-established humane endpoint criteria. Euthanasia was performed by overdose of isoflurane anesthesia followed by exsanguination, in accordance with institutional and internationally accepted ethical guidelines. Animals meeting humane endpoint criteria were euthanized within 18–24 hours of symptom onset to ensure timely and ethical intervention. No animals died spontaneously or required early termination beyond the pre-defined humane endpoint procedures. All scheduled terminal procedures were performed under anesthesia to minimize discomfort.

#### Study duration and observations.

The study was conducted from March 21, 2024 to September 18, 2024. During the study period, mortality/morbidity checks, clinical observations, detailed clinical observations, body weight, body weight gain, and food consumption were recorded. Mortality/morbidity and general clinical observations were performed daily, and mortality/morbidity was checked twice daily (morning and evening). Detailed clinical observations, body weight measurements, and food consumption measurements were performed on Day 1 and weekly thereafter. Body weight gain was calculated on a weekly basis from Day 1 using Equation 1. Food input (F.I.) and food left over (F.L.) were measured weekly throughout the study, and food consumption was calculated using Equation 2 and expressed as g/rat/day for each week. Ophthalmoscopy was performed prior to the start of dosing and again at scheduled termination. Vaginal smear collection was performed at scheduled termination. Histopathological examination was performed following necropsy at scheduled termination.

Equation 1. Equation for Body Weight Gain


$$\text{Body}\;\text{weight}\;\text{gain}(\%)=\text{100}\cdot \frac{\text{body}\;\text{weight}(\text{day}\;\text{Y})}{\text{body}\;\text{weight}(\text{day}\;\text{1})}-\text{100}$$

day Y: body weight recorded on weekly basis until terminal sacrifice (except fasted body weight) after treatment on day 1

day 1: body weight recorded on day 1 prior to treatment

The body weight gain data was expressed as Mean±SD

Equation 2. Equation for Food Consumption

$\text{Weekly}\;\text{food}\;\text{consumption}(\text{g}/\text{rat}/\text{day})=\text{Weekly}\;\text{F}.\text{I}.(\text{g})-\frac{\text{Weekly}\;\text{F}.\text{I}.(\text{g})}{\text{No}.\;\text{of}\;\text{days}\;\text{in}\;\text{a}\;\text{week}}\cdot \frac{\text{No}.\;\text{of}\;\text{animals}}{\text{cage}}$

#### Clinical pathology.

Clinical pathology assessments were performed for all surviving animals in G1-G6 using samples collected on Days 91 and 105 (main and recovery groups, respectively). Rats were fasted overnight in metabolic cages, with water provided ad libitum. Blood samples were collected from the retro-orbital plexus under isoflurane anesthesia. Approximately 2.0–2.5 mL of blood was obtained from each rat and used for hematology (Table 1), coagulation parameters (Table 2), and clinical chemistry (Table 3). Urine samples were collected from both sexes on Day 86 (G1 and G2), Day 87 (G3 and G4), and Day 104 (G5 and G6). Urinalysis was performed using a urine analyzer for the parameters listed in Table 4.

**Table 1:** Hematology.

Parameters	Units
Red Blood Cell count (RBC)	10^12^/L
White Blood Cell count (WBC)	10^9^/L
Reticulocyte (Retic)	%
Hemoglobin (HGB)	g/dL
Hematocrit (HCT)	%
Platelet Count (PLT)	10^3^/uL
Differential Leukocyte Count (DLC)	% & 10^9^/L
Neutrophils (Nec)	
Lymphocytes (Lymp)	
Basophils (Baso)	
Eosinophils (Eosi)	
Monocytes (Mono)	

**Table 2:** Coagulation Parameters.

Parameters	Unit
Prothrombin Time (PT)	Seconds
Activated Partial Thromboplastin Time (APTT)	Seconds

**Table 3:** Clinical Chemistry.

Parameters	Units
Glucose (GLU)	mg/dL
Total Protein (TP)	g/L
Triglycerides (TRG)	mg/dL
Total Cholesterol (CHO)	mg/dL
Low Density Lipoprotein (LDL)	mg/dL
High Density Lipoprotein (HDL)	mg/dL
Alanine Aminotransferase (ALT)	U/L
Aspartate Aminotransferase (AST)	U/L
Alkaline Phosphatase (ALP)	IU/L
Albumin (ALB)	mg/dL
Total Bilirubin (BIL)	mg/dL
Blood Urea Nitrogen (BUN)	mg/dL
Creatinine (CRE)	mg/dL
Calcium (Ca)	mg/dL
Sodium (Na)	mmol/L
Potassium (K)	mmol/L
Phosphorus (P)	mg/dL
T3	ng/mL
T4	ng/mL
TSH	uIU/mL

**Table 4:** Urine analysis.

Parameters	
Appearance	Glucose
Volume	Ketone Bodies
Color	Urobilinogen
Clarity	Bilirubin
Specific Gravity	Nitrites
pH	Blood
Protein	

#### Vaginal Cytology.

To determine the stage of the estrous cycle (proestrus, estrus, metestrus, and diestrus), vaginal smears were corrected from females in G1-G4 on Day 91 and from females in G5 and G6 on Day 105 prior to scheduled euthanasia. Smears were examined microscopically, and the types and percentages of cells were recorded.

#### Organ Weight and Histopathology.

Histopathological examination was performed on preserved organs and tissues from the control and high-dose group in both the main and recovery phases (G1, G4, G5, and G6). Because no severe or treatment-related lesions were identified in the high-dose group compared with controls, organs and tissues from the low- and mid-dose groups (G2 and G3) were not submitted for histopathological evaluation. Absolute organ weights were measured for the organs listed in Table 5, and relative organ weights were calculated using Equation 3. For histopathology, collected organs and tissues were processed by routine methods, sectioned, and stained with hematoxylin and eosin (H&E) for microscopic examination.

**Table 5:** Organ weighted and collected for histopathology.

Tissue	Weighted	Collected and Fixed
Brain	○	○
Thyroid	○	○
Adrenals	○	○
Heart	○	○
Aorta	×	○
Eye	×	○
Spleen	○	○
Esophagus	×	○
Thymus	○	○
Pituitary gland	○	○
Lung with bronchi and bronchioles	×	○
Stomach	×	○
Small intestine (Duodenum, Jejunum, Terminal Ileum)	×	○
Large intestine (Caecum, Colon, and Rectum)	×	○
Liver	○	○
Kidneys	○	○
Urinary Bladder	×	○
Ovaries (in female)	○	○
Uterus with cervix (in female)	○	○
Testes (in male)	○	○
Epididymis	○	○
Seminal vesicle	○	○
Prostate + Seminal Vesicles with coagulating glands	○	○
Lymph node (Mesenteric)	×	○
Skin along with Mammary glands	×	○

Equation 3. Equation for Relative Organ Weight


$$\text{Relative}\;\text{organ}\;\text{weight}(\%)=\frac{\text{absolute}\;\text{organ}\;\text{weight}}{\text{fasted}\;\text{body}\;\text{weight}}\cdot \text{100}$$

### Statistical analysis

The data were analyzed using GraphPad Prism software (version 8.4.3). Statistical analyses were performed for body weight, food consumption, clinical pathology parameters, and absolute and relative organ weights. Unless otherwise specified, a significant level of 5% was used; where appropriate, results at the 1% level are also reported. For each parameter, data were first assessed for homogeneity of variance. Homogeneous data were analyzed by one-way analysis of variance (ANOVA), followed by Dunnett’s multiple comparison test to compare each treated group with the corresponding control. Non-homogeneous data were transformed using an appropriate method prior to analysis.

## Result

### Mortality and clinical observation

No mortality or morbidity was observed in any group during the study period. Daily cage-side observations did not reveal any abnormal clinical signs. During the detailed weekly clinical examinations conducted before and during the dosing period, no abnormalities were observed in general appearance or behavior, including posture, gait, locomotor activity, and response to handling, and no clonic or tonic movements or stereotypic behaviors (e.g., excessive grooming or repetitive circling) were noted. No abnormalities were observed in the skin, fur, mucous membranes, eyes (e.g., lacrimation), ears, oral cavity, respiration, or excreta (urine/feces). Ophthalmoscopic examination of both eyes did not reveal any abnormalities in any group.

### Body Weight

**Fig 2:**
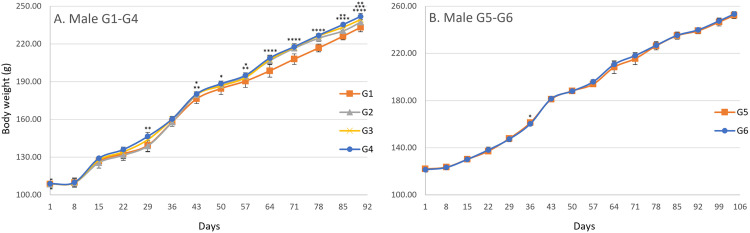
Body Weight changes in male rats during and after the 90-day dosing period. Body weight was measured weekly throughout the study. Data are presented as mean ± SD (n = 10 rats per group for main study groups; n = 5 rats per group for recovery groups). (A) Main study groups received SCP Complex-LS for 90 consecutive days: G1 (control, vehicle only), G2 (low dose, 10.3 mg/kg B.wt./day), G3 (mid dose, 20.6 mg/kg B.wt./day), and G4 (high dose, 41.2 mg/kg B.wt./day). (B) Recovery groups received treatment for 90 days followed by a 14-day recovery period: G5 (control recovery, vehicle only) and G6 (high-dose recovery, 41.2 mg/kg B.wt./day). Statistical comparisons at each time point were performed using one-way ANOVA followed by Dunnett’s multiple comparison test, comparing each treated group with the corresponding control (G1 vs G2-G4; G5 vs G6). Asterisks indicate statistically significant differences compared with the corresponding control group: *P < 0.05, **P < 0.01, ****P < 0.0001.

**Fig 3:**
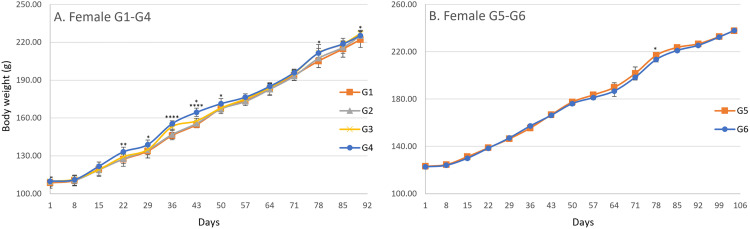
Body Weight changes in female rats during and after the 90-day dosing period. Body weight was measured weekly throughout the study. Data are presented as mean ± SD (n = 10 rats per group for main study groups; n = 5 rats per group for recovery groups). (A) Main study groups received SCP Complex-LS for 90 consecutive days: G1 (control, vehicle only), G2 (low dose, 10.3 mg/kg B.wt./day), G3 (mid dose, 20.6 mg/kg B.wt./day), and G4 (high dose, 41.2 mg/kg B.wt./day). (B) Recovery groups received treatment for 90 days followed by a 14-day recovery period: G5 (control recovery, vehicle only) and G6 (high-dose recovery, 41.2 mg/kg B.wt./day). Statistical comparisons at each time point were performed using one-way ANOVA followed by Dunnett’s multiple comparison test, comparing each treated group with the corresponding control (G1 vs G2-G4; G5 vs G6). Asterisks indicate statistically significant differences compared with the corresponding control group: *P < 0.05, **P < 0.01, ****P < 0.0001.

**Fig 4:**
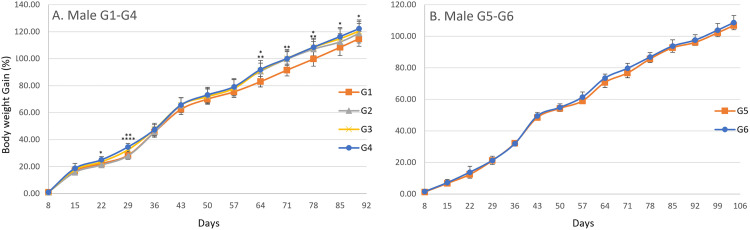
Body Weight Gain (%) in male rats during and after the 90-day dosing period. Body weight gain was calculated at each measurement point based on changes from the initial body weight. Data are presented as mean ± SD (n = 10 rats per group for main study groups; n = 5 rats per group for recovery groups). (A) Main study groups received SCP Complex-LS for 90 consecutive days: G1 (control, vehicle only), G2 (low dose, 10.3 mg/kg B.wt./day), G3 (mid dose, 20.6 mg/kg B.wt./day), and G4 (high dose, 41.2 mg/kg B.wt./day). (B) Recovery groups received treatment for 90 days followed by a 14-day recovery period: G5 (control recovery, vehicle only) and G6 (high-dose recovery, 41.2 mg/kg B.wt./day). No statistically significant differences in body weight gain were observed between G5 and G6. Statistical comparisons at each time point were performed using one-way ANOVA followed by Dunnett’s multiple comparison test, comparing each treated group with the corresponding control (G1 vs G2-G4; G5 vs G6). Asterisks indicate statistically significant differences compared with the corresponding control group: *P < 0.05, **P < 0.01, ****P < 0.0001.

**Fig 5:**
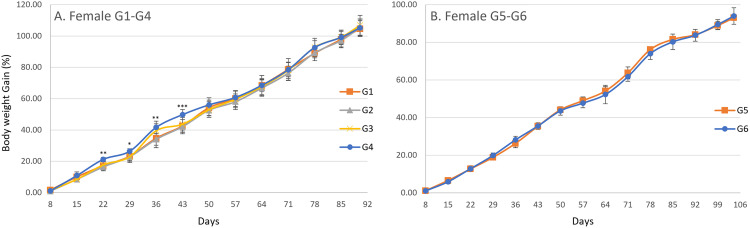
Body Weight Gain (%) in female rats during and after the 90-day dosing period. Body weight gain was calculated at each measurement point based on changes from the initial body weight. Data are presented as mean ± SD (n = 10 rats per group for main study groups; n = 5 rats per group for recovery groups). (A) Main study groups received SCP Complex-LS for 90 consecutive days: G1 (control, vehicle only), G2 (low dose, 10.3 mg/kg B.wt./day), G3 (mid dose, 20.6 mg/kg B.wt./day), and G4 (high dose, 41.2 mg/kg B.wt./day). (B) Recovery groups received treatment for 90 days followed by a 14-day recovery period: G5 (control recovery, vehicle only) and G6 (high-dose recovery, 41.2 mg/kg B.wt./day). No statistically significant differences in body weight gain were observed between G5 and G6. Statistical comparisons at each time point were performed using one-way ANOVA followed by Dunnett’s multiple comparison test, comparing each treated group with the corresponding control (G1 vs G2-G4; G5 vs G6). Asterisks indicate statistically significant differences compared with the corresponding control group: *P < 0.05, **P < 0.01, ****P < 0.0001.

**Fig 6:**
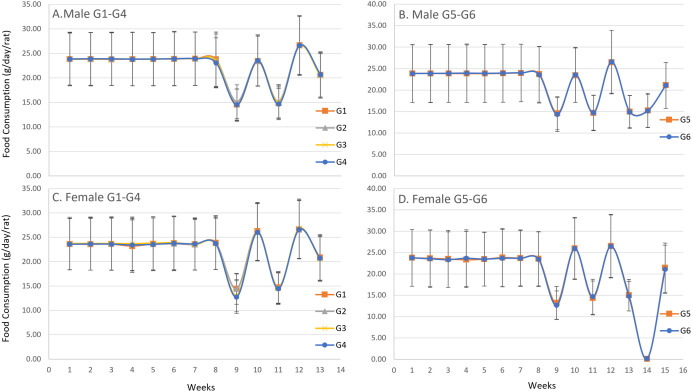
Food Consumption (g/day/rat) in male and female rats during and after the 90-day dosing period. Weekly food consumption was measured throughout the study. Data are presented as mean ± SD (n = 10 rats per group for main study groups; n = 5 rats per group for recovery groups). Main study groups received SCP Complex-LS for 90 consecutive days: G1 (control, vehicle only), G2 (low dose, 10.3 mg/kg B.wt./day), G3 (mid dose, 20.6 mg/kg B.wt./day), and G4 (high dose, 41.2 mg/kg B.wt./day). Recovery groups received treatment for 90 days followed by a 14-day recovery period: G5 (control recovery, vehicle only) and G6 (high-dose recovery, 41.2 mg/kg B.wt./day). (A) Male main study groups (G1-G4). (B) Male recovery groups (G5, G6). (C) Female main study groups (G1-G4). (D) Female recovery groups (G5, G6).

In the male main groups, significant increases in body weight were observed on day 29 in G4, day 43 in G2, G3, and G4, day 50 in G4, day 57 in G2 and G4, day 64 in G2, G3, and G4, day 71 in G2, G3, and G4, day 78 in G2, G3, and G4, and day 85 in G2, G3, and G4 compared with G1 (Fig 2A). In the male recovery groups, a significant decrease was observed on day 36 in G6 when compared with G5 (Fig 2B). In the female main groups, significant increases in body weight were observed on days 22 and 29 in G4, day 36 in G3 and G4, day 43 in G3 and G4, day 50 in G4, day 78 in G3 and G4, and day 90 in G3 compared with G1 (Fig 3A). In the female recovery groups, a significant decrease was observed on day 78 in G6 compared with G5 (Fig 3B). A gradual increase in body weight was observed from day 1 until termination in both males and females across all groups. For body weight gain, significant increases were observed in males on day 22 in G4, day 29 in G3 and G4, day 64 in G2, G3, and G4, day 71 in G2, G3, and G4, day 78 in G2, G3, and G4, day 85 in G4, and day 90 in G4 compared with G1 (Fig 4A), and in females on days 22, 29, 36, and 43 in G4 compared with G1 (Fig 5A). No significant differences were observed for body weight gain in either sex in the recovery groups (Fig 4B and 5B), nor for food consumption in either sex in the main or recovery groups (Fig 6). Although these sporadic differences in body weight and body weight gain reached statistical significance at some time points in the treated groups, they were small in magnitude and did not show any clear dose-related trend across the dose range tested.

Statistical comparisons at each time point were performed using one-way ANOVA followed by Dunnett’s multiple comparison test, comparing each treated group with the corresponding control (G1 vs G2-G4; G5 vs G6). No statistically significant differences in food consumption were observed in either sex in the main or recovery groups.

## Clinical pathology

### Hematology.

In the male main groups, significant decreases were observed for the relative lymphocytes in G4, reticulocyte in G2, G3 and G4 when compared with G1 and significant increase was observed in relative and absolute eosinophils in G4 when compared with G1. In the male recovery groups, significant decrease was observed for the relative neutrophile in G6 and significant increase was observed for the relative monocytes in G6 when compared with G5 (Table 6). In the female main groups, significant increase was observed for the relative lymphocytes in G3 and G4 when compared with G1 and significant decrease was observed for the relative basophile in G4, relative eosinophile in G3 and G4, absolute basophile in G4, absolute eosinophile in G2, G3 and G4, and reticulocyte in G4 when compared with G1. No significant changes were observed in recovery groups (Table 7).

**Table 6:** Hematology of male rats.

Male								
Parameter	Group							
	G1		G2		G3		G4	
	mean±SD	N	mean±SD	N	mean±SD	N	mean±SD	N
RBC (10^12^/L)	10.46 ± 0.58	10	10.30 ± 0.87	10	10.24 ± 0.58	10	10.43 ± 0.46	10
WBC (10^9^/L)	8.77 ± 3.32	10	8.59 ± 3.65	10	9.42 ± 3.60	10	9.94 ± 4.37	10
HGB (g/dL)	15.24 ± 0.99	10	15.28 ± 0.84	10	14.98 ± 1.19	10	15.44 ± 0.54	10
HCT (%)	50.12 ± 2.65	10	50.38 ± 3.74	10	50.92 ± 2.90	10	50.92 ± 1.96	10
PLT (10^3^/uL)	915.20 ± 199.95	10	959.60 ± 187.92	10	897.70 ± 160.56	10	965.40 ± 176.56	10
Neu^a^ (%)	13.72 ± 6.70	10	15.50 ± 5.21	10	12.90 ± 4.99	10	16.51 ± 6.86	10
Lymp^a^ (%)	66.42 ± 6.27	10	62.93 ± 5.04	10	62.23 ± 8.21	10	51.51 ± 16.12**	10
Eosi^a^ (%)	2.83 ± 2.15	10	5.78 ± 3.07	10	5.40 ± 3.55	10	15.66 ± 15.10**	10
Mono^a^ (%)	16.90 ± 4.56	10	15.49 ± 7.13	10	19.32 ± 5.90	10	16.08 ± 6.00	10
Baso^a^ (%)	0.14 ± 0.09	10	0.29 ± 0.19	10	0.16 ± 0.17	10	0.24 ± 0.23	10
Neu^b^ (10^9^/L)	1.04 ± 0.42	10	1.26 ± 0.58	10	1.15 ± 0.45	10	1.66 ± 0.99	10
Lymp^b^ (10^9^/L)	5.93 ± 2.66	10	5.50 ± 2.62	10	6.07 ± 3.12	10	5.04 ± 3.02	10
Eosi^b^ (10^9^/L)	0.24 ± 0.19	10	0.45 ± 0.24	10	0.45 ± 0.30	10	1.76 ± 2.08**	10
Mono^b^ (10^9^/L)	1.55 ± 0.92	10	1.35 ± 0.69	10	1.73 ± 0.58	10	1.46 ± 0.58	10
Baso^b^ (10^9^/L)	0.01 ± 0.01	10	0.03 ± 0.02	10	0.01 ± 0.02	10	0.02 ± 0.03	10
Retic (%)	15.06 ± 1.41	10	8.44 ± 4.90****	10	7.66 ± 0.78****	10	7.01 ± 0.77****	10
**Parameter**	**Group**							
	**G5**					**G6**		
	**mean±SD**		**N**			**mean±SD**		**N**
RBC (10^12^/L)	10.59 ± 0.42		5			10.98 ± 0.35		5
WBC (10^9^/L)	5.23 ± 0.48		5			7.75 ± 3.33		5
HGB (g/dL)	16.52 ± 0.57		5			16.24 ± 0.99		5
HCT (%)	53.76 ± 1.46		5			53.38 ± 3.63		5
PLT (10^3^/uL)	882.00 ± 243.83		5			870.80 ± 115.69		5
Neu^a^ (%)	23.90 ± 6.46		5			14.22 ± 4.28*		5
Lymp^a^ (%)	56.08 ± 5.76		5			60.63 ± 4.63		5
Eosi^a^ (%)	5.99 ± 1.17		5			6.54 ± 1.49		5
Mono^a^ (%)	13.88 ± 1.69		5			18.43 ± 3.22*		5
Baso^a^ (%)	0.15 ± 0.08		5			0.19 ± 0.14		5
Neu^b^ (10^9^/L)	1.26 ± 0.39		5			0.99 ± 0.23		5
Lymp^b^ (10^9^/L)	2.9 ± 0.34		5			4.79 ± 2.18		5
Eosi^b^ (10^9^/L)	0.31 ± 0.07		5			0.51 ± 0.25		5
Mono^b^ (10^9^/L)	0.73 ± 0.12		5			1.44 ± 0.77		5
Baso^b^ (10^9^/L)	0.01 ± 0.00		5			0.01 ± 0.01		5
Retic (%)	9.26 ± 2.30		5			7.46 ± 0.59		5

^a^Represents Relative Leucocytes Counts, ^b^Represents Absolute Leucocytes Counts

Neu^a^: *P < 0.05(0.0261) in G6 with significant decrease at 95% confidence interval; Lymp^a^: **P < 0.05(0.0051) in G4 with significant decrease at 95% confidence interval; Eosi^a^: **P < 0.05(0.0027) in G4 with significant increase at 95% confidence interval; Mono^a^: *P < 0.05(0.0360) in G6 with significant increase at 95% confidence interval; Eosi^b^: **P < 0.05(0.0080) in G4 with significant increase at 95% confidence interval; Retic: ****P < 0.0001 in G2,G3 & G4 with significant decrease at 95% confidence interval. (G2, G3, and G4 are compared with G1/ G6 is compared with G5)

**Table 7:** Hematology of female rats.

Female								
Parameter	Group							
	G1		G2		G3		G4	
	mean±SD	N	mean±SD	N	mean±SD	N	mean±SD	N
RBC (10^12^/L)	8.89 ± 0.88	10	9.01 ± 1.40	10	8.90 ± 1.21	10	9.13 ± 1.14	10
WBC (10^9^/L)	6.59 ± 2.16	10	6.53 ± 2.49	10	7.21 ± 3.81	10	6.66 ± 3.83	10
HGB (g/dL)	13.94 ± 1.35	10	14.28 ± 2.08	10	14.30 ± 1.68	10	14.54 ± 1.41	10
HCT (%)	44.67 ± 5.39	10	45.22 ± 7.61	10	45.17 ± 5.83	10	46.01 ± 5.61	10
PLT (10^3^/uL)	904.80 ± 369.54	10	985.60 ± 321.95	10	1110.80 ± 304.12	10	654.20 ± 247.43	10
Neu^a^ (%)	15.01 ± 4.93	10	18.57 ± 6.67	10	16.64 ± 6.34	10	14.89 ± 8.48	10
Lymp^a^ (%)	49.64 ± 7.01	10	50.73 ± 11.26	10	60.58 ± 7.88*	10	60.06 ± 7.39*	10
Eosi^a^ (%)	19.46 ± 8.25	10	13.91 ± 4.04	10	7.88 ± 4.99***	10	7.76 ± 7.16***	10
Mono^a^ (%)	15.59 ± 5.35	10	16.46 ± 11.24	10	14.67 ± 3.38	10	17.15 ± 5.22	10
Baso^a^ (%)	0.31 ± 0.08	10	0.33 ± 0.16	10	0.24 ± 0.18	10	0.14 ± 0.12*	10
Neu^b^ (10^9^/L)	0.93 ± 0.21	10	1.21 ± 0.76	10	1.04 ± 0.53	10	1.07 ± 0.99	10
Lymp^b^ (10^9^/L)	3.34 ± 1.45	10	3.34 ± 1.72	10	4.59 ± 2.79	10	4.10 ± 2.52	10
Eosi^b^ (10^9^/L)	1.28 ± 0.70	10	0.84 ± 0.21*	10	0.43 ± 0.25***	10	0.37 ± 0.22****	10
Mono^b^ (10^9^/L)	1.02 ± 0.42	10	1.12 ± 0.95	10	1.13 ± 0.70	10	1.12 ± 0.61	10
Baso^b^ (10^9^/L)	0.02 ± 0.01	10	0.02 ± 0.01	10	0.01 ± 0.01	10	0.01 ± 0.01*	10
Retic (%)	6.30 ± 0.96	10	6.17 ± 0.54	10	5.67 ± 0.28	10	5.63 ± 0.42*	10
Parameter	Group							
	G5				G6			
	mean±SD		N		mean±SD			N
RBC (10^12^/L)	9.51 ± 1.15		5		8.79 ± 1.84			5
WBC (10^9^/L)	5.53 ± 2.63		5		5.32 ± 3.54			5
HGB (g/dL)	15.04 ± 1.74		5		14.40 ± 2.94			5
HCT (%)	48.48 ± 5.59		5		45.68 ± 9.73			5
PLT (10^3^/uL)	974.00 ± 191.77		5		875.20 ± 195.52			5
Neu^a^ (%)	18.47 ± 2.98		5		20.30 ± 7.02			5
Lymp^a^ (%)	58.25 ± 5.29		5		57.65 ± 7.81			5
Eosi^a^ (%)	8.09 ± 6.00		5		4.88 ± 3.11			5
Mono^a^ (%)	14.96 ± 3.66		5		17.01 ± 2.52			5
Baso^a^ (%)	0.23 ± 0.09		5		0.17 ± 0.14			5
Neu^b^ (10^9^/L)	1.01 ± 0.46		5		0.98 ± 0.75			5
Lymp^b^ (10^9^/L)	3.29 ± 1.75		5		3.26 ± 2.32			5
Eosi^b^ (10^9^/L)	0.34 ± 0.26		5		0.18 ± 0.11			5
Mono^b^ (10^9^/L)	0.89 ± 0.59		5		0.88 ± 0.52			5
Baso^b^ (10^9^/L)	0.01 ± 0.01		5		0.01 ± 0.01			5
Retic (%)	7.50 ± 0.30		5		6.92 ± 0.33			5

^a^Represents Relative Leucocytes Counts, ^b^Represents Absolute Leucocytes Counts

Lympa: *P < 0.05(0.0190 & 0.265) in G3 & G4 with significant increase at 95% confidence interval Basoa: *P < 0.05(0.0320) in G4 with significant decrease at 95% confidence interval; Eosia: ***P < 0.05(0.0007 & 0.0006) in G3 & G4 with significant decrease at 95% confidence interval; Basoa: *P < 0.05(0.0252) in G4 with significant decrease at 95% confidence interval; Eosib: * & ***P < 0.05(0.0252 & 0.0001) in G2 & G3 & ****P < 0.0001 in G4 with significant decrease at 95% confidence interval; Retic: *P < 0.05(0.0484) in G4 with significant decrease at 95% confidence interval. (G2, G3, and G4 are compared with G1)

### Coagulation parameters.

In male, significant increase was observed for the prothrombin time in G2 and G3 compared with G1 in main group animals. In recovery group animals, significant increase was observed for the prothrombin time in G6 compared with G5 (Table 8). In female, significant increase was observed for the prothrombin time in G3 when compared with G1, and significant decrease was observed for the activated partial thromboplastin time in G2 and G4 compared with G1 (Table 9).

**Table 8:** Coagulation parameters of male rats.

Male								
Parameter	Group							
	G1		G2		G3		G4	
	mean±SD	N	mean±SD	N	mean±SD	N	mean±SD	N
PT (Sec)	14.06 ± 0.99	10	15.58 ± 0.77**	10	15.87 ± 1.33***	10	14.42 ± 0.90	10
APTT (Sec)	29.68 ± 1.71	10	30.20 ± 0.94	10	28.83 ± 2.96	10	28.75 ± 3.32	10
**Parameter**	**Group**							
	**G5**				**G6**			
	**mean±SD**		**N**		**mean±SD**			**N**
PT (Sec)	12.04 ± 0.47		5		13..50 ± 0.48*			5
APTT (Sec)	29.74 ± 1.66		5		31.98 ± 1.50			5

PT: ** & ***P < 0.05(0.0054 & 0.0009) in G2 & G3 with significant increase at 95% confidence interval; PT: *P < 0.05(0.0160) in G6 with significant increase at 95% confidence interval. (G2, G3, and G4 are compared with G1/ G6 is compared with G5)

**Table 9:** Coagulation parameters of female rats.

Female										
Parameter	Group									
	G1		G2		G3				G4	
	mean±SD	N	mean±SD	N	mean±SD			N	mean±SD	N
PT (Sec)	13.15 ± 0.91	10	14.60 ± 0.43	10	14.78 ± 2.39*		10		14.56 ± 0.82	10
APTT (Sec)	31.31 ± 0.53	10	30.11 ± 0.33*	10	21.50 ± 1.57		10		29.12 ± 1.05****	10
**Parameter**	**Group**									
	**G5**					**G6**				
	**mean±SD**		**N**			**mean±SD**				**N**
PT (Sec)	13.76 ± 0.59		5			14.52 ± 1.39				5
APTT (Sec)	30.60 ± 1.12		5			29.84 ± 0.96				5

PT: *P < 0.05(0.0295) in G3 with significant increase at 95% confidence interval; APTT: *P < 0.05(0.0282) in G2 & ****P < 0.0001 in G4 with significant decrease at 95% confidence interval. (G2, G3, and G4 are compared with G1)

### Urine analysis.

Approximately 5–10 mL of urine was collected from each male and female rat after overnight fasting. As shown in Table 10, 11 urinalysis parameters were evaluated: color, clarity, blood, bilirubin, urobilinogen, ketone bodies, glucose, protein, pH, nitrite, and specific gravity. All urinalysis results were within expected physiological ranges and showed no treatment-related findings. Therefore, no test item-related effects were identified in urinalysis in either the main or recovery groups.

**Table 10:** Urine analysis.

Male					
Parameter	Observation	No. of animals showing observation of particular parameter per total number of animals examined per group			
		Group			
		G1	G2	G3	G4
Color	Yellow	3/10	2/10	5/10	3/10
	Dark Yellow	3/10	3/10	3/10	4/10
	Straw	3/10	5/10	0/10	0/10
	Pale Yellow	1/10	0/10	2/10	3/10
Clarity	Turbid	5/10	4/10	6/10	1/10
	Cloudy	3/10	2/10	2/10	3/10
	Clear	2/10	4/10	2/10	4/10
	Dark	0/10	0/10	0/10	2/10
Blood (Ery/uL)	Negative	8/10	8/10	9/10	10/10
	10	1/10	1/10	1/10	0/10
	50	1/10	1/10	0/10	0/10
Bilirubin (mg/dL)	Negative	9/10	8/10	7/10	9/10
	3	1/10	1/10	0/10	0/10
	1	0/10	1/10	3/10	1/10
Urobilinogen (mg/dL)	Normal	6/10	7/10	9/10	10/10
	1	4/10	1/10	1/10	0/10
	3	0/10	2/10	0/10	0/10
Ketone Bodies (mg/dL)	Negative	4/10	3/10	6/10	9/10
	Trace	5/10	6/10	4/10	1/10
	16	1/10	1/10	0/10	0/10
Glucose	Negative	10/10	10/10	10/10	10/10
Protein (mg/dL)	100	5/10	4/10	4/10	5/10
	500	4/10	5/10	2/10	1/10
	Negative	1/10	0/10	1/10	3/10
	30	0/10	1/10	3/10	1/10
pH	9	7/10	9/10	9/10	10/10
	8	2/10	0/10	1/10	0/10
	7.5	0/10	1/10	0/10	0/10
	7	1/10	0/10	0/10	0/10
Nitrites	Negative	7/10	5/10	1/10	4/10
	Positive	3/10	5/10	9/10	6/10
Specific Gravity	1.000	9/10	9/10	10/10	10/10
	1.005	0/10	1/10	0/10	0/10
	1.025	1/10	0/10	0/10	0/10
Parameter	Observation	No. of animals showing observation of particular parameter per total number of animals examined per group			
		Group			
		G5		G6	
Color	Yellow	3/5		2/5	
	Dark Yellow	0/5		1/5	
	Straw	0/5		1/5	
	Pale Yellow	2/5		1/5	
Clarity	Turbid	1/5		3/5	
	Cloudy	4/5		0/5	
	Clear	0/5		2/5	
Blood	Negative	5/5		5/5	
Bilirubin (mg/dL)	Negative	4/5		5/5	
	1	1/5		0/5	
Urobilinogen	Normal	5/5		5/5	
Ketone Bodies	Negative	2/5		3/5	
	Trace	3/5		2/5	
Glucose	Negative	5/5		5/5	
Protein (mg/dL)	100	2/5		1/5	
	500	1/5		4/5	
	30	2/5		0/5	
pH	9	5/5		5/5	
Nitrites	Negative	0/5		1/5	
	Positive	5/5		4/5	
Specific Gravity	1.000	5/5		5/5	
Female					
Parameter	Observation	No. of animals showing observation of particular parameter per total number of animals examined per group			
		Group			
		G1	G2	G3	G4
Color	Yellow	3/10	5/10	4/10	5/10
	Dark Yellow	4/10	3/10	2/10	0/10
	Straw	1/10	0/10	1/10	2/10
	Pale Yellow	2/10	2/10	3/10	3/10
Clarity	Turbid	4/10	6/10	3/10	1/10
	Cloudy	5/10	2/10	4/10	5/10
	Clear	1/10	2/10	3/10	4/10
	Dark	0/10	0/10	0/10	0/10
Blood (Ery/uL)	Negative	9/10	8/10	8/10	0/10
	10	1/10	2/10	2/10	10/10
	50	0/10	0/10	0/10	0/10
Bilirubin (mg/dL)	Negative	10/10	6/10	7/10	1/10
	3	0/10	0/10	2/10	8/10
	1	0/10	3/10	1/10	0/10
	6	0/10	1/10	0/10	1/10
Urobilinogen (mg/dL)	Normal	10/10	6/10	7/10	0/10
	1	0/10	4/10	1/10	0/10
	3	0/10	0/10	0/10	0/10
	6	0/10	0/10	1/10	1/10
	12	0/10	0/10	1/10	9/10
Ketone Bodies (mg/dL)	Negative	10/10	7/10	7/10	1/10
	Trace	0/10	3/10	2/10	1/10
	16	0/10	0/10	1/10	8/10
Glucose	Negative	10/10	10/10	10/10	10/10
Protein (mg/dL)	100	5/10	5/10	8/10	1/10
	500	4/10	4/10	1/10	0/10
	Negative	0/10	0/10	1/10	9/10
	30	1/10	1/10	0/10	0/10
pH	9	10/10	9/10	6/10	0/10
	8	0/10	0/10	2/10	0/10
	7.5	0/10	0/10	0/10	0/10
	7	0/10	1/10	0/10	0/10
	6	0/10	0/10	2/10	10/10
Nitrites	Negative	4/10	4/10	8/10	0/10
	Positive	6/10	6/10	2/10	10/10
Specific Gravity	1.000	10/10	10/10	8/10	0/10
	1.005	0/10	0/10	0/10	0/10
	1.030	0/10	0/10	2/10	10/10
Parameter	Observation	No. of animals showing observation of particular parameter per total number of animals examined per group			
		Group			
		G5		G6	
Color	Yellow	2/5		3/5	
	Dark Yellow	2/5		0/5	
	Straw	0/5		1/5	
	Pale Yellow	1/5		1/5	
Clarity	Turbid	2/5		2/5	
	Cloudy	2/5		1/5	
	Clear	1/5		2/5	
Blood (Ery/uL)	Negative	4/5		3/5	
	10	1/5		2/5	
Bilirubin (mg/dL)	Negative	4/5		5/5	
	3	1/5		0/5	
Urobilinogen (mg/dL)	Normal	2/5		5/5	
	1	3/5		0/5	
Ketone Bodies	Negative	2/5		5/5	
	Trace	3/5		0/5	
Glucose	Negative	5/5		5/5	
Protein (mg/dL)	100	2/5		4/5	
	500	3/5		1/5	
pH	9	5/5		5/5	
Nitrites	Negative	3/5		5/5	
	Positive	2/5		0/5	
Specific Gravity	1.000	5/5		5/5	

**Table 11:** Clinical chemistry of male rats.

Male								
Parameter	Group							
	G1		G2		G3		G4	
	mean±SD	N	mean±SD	N	mean±SD	N	mean±SD	N
Glu (mg/dL)	110.60 ± 14.16	10	126.97 ± 16.95	10	135.50 ± 16.30*	10	129.46 ± 31.82	10
TP (g/L)	6.26 ± 0.31	10	6.13 ± 0.18	10	6.03 ± 0.23	10	6.11 ± 0.30	10
ALT (U/L)	57.41 ± 14.41	10	49.32 ± 6.44	10	44.21 ± 3.07**	10	45.35 ± 4.88**	10
AST (U/L)	78.79 ± 7.94	10	79.35 ± 8.92	10	76.62 ± 9.23	10	76.00 ± 10.31	10
ALP (IU/L)	79.73 ± 8.82	10	80.28 ± 8.96	10	83.47 ± 4.82	10	75.94 ± 10.01	10
BIL (mg/dL)	0.08 ± 0.01	10	0.08 ± 0.01	10	0.08 ± 0.01	10	0.08 ± 0.01	10
BUN (mg/dL)	10.59 ± 0.59	10	10.29 ± 0.47	10	10.19 ± 0.52	10	10.42 ± 0.80	10
CRE (mg/dL)	1.02 ± 0.18	10	0.91 ± 0.08	10	1.04 ± 0.32	10	0.86 ± 0.18	10
TRG (mg/dL)	71.70 ± 25.56	10	64.68 ± 16.16	10	63.34 ± 28.57	10	63.04 ± 13.68	10
CHO (mg/dL)	57.78 ± 13.26	10	56.97 ± 12.04	10	53.82 ± 12.46	10	51.16 ± 10.53	10
Na (mmol/L)	138.36 ± 0.65	10	138.67 ± 0.64	10	139.02 ± 0.94	10	138.88 ± 0.73	10
K (mmol/L)	4.86 ± 0.47	10	5.04 ± 0.34	10	4.70 ± 0.24	10	4.89 ± 0.59	10
P (mg/dL)	6.58 ± 0.47	10	6.52 ± 0.34	10	6.63 ± 0.36	10	7.12 ± 0.62*	10
ALB (mg/dL)	3.23 ± 0.19	10	3.25 ± 0.10	10	3.48 ± 0.13**	10	3.52 ± 0.16***	10
Ca (mg/dL)	11.18 ± 0.58	10	11.15 ± 0.48	10	10.54 ± 0.40*	10	11.38 ± 0.71	10
HDL (mg/dL)	41.02 ± 1.00	10	40.96 ± 1.45	10	41.43 ± 0.39	10	41.43 ± 0.63	10
LDL (mg/dL)	31.15 ± 0.64	10	30.68 ± 0.47	10	30.76 ± 1.14	10	31.19 ± 0.95	10
T3 (ng/ml)	6.05 ± 0.73	10	6.14 ± 0.82	10	6.54 ± 0.47	10	6.55 ± 0.69	10
T4 (ng/ml)	76.51 ± 28.65	10	63.52 ± 23.66	10	58.77 ± 17.25	10	62.19 ± 25.42	10
TSH (ulU/ml)	1.76 ± 0.78	10	1.82 ± 0.58	10	2.18 ± 0.20	10	2.23 ± 0.37	10
Parameter	Group							
	G5				G6			
	mean±SD		N		mean±SD			N
Glu (mg/dL)	96.64 ± 3.54		5		96.08 ± 2.42			5
TP (g/L)	6.81 ± 1.03		5		7.94 ± 0.39			5
ALT (U/L)	32.48 ± 2.66		5		40.50 ± 4.02*			5
AST (U/L)	49.68 ± 7.10		5		50.36 ± 3.27			5
ALP (IU/L)	101.04 ± 4.48		5		101.30 ± 7.33			5
BIL (mg/dL)	0.29 ± 0.06		5		0.30 ± 0.04			5
BUN (mg/dL)	10.31 ± 0.65		5		12.86 ± 5.97			5
CRE (mg/dL)	0.68 ± 0.13		5		0.57 ± 0.07			5
TRG (mg/dL)	50.08 ± 6.57		5		63.88 ± 21.21			5
CHO (mg/dL)	62.28 ± 5.85		5		67.86 ± 1.53			5
Na (mmol/L)	139.92 ± 0.85		5		139.80 ± 1.52			5
K (mmol/L)	4.84 ± 0.23		5		5.65 ± 0.77			5
P (mg/dL)	6.44 ± 0.37		5		5.96 ± 0.39			5
ALB (mg/dL)	4.03 ± 0.10		5		4.09 ± 0.10			5
Ca (mg/dL)	10.86 ± 0.81		5		10.62 ± 1.30			5
HDL (mg/dL)	41.48 ± 5.50		5		38.68 ± 2.64			5
LDL (mg/dL)	33.58 ± 5.47		5		31.42 ± 4.05			5
T3 (ng/ml)	7.60 ± 0.69		5		7.20 ± 0.37			5
T4 (ng/ml)	100.09 ± 6.23		5		100.09 ± 6.59			5
TSH (ulU/ml)	1.40 ± 0.55		5		2.00 ± 0.00			5

Glu: *P < 0.05(0.0316) in G3 with significant increase at 95% confidence interval; ALT: **P < 0.05(0.0034 & 0.0078) in G3 & G4 with significant decrease & *P < 0.05(0.0310) in G6 with significant increase at 95% confidence interval; P: *P < 0.05(0.0352) in G4 with significant increase at 95% confidence interval; ALB: ** & ***P < 0.05(0.0012 & 0.0002) in G3 & G4 with significant increase at 95% confidence interval; Ca: *P < 0.05(0.0375) in G3 with significant decrease at 95% confidence interval. (G2, G3, and G4 are compared with G1)

### Clinical chemistry.

In clinical chemistry, in main group males, significant decrease was observed for the ALT in G3 and G4 and calcium in G3 compared with G1 and significant increase was observed for the glucose in G3, phosphorus in G4 and albumin in G3 and G4 compared with G1. In recovery group, significant increase for the ALT in G6 was observed compared with G5 (Table 11). In main group female, significant decrease was observed for the creatine in G3 and G4, total protein in G3 and G4 and albumin in G3 and G4, also significant increase was observed for the calcium in G4 and cholesterol in G3 and G4 compared with G1. In recovery group females, significant increase was observed for the LDL in G6 when compared with G5 (Table 12).

**Table 12:** Clinical chemistry of female rats.

Female								
Parameter	Group							
	G1		G2		G3		G4	
	mean±SD	N	mean±SD	N	mean±SD	N	mean±SD	N
Glu (mg/dL)	95.97 ± 14.63	10	103.76 ± 17.58	10	100.07 ± 16.53	10	101.88 ± 14.03	10
TP (g/L)	6.83 ± 0.27	10	6.71 ± 0.54	10	6.09 ± 0.52**	10	6.18 ± 0.43**	10
ALT (U/L)	45.30 ± 6.22	10	50.57 ± 10.18	10	44.14 ± 1.94	10	45.19 ± 1.77	10
AST (U/L)	75.65 ± 13.00	10	77.13 ± 8.98	10	75.52 ± 13.22	10	82.24 ± 10.82	10
ALP (IU/L)	79.32 ± 7.12	10	83.38 ± 10.68	10	79.64 ± 14.86	10	78.82 ± 11.86	10
BIL (mg/dL)	0.08 ± 0.01	10	0.07 ± 0.01	10	0.08 ± 0.01	10	0.08 ± 0.01	10
BUN (mg/dL)	10.93 ± 0.85	10	10.72 ± 0.75	10	11.58 ± 3.23	10	11.02 ± 0.63	10
CRE (mg/dL)	1.14 ± 0.12	10	1.16 ± 0.13	10	0.83 ± 0.06****	10	0.76 ± 0.04****	10
TRG (mg/dL)	72.44 ± 10.25	10	65.44 ± 16.85	10	74.59 ± 9.88	10	72.99 ± 23.42	10
CHO (mg/dL)	61.03 ± 14.91	10	61.82 ± 15.65	10	80.63 ± 9.12**	10	80.53 ± 8.87**	10
Na (mmol/L)	138.43 ± 0.62	10	138.83 ± 1.01	10	138.55 ± 0.73	10	138.80 ± 0.83	10
K (mmol/L)	4.62 ± 0.33	10	4.39 ± 0.30	10	4.63 ± 0.31	10	4.87 ± 0.51	10
P (mg/dL)	6.10 ± 0.40	10	6.00 ± 0.39	10	6.09 ± 0.43	10	6.03 ± 0.37	10
ALB (mg/dL)	3.64 ± 0.09	10	3.65 ± 0.11	10	3.31 ± 0.13****	10	3.43 ± 0.18**	10
Ca (mg/dL)	11.30 ± 0.50	10	11.23 ± 0.67	10	11.51 ± 0.56	10	11.99 ± 0.69*	10
HDL (mg/dL)	40.28 ± 3.15	10	41.28 ± 0.61	10	41.44 ± 0.73	10	40.10 ± 3.11	10
LDL (mg/dL)	30.74 ± 1.44	10	29.71 ± 1.16	10	30.48 ± 1.30	10	29.50 ± 0.85	10
T3 (ng/ml)	8.23 ± 1.63	10	7.77 ± 1.29	10	7.23 ± 1.19	10	7.03 ± 1.32	10
T4 (ng/ml)	81.32 ± 12.20	10	79.19 ± 13.14	10	71.57 ± 12.19	10	71.57 ± 16.37	10
TSH (ulU/ml)	1.52 ± 0.53	10	1.88 ± 0.29	10	1.72 ± 0.22	10	1.70 ± 0.19	10
Parameter	Group							
	G5				G6			
	mean±SD		N		mean±SD			N
Glu (mg/dL)	96.96 ± 2.27		5		94.72 ± 3.61			5
TP (g/L)	6.83 ± 0.61		5		6.74 ± 0.31			5
ALT (U/L)	43.64 ± 4.41		5		49.14 ± 8.36			5
AST (U/L)	53.18 ± 4.33		5		57.36 ± 9.22			5
ALP (IU/L)	95.52 ± 4.59		5		98.80 ± 2.34			5
BIL (mg/dL)	0.32 ± 0.06		5		0.34 ± 0.07			5
BUN (mg/dL)	0.32 ± 0.06		5		0.34 ± 0.07			5
CRE (mg/dL)	0.62 ± 0.06		5		0.65 ± 0.10			5
TRG (mg/dL)	48.80 ± 5.02		5		46.40 ± 6.74			5
CHO (mg/dL)	55.28 ± 3.59		5		57.24 ± 6.89			5
Na (mmol/L)	138.04 ± 1.02		5		138.50 ± 0.58			5
K (mmol/L)	4.73 ± 0.31		5		4.65 ± 0.21			5
P (mg/dL)	5.64 ± 0.42		5		5.34 ± 0.40			5
ALB (mg/dL)	4.71 ± 0.28		5		4.90 ± 0.49			5
Ca (mg/dL)	10.87 ± 1.19		5		11.71 ± 0.95			5
HDL (mg/dL)	37.86 ± 11.00		5		47.48 ± 3.01			5
LDL (mg/dL)	25.24 ± 5.63		5		31.36 ± 5.07*			5
T3 (ng/ml)	9.62 ± 0.71		5		9.12 ± 0.39			5
T4 (ng/ml)	88.14 ± 5.51		5		91.78 ± 5.91			5
TSH (ulU/ml)	1.47 ± 0.46		5		1.96 ± 0.20			5

CRE: ****P < 0.0001 in G3 & G4 with significant decrease at 95% confidence interval; TP: **P < 0.05(0.0025 & 0.0084) in G3 & G4 with significant decrease at 95% confidence interval; CHO: **P < 0.05(0.0036 & 0.0038) in G4 with significant increase at 95% confidence interval; ALB: ****P < 0.0001 & **P < 0.05(0.0035) in G3 & G4 respectively with significant decrease at 95% confidence interval; Ca: *P < 0.05(0.0421) in G4 with significant increase at 95% confidence interval; LDL: *P < 0.05(0.0131) in G6 with significant increase at 95% confidence interval. (G2, G3, and G4 are compared with G1/ G6 is compared with G5)

### Vaginal cytology

Vaginal cytology showed that female rats were in proestrus, estrus, or diestrus, and some animals were in transition from proestrus to estrus at the time of examination. Keratinized epithelial cells, nucleated epithelial cells, and leukocytes were observed in patterns consistent with the respective stages of the estrous cycle.

### Organ weight

In the male main groups, significant decreases in absolute organ weight were observed for the spleen in G2 and G3, seminal vesicles in G2, and prostate + seminal vesicles in G3 and G4 compared with G1. Significant increases were observed for the liver in G4 and pituitary gland in G2 and G4 compared with G1 (Table 13).

**Table 13:** Absolute Organ Weight(g) of male rats.

Male								
Tissue	Group							
	G1		G2		G3		G4	
	mean±SD	N	mean±SD	N	mean±SD	N	mean±SD	N
Adrenals^c^	0.06 ± 0.00	10	0.06 ± 0.01	10	0.06 ± 0.00	10	0.06 ± 0.01	10
Kidneys^c^	2.74 ± 0.42	10	2.99 ± 0.32	10	2.72 ± 0.25	10	2.98 ± 0.44	10
Liver	11.38 ± 2.14	10	13.33 ± 2.62	10	12.36 ± 1.28	10	14.77 ± 1.79**	10
Heart	1.18 ± 0.11	10	1.23 ± 0.11	10	1.14 ± 0.11	10	1.22 ± 0.14	10
Brain	1.91 ± 0.06	10	1.82 ± 0.17	10	1.73 ± 0.19	10	1.80 ± 0.26	10
Spleen	0.90 ± 0.23	10	0.65 ± 0.14*	10	0.68 ± 0.06*	10	0.90 ± 0.23	10
Epididymis^c^	1.42 ± 0.12	10	1.42 ± 0.33	10	1.41 ± 0.09	10	1.40 ± 0.07	10
Testes^c^	3.00 ± 0.13	10	2.84 ± 0.48	10	2.96 ± 0.10	10	3.16 ± 0.20	10
Thymus	0.41 ± 0.13	10	0.47 ± 0.14	10	0.48 ± 0.14	10	0.52 ± 0.14	10
Thyroid	0.00 ± 0.00	10	0.00 ± 0.00	10	0.00 ± 0.00	10	0.00 ± 0.00	10
Seminal Vesicles	0.20 ± 0.00	10	0.19 ± 0.01	10	0.20 ± 0.00	10	0.19 ± 0.01	10
Prostate + Seminal Vesicles with coagulating glands	1.08 ± 0.00	10	1.07 ± 0.00	10	1.07 ± 0.00*	10	1.07 ± 0.00**	10
Prostate	0.64 ± 0.00	10	0.67 ± 0.02	10	0.64 ± 0.03	10	0.63 ± 0.03	10
Pituitary Glands	0.01 ± 0.00	10	0.01 ± 0.00**	10	0.01 ± 0.00	10	0.01 ± 0.00**	10
**Tissue**	**Group**							
	**G5**				**G6**			
	**mean±SD**		**N**		**mean±SD**			**N**
Adrenals^c^	0.07 ± 0.00		5		0.07 ± 0.01			5
Kidneys^c^	3.40 ± 0.47		5		3.41 ± 0.52			5
Liver	13.10 ± 2.21		5		11.26 ± 0.75			5
Heart	1.30 ± 0.0		5		1.21 ± 0.07			5
Brain	2.08 ± 0.18		5		1.93 ± 0.07			5
Spleen	0.86 ± 0.08		5		0.77 ± 0.11			5
Epididymis^c^	1.43 ± 0.09		5		1.45 ± 0.05			5
Testes^c^	3.04 ± 0.08		5		0.46 ± 0.08			5
Thymus	0.46 ± 0.08		5		0.46 ± 0.08			5
Thyroid	0.00 ± 0.00		5		0.00 ± 0.00			5
Seminal Vesicles	0.47 ± 0.01		5		0.45 ± 0.03			5
Prostate + Seminal Vesicles with coagulating glands	1.07 ± 0.00		5		1.07 ± 0.00			5
Prostate	0.64 ± 0.01		5		0.66 ± 0.03			5
Pituitary Glands	0.01 ± 0.00		5		0.01 ± 0.00			5

^c^Paired organs were weighed together

Liver: **P < 0.05(0.0018) in G4 with significant increase at 95% confidence interval; Spleen: *P < 0.05(0.0103 & 0.0258) in G2 & G3 with significant decrease at 95% confidence interval; Prostate + Seminal Vesicle: * & **P < 0.05(0.0206 & 0.0010) in G3 & G4 with significant decrease at 95% confidence interval; Pituitary Glands: ** & **P < 0.05 (0.0076 & 0.0049) in G2 & G4 with significant increase at 95% confidence interval; Seminal Vesicles: *P < 0.05(0.0138) in G2 with significant decrease at 95% confidence interval. (G2, G3, and G4 are compared with G1)

In the female main groups, significant decreases in absolute organ weight were observed for the spleen in G2 and uterus in G3 compared with G1. Significant increases were observed for the kidneys in G3 and G4, heart in G3, brain in G2 and G3, thymus in G2, and thyroid in G3 compared with G1 (Table 14). No hypertrophy or atrophy of the liver, pituitary gland, spleen, seminal vesicles, kidneys, heart, thymus, brain, thyroid, prostate + seminal vesicles, or uterus was observed on gross examination or histopathology, and no severe histopathological findings were noted in any organ in either sex.

**Table 14:** Absolute Organ Weight(g) of female rats.

Female								
Tissue	Group							
	G1		G2		G3		G4	
	mean±SD	N	mean±SD	N	mean±SD	N	mean±SD	N
Adrenals^c^	0.08 ± 0.01	10	0.08 ± 0.01	10	0.08 ± 0.02	10	0.07 ± 0.01	10
Kidneys^c^	1.71 ± 0.15	10	1.70 ± 0.11	10	1.99 ± 0.26*	10	1.97 ± 0.31*	10
Liver	8.10 ± 0.54	10	8.00 ± 0.79	10	8.21 ± 1.05	10	8.67 ± 1.68	10
Heart	0.82 ± 0.09	10	0.86 ± 0.06	10	1.04 ± 0.19**	10	0.90 ± 0.10	10
Brain	1.59 ± 0.14	10	1.76 ± 0.14*	10	1.78 ± 0.17*	10	1.59 ± 0.13	10
Spleen	0.70 ± 0.11	10	0.49 ± 0.08**	10	0.68 ± 0.15	10	0.72 ± 0.15	10
Ovaries^c^	0.14 ± 0.02	10	0.15 ± 0.02	10	0.15 ± 0.03	10	0.15 ± 0.02	10
Uterus and Cervix	0.68 ± 0.17	10	0.62 ± 0.09	10	0.51 ± 0.12*	10	0.57 ± 0.16	10
Thymus	0.38 ± 0.10	10	0.52 ± 0.11*	10	0.48 ± 0.14	10	0.43 ± 0.10	10
Thyroid	0.00 ± 0.00	10	0.00 ± 0.00	10	0.00 ± 0.00**	10	0.00 ± 0.00	10
Pituitary Glands	0.01 ± 0.00	10	0.01 ± 0.00	10	0.01 ± 0.00	10	0.01 ± 0.00	10
**Tissue**	**Group**							
	**G5**				**G6**			
	**mean±SD**		**N**		**mean±SD**			**N**
Adrenals^c^	0.08 ± 0.02		5		0.09 ± 0.01			5
Kidneys^c^	1.88 ± 0.29		5		1.89 ± 0.10			5
Liver	6.42 ± 1.48		5		8.05 ± 0.88			5
Heart	0.88 ± 0.11		5		0.86 ± 0.07			5
Brain	1.88 ± 0.21		5		1.77 ± 0.18			5
Spleen	0.80 ± 0.12		5		0.71 ± 0.06			5
Ovaries^c^	0.14 ± 0.03		5		0.12 ± 0.01			5
Uterus and Cervix	0.83 ± 0.30		5		0.72 ± 0.15			5
Thymus	0.26 ± 0.09		5		0.18 ± 0.01			5
Thyroid	0.00 ± 0.00		5		0.00 ± 0.00			5
Pituitary Glands	0.01 ± 0.00		5		0.01 ± 0.00			5

^c^Paired organs were weighed together

Kidneys: * & *P < 0.05(0.0189 & 0.0306) in G3 & G4 with significant increase at 95% confidence interval; Heart: **P < 0.05(0.0010) in G3 with significant increase at 95% confidence interval; Brain: * & *P < 0.05(0.0280 & 0.0134) in G2 & G3 with significant increase at 95% confidence interval; Spleen: **P < 0.05(0.0016) in G2 with significant decrease at 95% confidence interval; Uterus: *P < 0.05(0.0269) in G3 with significant increase at 95% confidence interval; Thyroid: **P < 0.05(0.0047) in G3 with significant increase at 95% confidence interval. (G2, G3, and G4 are compared with G1)

In the male main groups, significant decreases in relative organ weights were observed for the spleen in G2 and G3, brain in G3, seminal vesicles in G2 and G4, prostate + seminal vesicles in G2, G3, and G4, and prostate in G4 compared with G1. A significant increase was observed for the liver in G4 when compared with G1 (Table 15). In the female main groups, significant decreases in relative organ weights were observed for the adrenal glands in G4, spleen in G2, and uterus in G3 when compared with G1. Significant increases were observed for the kidneys in G3, heart in G3, brain in G3, thymus in G2, and thyroid in G3 compared with G1 (Table 16). No significant changes were observed in either sex in the recovery groups for absolute or relative organ weights (Tables 13–16).

**Table 15:** Relative Organ Weight (%) of male rats.

Male								
Tissue	Group							
	G1		G2		G3		G4	
	mean±SD	N	mean±SD	N	mean±SD	N	mean±SD	N
Fasted Body Weight (g)	232.80 ± 3.93	10	237.11 ± 2.03	10	239.35 ± 2.54	10	241.76 ± 2.62	10
Adrenals^c^	0.03 ± 0.00	10	0.03 ± 0.00	10	0.02 ± 0.00	10	0.03 ± 0.00	10
Kidneys^c^	1.17 ± 0.17	10	1.26 ± 0.13	10	1.14 ± 0.10	10	1.23 ± 0.18	10
Liver	4.88 ± 0.90	10	5.61 ± 1.07	10	5.16 ± 0.53	10	6.11 ± 0.73**	10
Heart	0.51 ± 0.05	10	0.52 ± 0.05	10	0.48 ± 0.05	10	0.51 ± 0.06	10
Brain	0.82 ± 0.03	10	0.77 ± 0.07	10	0.72 ± 0.08*	10	0.74 ± 0.11	10
Spleen	0.39 ± 0.10	10	0.27 ± 0.06**	10	0.28 ± 0.03*	10	0.37 ± 0.10	10
Epididymis^c^	0.61 ± 0.06	10	0.60 ± 0.14	10	0.59 ± 0.04	10	0.58 ± 0.03	10
Testes^c^	1.29 ± 0.05	10	1.20 ± 0.20	10	1.24 ± 0.04	10	1.31 ± 0.09	10
Thymus	0.18 ± 0.05	10	0.20 ± 0.06	10	0.20 ± 0.06	10	0.22 ± 0.06	10
Thyroid	0.00 ± 0.00	10	0.00 ± 0.00	10	0.00 ± 0.00	10	0.00 ± 0.00	10
Seminal Vesicles	0.09 ± 0.00	10	0.08 ± 0.01	10	0.08 ± 0.00	10	0.08 ± 0.00	10
Prostate + Seminal Vesicles with coagulating glands	0.46 ± 0.01	10	0.45 ± 0.00**	10	0.45 ± 0.00****	10	0.44 ± 0.00****	10
Prostate	0.28 ± 0.00	10	0.28 ± 0.01	10	0.27 ± 0.01	10	0.26 ± 0.01*	10
Pituitary Glands	0.01 ± 0.00	10	0.01 ± 0.00	10	0.01 ± 0.00	10	0.01 ± 0.00	10
**Tissue**	**Group**							
	**G5**				**G6**			
	**mean±SD**			**N**	**mean±SD**			**N**
Fasted Body Weight (g)	251.92 ± 2.98			5	252.46 ± 1.58			5
Adrenals^c^	0.03 ± 0.00			5	0.03 ± 0.00			5
Kidneys^c^	1.35 ± 0.20			5	1.35 ± 0.20			5
Liver	5.21 ± 0.92			5	4.46 ± 0.32			5
Heart	0.52 ± 0.03			5	0.48 ± 0.03			5
Brain	0.83 ± 0.08			5	0.77 ± 0.02			5
Spleen	0.34 ± 0.04			5	0.31 ± 0.04			5
Epididymis^c^	0.57 ± 0.03			5	0.57 ± 0.02			5
Testes^c^	1.21 ± 0.04			5	1.20 ± 0.06			5
Thymus	0.18 ± 0.03			5	0.18 ± 0.03			5
Thyroid	0.00 ± 0.00			5	0.00 ± 0.00			5
Seminal Vesicles	0.19 ± 0.00			5	0.18 ± 0.01			5
Prostate + Seminal Vesicles with coagulating glands	0.43 ± 0.01			5	0.43 ± 0.00			5
Prostate	0.26 ± 0.01			5	0.26 ± 0.01			5
Pituitary Glands	0.01 ± 0.00			5	0.01 ± 0.00			5

^c^Paired organs were weighed together

Liver: **P < 0.05(0.0063) in G4 with significant increase at 95% confidence interval; Spleen: ** & *P < 0.05(0.0059 & 0.0128) in G2 & G3 with significant decrease at 95% confidence interval; Brain: *P < 0.05(0.0264) in G3 with significant decrease at 95% confidence interval; Prostate + Seminal Vesicle: **, **** & ****P < 0.05(0.0030), P < 0.0001 in G2,G3 & G4 respectively with significant decrease at 95% confidence interval; Prostate: *P < 0.05(0.0256) in G4 with significant decrease at 95% confidence interval; Seminal Vesicles: *** & *P < 0.05(0.0007 & 0.0116) in G2 & G4 with significant decrease at 95% confidence interval. (G2, G3, and G4 are compared with G1)

**Table 16:** Relative Organ Weight (%) of female rats.

Female								
Tissue	Group							
	G1		G2		G3		G4	
	mean±SD	N	mean±SD	N	mean±SD	N	mean±SD	N
Fasted Body Weight (g)	221.85 ± 6.46	10	225.19 ± 4.33	10	226.71 ± 1.90	10	225.36 ± 3.97	10
Adrenals^c^	0.04 ± 0.00	10	0.03 ± 0.00	10	0.04 ± 0.01	10	0.03 ± 0.00*	10
Kidneys^c^	0.77 ± 0.06	10	0.76 ± 0.06	10	0.88 ± 0.12*	10	0.88 ± 0.13	10
Liver	3.66 ± 0.03	10	3.55 ± 0.34	10	3.62 ± 0.47	10	3.85 ± 0.75	10
Heart	0.37 ± 0.04	10	0.38 ± 0.03	10	0.46 ± 0.09**	10	0.40 ± 0.04	10
Brain	0.72 ± 0.07	10	0.78 ± 0.06	10	0.79 ± 0.08*	10	0.71 ± 0.05	10
Spleen	0.32 ± 0.05	10	0.22 ± 0.04**	10	0.30 ± 0.07	10	0.32 ± 0.07	10
Ovaries^c^	0.07 ± 0.01	10	0.07 ± 0.01	10	0.07 ± 0.01	10	0.07 ± 0.01	10
Uterus and Cervix	0.31 ± 0.08	10	0.28 ± 0.04	10	0.23 ± 0.05*	10	0.25 ± 0.07	10
Thymus	0.17 ± 0.04	10	0.23 ± 0.05*	10	0.21 ± 0.06	10	0.19 ± 0.04	10
Thyroid	0.00 ± 0.00	10	0.00 ± 0.00	10	0.00 ± 0.00*	10	0.00 ± 0.00	10
Pituitary Glands	0.01 ± 0.00	10	0.01 ± 0.00	10	0.01 ± 0.00	10	0.01 ± 0.00	10
**Tissue**	**Group**							
	**G5**					**G6**		
	**mean±SD**		**N**			**mean±SD**		**N**
Fasted Body Weight (g)	237.32 ± 2.49		5			237.40 ± 2.65		5
Adrenals^c^	0.03 ± 0.01		5			0.04 ± 0.00		5
Kidneys^c^	0.79 ± 0.12		5			0.79 ± 0.05		5
Liver	2.71 ± 0.64		5			3.39 ± 0.40		5
Heart	0.37 ± 0.05		5			0.36 ± 0.03		5
Brain	0.79 ± 0.09		5			0.75 ± 0.07		5
Spleen	0.34 ± 0.05		5			0.30 ± 0.02		5
Ovaries^c^	0.06 ± 0.01		5			0.05 ± 0.01		5
Uterus and Cervix	0.35 ± 0.13		5			0.30 ± 0.06		5
Thymus	0.11 ± 0.04		5			0.08 ± 0.00		5
Thyroid	0.00 ± 0.00		5			0.00 ± 0.00		5
Pituitary Glands	0.01 ± 0.00		5			0.01 ± 0.00		5

^c^Paired organs were weighed together

Adrenals: *P < 0.05(0.0373) in G4 with significant decrease at 95% confidence interval; Kidneys: *P < 0.05(0.0475) in G3 with significant increase at 95% confidence interval; Heart: **P < 0.05(0.0031) in G3 with significant increase at 95% confidence interval; Brain: *P < 0.05(0.0480) in G3 with significant increase at 95% confidence interval; Spleen: **P < 0.05(0.0012) in G2 with significant decrease at 95% confidence interval; Uterus: *P < 0.05(0.0159) in G3 with significant decrease at 95% confidence interval; Thymus: *P < 0.05(0.0350) in G2 with significant increase at 95% confidence interval; Thyroid: *P < 0.05(0.0466) in G3 with significant increase at 95% confidence interval. (G2, G3, and G4 are compared with G1)

Overall, increases and decreases in absolute and relative organ weights were observed sporadically in both directions across the treated groups and did not exhibit a consistent dose-response pattern.

### Histopathology

Histopathological examinations were conducted on G1 and G4, as well as G5 and G6. G2 and G3 were not subjected to histopathological evaluation, as no significant pathological changes were observed in G4 compared to the control group, and the few changes observed were non-specific and minimal in nature. No gross pathologic findings were observed in any of the treated animals compared to the control group. Notably, no significant histopathological changes were observed in G5. The histopathological findings are summarized in Table 17 and Table 18.

**Table 17:** Histopathology Observation of male rats.

Male			
Organ	Finding/Observation	No. of animals showing the histopathological finding per total number of animals examined per group	
		G1	G4
Adrenals	No Abnormality Detected	10/10	10/10
Brain	No Abnormality Detected	10/10	10/10
Heart	No Abnormality Detected	10/10	10/10
Kidney	No Abnormality Detected	9/10	6/10
	Tubular degeneration	1/10	1/10
	Inflammatory cells infiltration	0/10	2/10
	Degeneration of renal tubules	0/10	1/10
Liver	No Abnormality Detected	7/10	4/10
	Inflammatory cells infiltration	1/10	0/10
	Degeneration of hepatocytes	1/10	1/10
	Hemorrhages	1/10	0/10
	Infiltration of inflammatory cells	0/10	5/10
Lungs with bronchi and bronchioles	No Abnormality Detected	8/10	9/10
	Hemorrhages	2/10	0/10
	BALT	0/10	1/10
Testes	No Abnormality Detected	10/10	10/10
Epididymides	No Abnormality Detected	10/10	10/10
Urinary Bladder	No Abnormality Detected	10/10	10/10
Thyroid	No Abnormality Detected	10/10	10/10
Stomach	No Abnormality Detected	10/10	10/10
Spleen	No Abnormality Detected	10/10	10/10
Thymus	No Abnormality Detected	10/10	10/10
Lymph node (Mesenteric)	No Abnormality Detected	10/10	10/10
Skin along with Mammary glands	No Abnormality Detected	10/10	10/10
Small intestine (Duodenum, Jejunum, Terminal Ileum)	No Abnormality Detected	10/10	10/10
Large intestine (Caecum, Colon, rectum)	No Abnormality Detected	10/10	10/10
Prostate	No Abnormality Detected	10/10	10/10
Pituitary Gland	No Abnormality Detected	10/10	10/10
Eye	No Abnormality Detected	10/10	10/10
Esophagus	No Abnormality Detected	10/10	10/10
Aorta	No Abnormality Detected	10/10	10/10
Seminal vesicles	No Abnormality Detected	10/10	10/10
**Organ**	**Finding/Observation**	**No. of animals showing the histopathological finding per total number of animals examined per group**	
		**G5**	**G6**
Adrenals	No Abnormality Detected	5/5	5/5
Brain	No Abnormality Detected	5/5	5/5
Heart	No Abnormality Detected	5/5	5/5
Kidney	No Abnormality Detected	4/5	3/5
	Mononuclear cells infiltration	1/5	0/5
	Hemorrhages	0/5	1/5
	Congestion	0/5	1/5
Liver	No Abnormality Detected	3/5	5/5
	Sinusoidal hemorrhages	1/5	0/5
	Central Vein Congestion	1/5	0/5
Lungs with bronchi and bronchioles	No Abnormality Detected	5/5	5/5
Testes	No Abnormality Detected	5/5	5/5
Epididymides	No Abnormality Detected	5/5	5/5
Urinary Bladder	No Abnormality Detected	5/5	5/5
Thyroid	No Abnormality Detected	5/5	5/5
Stomach	No Abnormality Detected	5/5	5/5
Spleen	No Abnormality Detected	5/5	5/5
Thymus	No Abnormality Detected	5/5	5/5
Lymph node (Mesenteric)	No Abnormality Detected	5/5	5/5
Skin along with Mammary glands	No Abnormality Detected	5/5	5/5
Small intestine (Duodenum, Jejunum, Terminal Ileum)	No Abnormality Detected	5/5	5/5
Large intestine (Caecum, Colon, rectum)	No Abnormality Detected	5/5	5/5
Prostate	No Abnormality Detected	5/5	5/5
Pituitary Gland	No Abnormality Detected	5/5	5/5
Eye	No Abnormality Detected	5/5	5/5
Esophagus	No Abnormality Detected	5/5	5/5
Aorta	No Abnormality Detected	5/5	5/5
Seminal vesicles	No Abnormality Detected	5/5	5/5

**Table 18:** Histopathology Observation of female rats.

Female			
Organ	Finding/Observation	No. of animals showing the histopathological finding per total number of animals examined per group	
		G1	G4
Adrenals	No Abnormality Detected	10/10	10/10
Brain	No Abnormality Detected	10/10	10/10
Heart	No Abnormality Detected	10/10	10/10
Kidneys	No Abnormality Detected	9/10	8/10
	Infiltration of inflammatory cells	1/10	0/10
	Hemorrhages	0/10	1/10
	Tubular Hemorrhages	0/10	1/10
Liver	Congestion	1/10	0/10
	No Abnormality Detected	9/10	8/10
	Central vein congestion	0/10	1/10
	Hemorrhages	0/10	1/10
Lungs with bronchi and bronchioles	No Abnormality Detected	5/10	3/10
	BALT	3/10	6/10
	Lymphocyte infiltration	0/10	1/10
	Hemorrhages	2/10	0/10
Ovaries	No Abnormality Detected	10/10	10/10
Uterus with Cervix	No Abnormality Detected	10/10	10/10
Urinary Bladder	No Abnormality Detected	10/10	10/10
Thyroid	No Abnormality Detected	10/10	10/10
Stomach	No Abnormality Detected	10/10	9/10
	Lymphocyte infiltration	0/10	1/10
Spleen	No Abnormality Detected	10/10	10/10
Thymus	No Abnormality Detected	10/10	10/10
Lymph node (Mesenteric)	No Abnormality Detected	10/10	10/10
Skin along with Mammary glands	No Abnormality Detected	10/10	10/10
Small intestine (Duodenum, Jejunum, Terminal Ileum)	No Abnormality Detected	10/10	10/10
Large intestine (Caecum, Colon, Rectum)	No Abnormality Detected	10/10	10/10
Eye	No Abnormality Detected	10/10	10/10
Esophagus	No Abnormality Detected	10/10	10/10
aorta	No Abnormality Detected	10/10	10/10
**Organ**	**Finding/Observation**	**No. of animals showing the histopathological finding per total number of animals examined per group**	
		**G5**	**G6**
Adrenals	No Abnormality Detected	5/5	5/5
Brain	No Abnormality Detected	5/5	5/5
Heart	No Abnormality Detected	5/5	5/5
Kidneys	No Abnormality Detected	5/5	5/5
Liver	No Abnormality Detected	5/5	5/5
Lungs with bronchi and bronchioles	No Abnormality Detected	5/5	3/5
	Hemorrhages	0/5	1/5
	BALT	0/5	1/5
Ovaries	No Abnormality Detected	5/5	5/5
Uterus with Cervix	No Abnormality Detected	5/5	5/5
Urinary Bladder	No Abnormality Detected	5/5	5/5
Thyroid	No Abnormality Detected	5/5	5/5
Stomach	No Abnormality Detected	5/5	5/5
Spleen	No Abnormality Detected	5/5	5/5
Thymus	No Abnormality Detected	5/5	5/5
Lymph node (Mesenteric)	No Abnormality Detected	5/5	5/5
Skin along with Mammary glands	No Abnormality Detected	5/5	5/5
Small intestine (Duodenum, Jejunum, Terminal Ileum)	No Abnormality Detected	5/5	5/5
Large intestine (Caecum, Colon, Rectum)	No Abnormality Detected	5/5	5/5
Eye	No Abnormality Detected	5/5	5/5
Esophagus	No Abnormality Detected	5/5	5/5
aorta	No Abnormality Detected	5/5	5/5

#### Kidneys.

**Fig 7:**
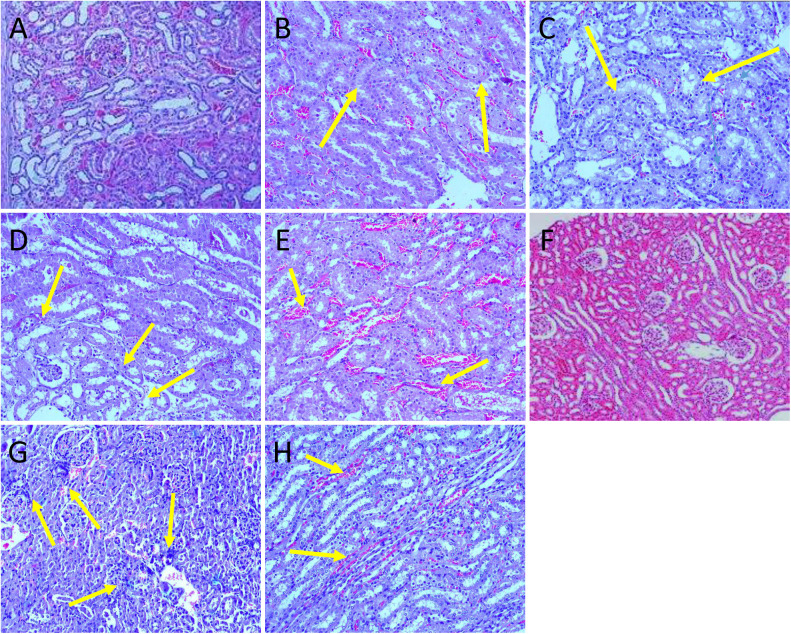
Histopathology photographs of kidneys. All sections were stained with H&E (10x). A: Male G1 with no histopathological changes; B: Male G1 with tubular degeneration; C: Male G4 with tubular degeneration; D: Male G5 with mononuclear cells infiltration; E: Male G6 with hemorrhages; F: Female G1 with no histopathological changes; G: Female G1 with infiltration of inflammatory cells; H: Female G4 with tubular hemorrhages.

In the kidneys, the following minimal and non-specific changes were observed: In main group males, tubular degeneration was noted in G1 (1/10) (Fig 7B). In G4, tubular degeneration (1/10), inflammatory cell infiltration (2/10), and degeneration of renal tubules (1/10) were observed (Fig 7C). In recovery group males, mononuclear cell infiltration was observed in G5 (1/5) (Fig 7D), and hemorrhages (1/5) and congestion (1/5) in G6 (Fig 7E). In main group females, infiltration of inflammatory cells was detected in G1 (1/10) (Fig 7G), while hemorrhages (1/10) and tubular hemorrhages (1/10) were noted in G4 (Fig 7H).

#### Liver.

**Fig 8:**
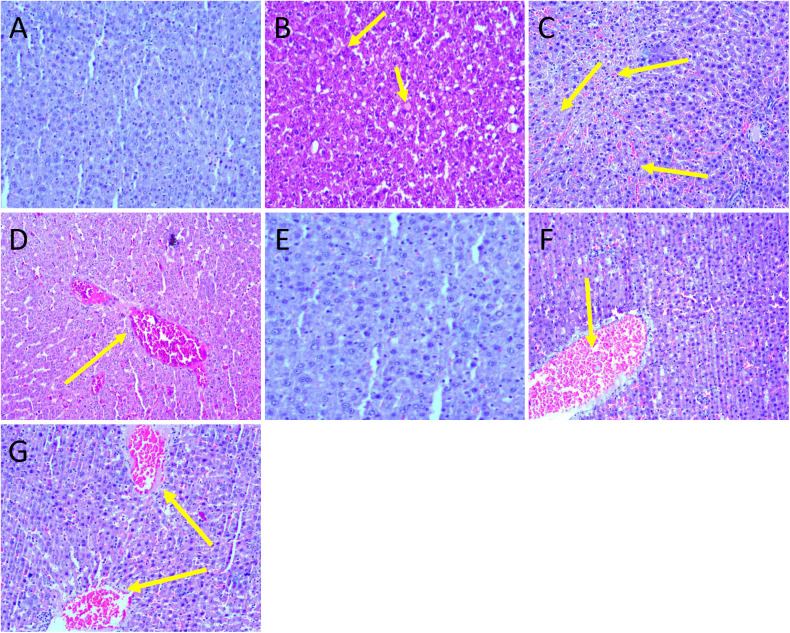
Histopathology photographs of liver. All sections were stained with H&E (10x). A: Male G1 with no histopathological changes; B: Male G1 with degeneration of hepatocytes; C: Male G4 with degeneration of hepatocytes; D: Male G5 with central vein congestion; E: Female G1 with no histopathological changes; F: Female G1 with congestion; G; Female G4 with central vein congestion and hemorrhages.

In the liver, minimal changes included: In main group males, inflammatory cell infiltration (1/10), degeneration of hepatocytes (1/10) and hemorrhages (1/10) were observed in G1 (Fig 8B), while degeneration of hepatocytes (1/10) and infiltration of inflammatory cells (5/10) were noted in G4 (Fig 8C). In recovery group males, sinusoidal hemorrhages (1/5) and central vein congestion (1/5) were detected in G5 (Fig 8D). In main group females, congestion was observed in G1 (1/10) (Fig 8F). In G4, central vein congestion (1/10) and hemorrhages (1/10) were observed (Fig 8G).

#### Lungs.

**Fig 9:**
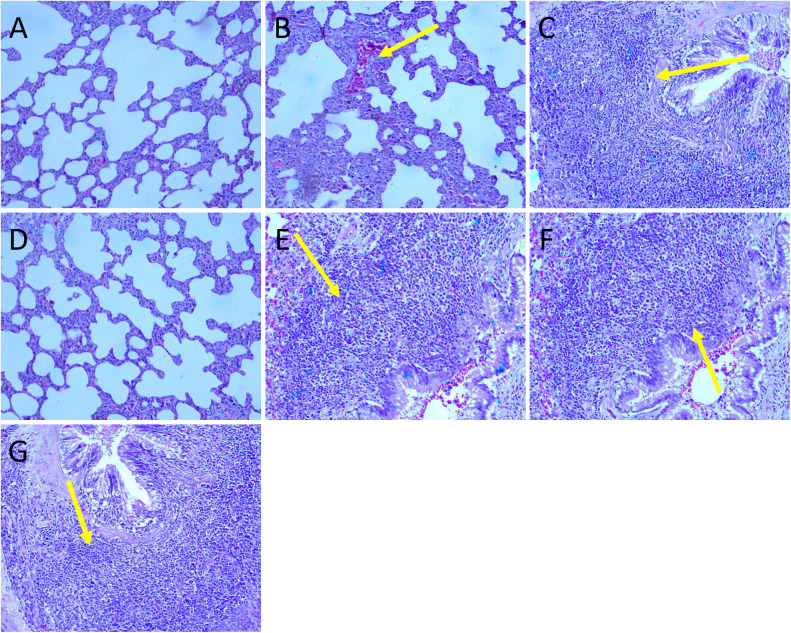
Histopathology photographs of lungs. All sections were stained with H&E (10x). A: Male G1 with no histopathological changes; B: Male G1 with hemorrhages; C: Male G4 with BALT; D: Female G1 with no histopathological changes; E: Female G1 with BALT; F: Female G6 with BALT.

In the lungs, the following changes were observed: In main group males, hemorrhages in lungs with bronchi and bronchioles were noted in G1 (2/10) (Fig 9B), while Bronchus Associated Lymphoid Tissue (BALT) was observed in G4 (1/10) (Fig 9C). In main group females, hemorrhages (2/10) and BALT (3/10) were detected in G1 (Fig 9E). In G4, BALT (6/10) and lymphocyte infiltration (1/10) were observed (Fig 9F). In recovery group females, hemorrhages (1/5) and BALT (1/5) were observed in G6 (Fig 9G). In the stomach, lymphocyte infiltration was observed in G4 females (1/10).

## Discussion

### Body weight

Statistically significant changes in body weight and body weight gain were observed at some time points in both males and females; however, these changes were sporadic, small in magnitude, and showed no consistent dose-related trend. Accordingly, they were not considered to be test-item-related. Body weight increased gradually from Day 1 to termination in all groups, indicating normal growth. No signs of toxicity or treatment-related clinical symptoms were observed during the dosing or recovery periods, and animals appeared normal during weekly detailed clinical observations. Food consumption was comparable among groups, further supporting that the minor variations in body weight were unlikely to be attributable to the test item. Overall, the test item produced no adverse effects on body weight or body weight gain under the conditions of this study.

### Clinical pathology

Although several statistically significant changes were observed in hematological parameters, these findings were sporadic, showed no consistent dose-related trend and were not supported by clinical signs or pathological evidence. Minor fluctuations were noted in leukocyte differentials (e.g., lymphocytes, eosinophils, basophils, monocytes) and reticulocytes; however, the changes were small in magnitude and occurred inconsistently across dose groups. In addition, no gross or histopathological findings suggestive of systemic inflammation or infection were observed, supporting the interpretation that these hematological variations were not adverse and were unlikely to be test item-related. accordingly, the statistically significant differences in hematology were considered within the range of biological variation.

For coagulation parameters, prothrombin time (PT) and activated partial thromboplastin time (APTT) showed occasional statistically significant differences; however, these changes were not dose-dependent and did not show a consistent pattern across dose groups. Moreover, there were no gross or histopathological findings indicative of hemorrhages or organ damage at necropsy that would suggest coagulation-related toxicity. Therefore, the observed coagulation changes were considered not adverse and unlikely to be related to the test item.

In clinical chemistry, although some statistically significant increases or decreases were observed, the changes were inconsistent across dose groups and lacked a dose-related trend. Importantly, there were no corresponding finding in clinical observations, organ weights, gross pathology, or histopathology that would indicate organ toxicity. For example, changes in liver-related parameters (e.g., creatinine and total protein) were not supported by pathological findings in the kidneys. Variations in serum calcium and lipid parameters were also not associated with relevant lesions in the thyroid gland, heart, or major vessels. Taken together, the absence of supportive pathological findings and the inconsistent, non-dose-dependent nature of these biochemical changes indicate that they were not attributable to the test item and were of low toxicological relevance.

### Vaginal cytology

The estrous cycle stages and the cell types observed in vaginal cytology were within expected physiological ranges, suggesting no test item-related effects on estrous cyclicity and no evidence of endocrine disruption within the parameters evaluated.

### Organ weight

Although several statistically significant increases and decreases were observed in absolute and relative organ weights, these variations were sporadic, showed no consistent dose-related trend, and were not accompanied by gross findings suggestive of organ hypertrophy or atrophy. Histopathological examination did not reveal any severe abnormalities or toxicologically relevant lesions in either sex within the tissues evaluated. The absence of corresponding organ-specific pathology, even in organs with statistically significant weight changes, suggests that these differences were incidental or within physiological variation rather than treatment-related. Therefore, the observed organ weight changes were considered non-adverse and unlikely to be related to the test item.

### Histopathology

Although some histopathological findings were observed in several groups, they occurred at low incidence were minimal in severity, showed no dose-related trend, and no relevant differences in gross pathology or clinical signs were noted between treated and control animals. No clinical signs indicative of complications such as ascites or hepatic failure, which could arise secondary to sinusoidal hemorrhages or central vein congestion in the liver, were observed, and serum liver enzyme activities remained within normal biological ranges. In the kidneys, tubular degeneration and mild inflammatory cell infiltration were noted in a small number of animals; however, no hematuria or other clinical manifestations of renal dysfunction were detected, and there were no histopathological features of renal hypertrophy or atrophy. Similarly, focal hemorrhages and inflammatory infiltrates were present in the lungs of some animals, but there were no macroscopic signs of pneumonia or respiratory distress, such as abnormal breathing or nasal discharge. Importantly, most of the pathological changes described above were observed at comparable incidence in both control and high-dose groups, including animals in both the main and recovery periods. These findings were generally minimal in severity, non-specific in nature, and were not accompanied by relevant changes in clinical pathology parameters or organ weights, including in organs such as the spleen where histopathological alterations were observed. Taken together, the histopathological changes are considered to represent spontaneous background lesions and are not related to administration of the test item.

### Overall assessment of systemic toxicity

Overall, several statistically significant differences in body weight gain, organ weight, and clinical pathology parameters were observed sporadically in the low-, mid-, and high-dose groups compared with the control. However, these changes did not follow a consistent dose-response pattern across the four dose levels, were generally small in magnitude, and remained within the expected biological variation for this strain. In addition, they were not supported by corroborating findings in clinical signs, gross necroscopy, vaginal cytology, or histopathology. Therefore, these sporadic changes were not considered toxicologically meaningful or related to administration of SCP Complex-LS.

### Comparison with related studies

The safety of cartilage-derived ingredients has been evaluated in various forms. Our findings are consistent with those of Kudo et al. [6], who reported no toxicity for salmon nasal cartilage powder containing PG. While their study used a limit test with a single high dose (1,000 mg/kg) for 90 days, our study extends these observations to a complex extract containing undenatured collagen and PG, administered daily at doses relevant to human consumption.

A theoretical consideration for undenatured collagen, compared with hydrolyzed collagen, is the potential for immune-mediated reactions due to preservation of higher-order structure. Marone et al. [7] reported no toxicological changes for undenatured type II collagen (chicken sternum) at doses up to 400 mg/kg/day. In our study, although some statistically significant changes in leukocyte differentials (e.g., lymphocytes and eosinophils) were observed, they were sporadic, showed no consistent dose-response, and were not supported by clinical signs or gross/histopathological findings. Notably, no histopathological abnormalities were observed in immune-related organs (spleen, thymus, and mesenteric lymph nodes). These findings suggest that salmon-derived undenatured collagen (SCP Complex-LS) does not raise evidence of systemic immune toxicity within the endpoints evaluated in this 90-day study.

Regarding hydrolyzed collagen, long-term studies such as the 24-month chronic toxicity study by Liang et al. [8] on salmon skin collagen peptides support a high safety profile. Although our study duration (90 days) and dose levels were lower than those in chronic studies, the absence of adverse findings aligns with the broader safety consensus for collagen-derived products. A divergence from previous high-dose studies [6,7] is the dose range: we focused on a multiple of the estimated human intake (approximately 25-fold margin) rather than determining a maximum tolerated dose. Nevertheless, the lack of toxicity in our study, together with high-dose safety data from existing literature [6–10], collectively supports the safety of SCP Complex-LS.

### Limitations and future directions

This study has several limitations that should be considered when interpreting the findings. First, the 90-day duration, while compliant with OECD Test Guideline 408 for subchronic toxicity assessment, does not address potential effects of lifetime exposure or transgenerational impacts. Second, although a comprehensive panel of toxicological endpoints was evaluated, specialized studies of reproductive and developmental toxicity, immunotoxicity, genotoxicity, and carcinogenicity, and chronic toxicity were not conducted. Third, the study was conducted in a single species (Sprague-Dawley rats) using oral gavage; potential species-specific responses or effects via other exposure routes were not examined. Finally, these findings apply specifically to SCP Complex-LS and may not be generalizable to other cartilage-derived formulations.

Despite the established NOAEL of 41.2 mg/kg B.wt./day (approximately 25-fold higher than the estimated human exposure), further studies would strengthen the safety database for SCP Complex-LS. These include chronic toxicity studies to evaluate long-term safety, reproductive and developmental toxicity assessments in accordance with ICH guidelines, and post-marketing surveillance in human populations consuming SCP Complex-LS at intended intake levels. Such investigations would provide additional confidence in the safety margin and support broader applications of this ingredient in functional foods and dietary supplements.

## Conclusion

The results of this 90-day repeated toxicity study indicate that no systemic toxicity or adverse effects were observed at doses up to 41.2 mg/kg B.wt./day when SCP Complex-LS was administered orally to Sprague-Dawley rats under the conditions of this study. In addition, findings from the recovery groups demonstrated no evidence of systemic toxicity or reversible or delayed toxicity at the high dose (41.2 mg/kg B.wt./day). Consequently, the NOAEL for SCP Complex-LS under the present experimental conditions was determined to be 41.2 mg/kg B.wt./day, which corresponds to approximately 25-fold higher exposure than the estimated human intake.

Taken together with previous subchronic toxicity studies of hydrolyzed collagen products at substantially higher dose levels, these findings provide robust scientific evidence supporting the safety of SCP Complex-LS within the practical range of human consumption. Nonetheless, in line with the limitations outlined above, additional studies such as chronic toxicity and reproductive and developmental toxicity assessments, as well as long-term clinical safety evaluations, would be valuable to further refine the safety margin and support broader applications of SCP Complex-LS as a functional food ingredient.

## Supporting information

S1 Text SCP Complex-LS Certificate of Analysis. (PDF)

S2 Text SCP Complex-LS Nutritional Analysis. (PDF)

S3 Data set The underlying data for Fig 2 to 6. (PDF)

S4 Data set The underlying data for Table 6 to Table 9. (PDF)

S5 Data set The underlying data for Table 11 to Table 16. (PDF)
