# Supplementary material for: 90-day oral toxicity study of a salmon nasal cartilage extract containing undenatured collagen and proteoglycan in Sprague-Dawley rats
Source: PLoS One. 2026 Jan 23;21(1):e0340675. doi: 10.1371/journal.pone.0340675 (PMC12829970; doi:10.1371/journal.pone.0340675)
Supplement: S1 Text — (PDF) [file pone.0340675.s001.pdf]

Date of Issue 26-Jun-23

Manufacture and Sales :

LINISE Co., Ltd.

Hokkaido Univ. Global Research Center
for Food & Medical Innovation 405,
Kita21, Nishi11,Kita-ku, Sapporo,
Hokkaido,
001-0021, Japan

Certificate of Analysis

**Product Name**	SCP Complex-LS
**Labeling Name**	Salmon Nasal Cartilage Extract<br>(Contains Undenatured TypeⅡ & Type Ⅺ Collagen, Undenatured Proteoglycan)
**Raw Material**	Salmon Nasal Cartilage
**Origin**	Hokkaido,Japan
**Use**	Food ingredient
**Manufacturing Date**	26-Jun-23
**Expiry Date**	25-Jun-26 (unopened)
**Lot No.**	SCP-AN061-004

**Manager**	**Confirmor**	**Tester**
QC<br>26-Jun-23<br>Okazaki	QC<br>26-Jun-23<br>Sudo	QC<br>26-Jun-23<br>Iwabuchi

Manufacturing Factory

Oshamambe Factory
159-9, Asahihama, Oshamambe,
Hokkaido, 049-3519, Japan

Certifications

Test items	Standard	Lower limit of quantitation	Results
**Property**	White or lemon-yellow powder,<br>Slightly unique smell	-	Confirmed
**pH**	6.5 to 8.5	-	7.8
**Loss on drying**	10.0 % or less	-	1.1
**Residue on ignition**	25.0 % or less	-	13.2
**Undenatured type Ⅱ&type Ⅺ Collagen**	40.0 % or more	-	40.2
**Undenatured Proteoglycan**	40.0 % or more	-	44.2
**Molecular weight (Undenatured Proteoglycan)**	2,000 to 4,150 kDa	-	2,606
**Lead**	2.0 ppm or less	0.1 ppm	ND
**Mercury**	0.5 ppm or less	0.01 ppm	ND
**Cadmium**	0.3 ppm or less	0.05 ppm	ND
**Arsenic**	2.0 ppm or less	0.1 ppm	ND
**Total Aerobic Microbial Count**	3,000 CFU/g or less	-	<300
**Coliforms**	Absent	-	Absent
**Total Yeasts & Mold Count**	100 CFU/g or less	-	<100

Note

Storage: Store in a cool dark place, away from heat and moisture.
Use promptly after opening.

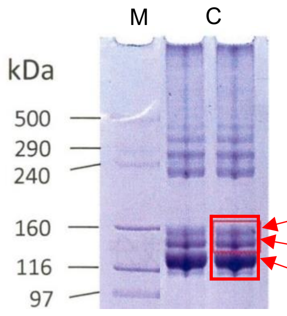

The results of SDS-PAGE confirmed that collagen in cartilage consists of type Ⅱ and type Ⅺ ,and the ratio of type Ⅱ and type Ⅺ collagen in total collagen is about " 8 : 2 ". **(Patent No. 7138873)**

Type Ⅺ collagen-related bands

Upper band :**α1 (Ⅺ)**

Middle band :**α2 (Ⅺ)**

α1(Ⅱ) and Lower band : **α3 (Ⅺ)**
