# Supplementary material for: 90-day oral toxicity study of a salmon nasal cartilage extract containing undenatured collagen and proteoglycan in Sprague-Dawley rats
Source: PLoS One. 2026 Jan 23;21(1):e0340675. doi: 10.1371/journal.pone.0340675 (PMC12829970; doi:10.1371/journal.pone.0340675)
Supplement: S2 Text — (PDF) [file pone.0340675.s002.pdf]

Date of issue 6/26/2023
Date created 6/26/2023

LINISE Co.,Ltd.
Hokkaido Univ. Global Research Center
for Food & Medical Innovation 405,
Kita21, Nishi11, Kita-ku, Sapporo,
Hokkaido, 001-0021, Japan

Nutritional Profile Information

This is to certify the nutritional information of our food products material as follows.

Sample name: SCP Complex-LS

Test Item	Result (/ 100g)	Units
**Energy**	341	kcal
**Moisture**	1.4	g
**Protein**	63.8	g
**Fat**	0.4	g
**Carbohydrate**	20.6	g
**Ash**	13.8	g
**Sodium**	1070	mg
**Salt equivalent (calculated from sodium content)**	2.71	g
