# Supplementary material for: 90-day oral toxicity study of a salmon nasal cartilage extract containing undenatured collagen and proteoglycan in Sprague-Dawley rats
Source: PLoS One. 2026 Jan 23;21(1):e0340675. doi: 10.1371/journal.pone.0340675 (PMC12829970; doi:10.1371/journal.pone.0340675)
Supplement: S3 Data set — (PDF) [file pone.0340675.s003.pdf]

Individual Animal Body Weight (g)

Group: G1 (Vehicle: 0 mg/kg B.wt./day) Sex: Male

Animal Number	Days										
	1	8	15	22	29	36	43	50	57	64	71
1	101.02	103.02	120.02	125.54	131.01	153.02	169.02	175.02	184.02	192.02	201.02
2	105.32	107.65	124.21	129.21	136.02	156.02	172.02	179.69	182.54	194.25	205.65
3	107.02	109.02	126.02	131.09	137.02	158.02	178.02	180.01	186.32	192.66	204.03
4	107.65	107.98	125.02	130.21	135.65	158.99	176.02	185.02	189.66	195.66	206.99
5	108.02	108.96	129.65	135.65	139.65	158.65	179.68	186.65	192.02	198.02	207.02
6	109.65	110.02	130.02	138.01	145.65	159.02	177.54	187.66	195.54	202.12	210.21
7	109.68	110.01	129.02	135.65	142.32	159.65	175.02	189.68	194.25	203.32	213.32
8	110.65	111.02	125.89	129.65	135.65	158.02	176.65	187.90	194.02	205.02	211.02
9	111.32	111.98	130.02	135.65	145.02	158.69	179.99	185.02	189.78	198.99	205.98
10	116.32	117.02	129.02	136.32	146.51	159.99	180.02	189.65	196.65	205.02	214.54

Animal Number	Days		
	78	85	90
1	211.02	220.01	225.65
2	213.21	223.35	229.05
3	215.02	224.02	231.02
4	216.99	229.65	234.54
5	218.02	227.54	234.02
6	216.02	228.02	236.65
7	220.32	228.54	235.02
8	219.99	226.56	236.66
9	218.02	227.00	234.21
10	219.86	227.45	235.02

Individual Animal Body Weight (g)

Group: G2 (Low dose: 10.3 mg/kg B.wt./day) Sex: Male

Animal Number	Days										
	1	8	15	22	29	36	43	50	57	64	71
21	102.02	104.02	119.65	125.02	132.02	148.98	181.02	185.02	192.02	204.02	212.32
22	104.02	104.98	120.02	126.96	135.65	156.36	176.02	187.02	197.25	206.65	215.89
23	107.02	108.02	121.45	128.02	135.65	158.02	178.99	188.65	195.02	205.02	217.85
24	107.98	108.65	123.02	128.98	136.98	158.02	178.02	187.98	192.21	209.99	215.65
25	108.02	108.99	127.02	131.21	139.85	160.02	179.89	186.02	196.36	206.66	219.99
26	109.14	110.02	125.02	132.01	138.02	158.02	176.02	187.02	192.02	208.02	218.54
27	110.02	111.24	129.32	135.65	141.54	159.66	182.02	189.65	194.54	205.02	217.02
28	111.02	111.54	130.01	135.02	140.02	159.02	181.01	186.54	192.32	209.99	215.02
29	112.02	113.05	129.65	136.32	146.65	161.21	183.65	189.99	194.58	204.55	215.65
30	114.02	114.78	130.54	135.02	142.02	159.02	178.02	187.02	195.65	208.02	218.02

Animal Number	Days		
	78	85	90
21	221.02	225.02	238.55
22	223.02	229.89	235.45
23	224.12	230.12	236.50
24	219.89	228.54	234.15
25	226.36	235.02	240.00
26	225.98	229.86	235.02
27	226.32	232.21	238.65
28	224.87	229.78	236.02
29	226.98	232.21	238.55
30	227.58	231.02	239.78

Individual Animal Body Weight (g)

Group: G3 (Mid Dose: 20.6 mg/kg B.wt./day) Sex: Male

Animal Number	Days										
	1	8	15	22	29	36	43	50	57	64	71
41	104.02	105.02	125.02	132.01	139.65	158.65	178.86	185.02	192.02	209.99	215.66
42	104.02	105.68	126.32	132.01	140.01	159.66	179.02	186.65	196.65	210.01	217.45
43	105.54	105.88	124.02	129.35	138.02	162.02	182.02	189.65	195.02	206.66	219.65
44	108.02	108.65	129.02	135.65	142.32	163.21	181.45	187.02	194.02	208.98	214.54
45	108.54	109.02	127.32	132.54	143.02	158.02	176.02	185.02	192.45	208.02	219.99
46	109.32	110.54	128.02	135.65	147.21	158.99	182.02	186.65	195.02	211.25	217.58
47	109.65	110.02	129.36	136.02	145.65	157.02	181.01	187.02	189.65	201.54	216.66
48	111.02	111.65	130.02	137.54	147.02	160.01	178.99	185.02	189.45	201.02	218.64
49	113.02	115.02	130.01	135.01	147.99	162.02	178.54	187.66	193.02	209.99	218.54
50	114.21	115.98	128.65	136.02	148.02	162.99	181.01	186.78	195.54	208.54	215.65

Animal Number	Days		
	78	85	90
41	223.02	230.01	235.02
42	224.02	234.54	239.87
43	228.65	235.65	242.12
44	227.65	232.21	238.87
45	226.35	235.03	239.86
46	226.65	236.45	243.54
47	226.35	231.21	237.45
48	228.58	234.02	241.21
49	225.66	229.78	236.50
50	227.02	234.54	239.87

Individual Animal Body Weight (g)

Group: G4 (High Dose: 41.2 mg/kg B.wt./day) Sex: Male

Animal Number	Days										
	1	8	15	22	29	36	43	50	57	64	71
61	104.02	105.02	130.01	134.02	139.99	156.02	179.98	186.02	192.02	208.65	213.02
62	105.02	106.65	129.02	135.21	142.65	159.98	178.87	187.54	194.54	209.02	215.02
63	107.02	108.02	128.21	133.24	143.02	162.02	182.02	189.99	195.65	210.01	219.99
64	107.02	108.32	129.35	134.54	147.02	158.65	181.01	189.65	194.54	209.89	216.65
65	108.65	109.02	127.02	135.68	148.98	159.69	183.32	191.02	196.36	207.58	218.05
66	109.54	110.21	128.02	136.02	149.99	161.21	178.98	186.05	192.24	210.36	218.02
67	109.65	110.98	130.01	136.08	148.02	160.02	178.05	187.54	196.58	211.02	221.21
68	110.02	111.04	130.66	137.40	147.87	163.62	181.01	192.32	198.89	209.84	218.65
69	113.32	114.54	131.02	138.02	148.98	160.02	180.12	186.02	193.65	205.02	217.15
70	114.65	115.65	128.02	139.99	148.01	162.21	178.98	187.54	15.45	206.66	219.66

Animal Number	Days		
	78	85	90
61	227.85	235.65	239.66
62	226.02	234.12	238.88
63	224.02	235.02	242.21
64	228.98	235.21	243.56
65	227.54	236.65	244.02
66	226.78	237.58	246.65
67	228.56	237.54	239.87
68	225.02	234.02	238.60
69	226.65	234.21	241.02
70	227.02	235.02	243.35

Individual Animal Body Weight (g)

Group: G5 (Recovery Vehicle: 0 mg/kg B.wt./day) Sex: Male

Animal Number	Days									
	1	8	15	22	29	36	43	50	57	64
81	121.02	123.02	128.02	135.96	147.21	158.65	182.02	189.65	192.02	201.36
82	121.02	122.54	129.65	137.02	148.02	159.99	178.58	187.32	195.54	204.55
83	122.42	124.56	130.02	138.20	149.87	161.02	179.33	185.02	192.12	210.32
84	122.63	123.45	131.02	136.45	147.02	162.32	182.01	189.54	195.65	211.02
85	123.02	124.02	132.21	138.02	147.01	164.02	184.02	189.65	194.54	214.78

Animal Number	Days					
	71	78	85	92	99	104
81	210.02	221.21	230.01	235.11	242.08	248.16
82	209.89	226.86	235.02	239.01	245.21	251.11
83	219.66	225.02	236.45	239.54	246.08	253.06
84	218.54	229.78	237.45	241.11	249.81	254.19
85	220.01	229.45	236.66	241.39	250.26	255.11

Group: G6 (Recovery High Dose: 41.2 mg/kg B.wt./day) Sex: Male

Animal Number	Days									
	1	8	15	22	29	36	43	50	57	64
91	119.02	121.02	130.01	139.65	148.98	158.02	179.99	186.02	196.35	210.11
92	120.02	122.32	131.21	139.99	147.02	159.99	182.02	189.99	194.54	208.59
93	121.32	123.02	129.65	140.01	143.65	160.01	181.21	186.65	198.54	210.33
94	123.02	124.54	128.65	135.21	148.02	161.21	179.68	188.02	196.02	214.55
95	124.02	125.65	131.21	136.02	149.02	162.02	184.99	189.65	194.75	210.25

Animal Number	Days					
	71	78	85	92	99	104
91	219.66	223.02	234.02	239.16	246.27	253.41
92	215.02	228.99	238.54	241.15	250.27	256.10
93	218.99	227.54	234.54	239.16	246.98	252.07
94	217.02	226.35	234.54	241.01	249.25	254.41
95	220.32	228.54	235.02	239.31	245.29	251.01

Individual Animal Body Weight (g)

Group: G1 (Vehicle: 0 mg/kg B.wt./day) Sex: Female

Animal Number	Days										
	1	8	15	22	29	36	43	50	57	64	71
11	102.02	105.02	110.02	119.54	125.02	142.65	150.01	161.21	165.98	173.02	185.99
12	105.32	106.65	114.21	120.01	127.01	149.02	152.66	165.02	172.02	179.66	192.02
13	104.66	105.69	112.01	121.11	128.55	147.02	153.24	167.02	175.66	182.54	196.54
14	106.02	107.02	119.65	126.65	132.21	148.21	154.02	169.66	174.02	186.02	192.02
15	107.02	109.02	118.02	129.02	135.02	147.02	156.32	168.90	176.65	186.11	198.99
16	111.02	114.02	122.32	129.99	136.33	148.02	157.02	168.01	174.02	187.25	196.66
17	110.32	112.32	124.02	129.02	137.02	147.00	154.02	167.55	175.21	189.60	198.78
18	112.02	114.02	124.32	130.01	138.99	142.32	157.22	169.99	173.21	180.12	192.02
19	113.21	113.98	126.02	136.54	142.21	142.66	157.98	168.02	174.54	183.21	194.55
20	114.54	115.98	124.21	130.01	135.02	146.65	154.02	167.22	175.66	179.89	190.02

Animal Number	Days		
	78	85	90
11	192.65	201.02	210.02
12	199.89	205.66	213.32
13	205.65	213.54	219.58
14	204.02	216.56	225.25
15	208.02	218.57	224.65
16	209.99	217.45	223.02
17	208.54	219.87	225.78
18	207.25	220.01	226.98
19	206.66	218.75	228.54
20	208.54	215.66	224.02

Individual Animal Body Weight (g)

Group: G2 (Low dose: 10.3 mg/kg B.wt./day) Sex: Female

Animal Number	Days										
	1	8	15	22	29	36	43	50	57	64	71
31	103.65	104.89	111.01	121.01	129.02	142.02	149.99	159.99	165.02	173.52	189.02
32	104.98	105.02	113.21	122.33	129.99	142.33	157.02	168.02	173.32	179.86	187.88
33	108.02	109.69	115.02	125.02	132.02	140.01	157.99	167.22	175.02	183.32	193.25
34	108.98	110.02	119.21	125.65	132.02	145.65	156.00	164.21	176.11	185.66	192.65
35	108.87	109.65	120.01	132.21	139.65	149.65	154.02	165.02	173.56	184.02	192.02
36	109.54	110.02	121.01	129.56	135.02	150.01	156.36	168.99	174.25	189.66	198.99
37	111.02	111.02	121.55	129.02	136.65	150.66	157.02	167.45	176.45	185.75	195.02
38	111.42	112.65	122.01	131.01	138.02	152.21	157.66	168.02	172.02	179.98	192.65
39	114.21	116.02	122.87	130.01	139.65	147.58	156.02	168.02	171.66	182.02	193.56
40	115.65	117.65	122.99	129.66	135.02	148.02	154.32	175.02	173.56	183.21	196.66

Animal Number	Days		
	78	85	90
31	199.88	206.56	216.68
32	198.78	208.99	218.55
33	209.99	219.89	226.65
34	208.28	215.54	228.78
35	207.45	218.57	229.65
36	206.60	216.25	225.02
37	208.78	218.78	227.02
38	209.99	216.36	228.66
39	211.22	218.78	224.54
40	212.66	220.112	226.54

Individual Animal Body Weight (g)

Group: G3 (Mid Dose: 20.6 mg/kg B.wt./day) Sex: Female

Animal Number	Days										
	1	8	15	22	29	36	43	50	57	64	71
51	107.02	109.02	114.54	131.21	135.02	153.21	157.02	168.65	175.24	185.54	195.66
52	105.32	107.25	116.02	125.21	130.01	154.02	159.99	169.02	176.55	184.55	198.02
53	105.98	108.02	117.12	129.66	136.32	152.01	157.54	168.78	174.02	180.25	189.69
54	109.02	112.32	117.89	130.12	138.02	153.45	154.02	164.02	179.66	186.58	195.02
55	109.32	110.02	119.65	125.65	132.21	157.02	156.98	168.00	174.02	185.66	196.66
56	110.02	112.02	120.01	129.02	136.02	147.24	157.02	167.02	173.21	182.01	192.02
57	110.12	112.02	120.32	129.99	135.65	149.65	157.08	169.66	175.24	183.32	198.02
58	112.02	113.02	120.99	131.01	137.02	152.02	158.65	168.02	174.98	184.02	197.88
59	113.89	114.54	122.02	129.65	132.32	157.25	161.24	168.00	173.02	186.66	196.66
60	114.89	115.06	122.54	130.01	136.02	154.66	157.02	167.02	174.54	182.02	195.02

Animal Number	Days		
	78	85	90
51	209.99	216.66	225.45
52	210.65	217.87	226.65
53	207.40	215.66	227.58
54	210.66	218.78	229.89
55	215.02	220.12	225.41
56	209.99	218.66	226.65
57	207.58	218.02	228.54
58	209.99	217.88	226.02
59	215.55	219.66	224.05
60	218.47	223.54	228.57

Individual Animal Body Weight (g)

Group: G4 (High Dose: 41.2 mg/kg B.wt./day) Sex: Female

Animal Number	Days										
	1	8	15	22	29	36	43	50	57	64	71
71	105.21	107.02	119.02	129.65	135.02	153.32	159.99	168.66	173.56	179.86	189.65
72	106.21	107.54	120.01	129.65	136.65	157.02	162.21	169.96	178.02	186.56	196.66
73	108.02	109.02	121.11	129.69	134.12	156.02	162.32	168.02	174.55	182.54	195.02
74	107.98	108.32	121.58	130.12	138.02	158.02	164.02	159.45	176.36	186.35	196.33
75	109.65	110.21	121.99	131.22	136.65	152.02	168.00	175.02	176.65	185.02	196.66
76	110.21	112.02	122.01	132.65	138.02	153.65	159.98	165.02	174.02	186.65	198.25
77	110.02	112.01	122.89	135.02	139.69	154.02	167.58	178.03	176.00	182.45	195.25
78	111.36	113.21	122.98	136.02	142.02	154.99	165.02	172.54	179.02	185.02	199.66
79	114.25	115.02	122.99	137.87	143.65	157.87	167.99	170.01	175.02	186.57	195.02
80	115.86	116.65	122.78	139.69	145.02	158.02	168.02	176.65	182.02	189.89	197.87

Animal Number	Days		
	78	85	90
71	196.68	208.02	215.66
72	209.85	216.56	226.54
73	212.23	218.55	228.02
74	208.45	218.55	225.45
75	217.55	219.66	223.21
76	219.25	225.25	229.45
77	210.02	218.66	224.65
78	214.55	218.99	227.02
79	218.66	223.33	229.36
80	208.59	219.56	224.54

Individual Animal Body Weight (g)

Group: G5 (Recovery Vehicle: 0 mg/kg B.wt./day) Sex: Female

Animal Number	Days									
	1	8	15	22	29	36	43	50	57	64
86	122.21	124.02	130.01	139.02	145.02	158.02	168.02	175.65	182.01	189.65
87	122.45	124.02	131.00	138.02	146.32	157.02	167.25	178.02	183.65	186.02
88	123.54	125.32	131.54	139.54	145.02	154.55	165.02	178.99	187.12	192.32
89	123.02	124.02	131.84	138.02	148.54	153.02	167.02	175.02	179.89	186.55
90	124.54	125.65	132.01	139.50	147.02	154.98	166.68	179.99	185.02	195.02

Animal Number	Days					
	71	78	85	92	99	104
86	196.65	213.32	220.01	224.15	229.68	234.16
87	198.55	215.88	225.25	227.61	235.71	239.01
88	206.02	218.02	225.36	228.54	234.51	237.56
89	198.55	219.66	223.02	226.01	231.31	238.81
90	208.88	218.25	224.55	227.11	232.55	239.41

Group: G6 (Recovery High Dose: 41.2 mg/kg B.wt./day) Sex: Female

Animal Number	Days									
	1	8	15	22	29	36	43	50	57	64
96	120.02	122.56	129.02	138.02	146.02	157.02	164.25	175.02	179.99	186.02
97	122.02	24.02	129.65	138.99	147.58	154.66	168.02	176.02	181.21	188.99
98	121.98	122.02	128.54	137.02	145.02	158.02	164.02	175.45	180.65	186.02
99	124.02	125.32	130.12	139.02	147.98	157.99	167.99	179.02	184.02	192.54
100	125.32	125.99	132.21	138.99	148.65	158.45	167.02	175.22	179.85	180.25

Animal Number	Days					
	71	78	85	92	99	104
96	198.88	214.02	223.66	226.61	232.08	241.01
97	196.66	210.02	220.45	223.91	230.81	236.41
98	195.54	214.22	221.25	224.16	231.57	236.01
99	201.22	215.66	219.66	226.15	234.58	240.11
100	199.86	213.33	220.32	226.11	233.09	236.11

Individual Animal Body Weight Gain (%)

Group: G1 (Vehicle: 0 mg/kg B.wt./day) Sex: Male

Animal Number	Days									
	8	15	22	29	36	43	50	57	64	71
1	1.98	18.81	24.27	29.69	51.47	67.31	73.25	82.16	90.08	98.99
2	2.21	17.94	22.68	29.15	48.14	63.33	70.61	73.32	84.44	95.26
3	1.87	17.75	22.49	28.03	47.65	66.34	68.20	74.10	80.02	90.65
4	0.31	16.14	20.96	26.01	47.69	63.51	71.87	76.18	81.76	92.28
5	0.87	20.02	25.58	29.28	46.87	66.34	72.79	77.76	83.32	91.65
6	0.34	18.58	25.86	32.83	45.03	61.92	71.14	78.33	84.33	91.71
7	0.30	17.63	23..68	29.76	45.56	59.57	72.94	77.11	85.38	94.49
8	0.33	13.77	17.17	22.59	42.81	59.65	69.81	73.35	85.29	90.71
9	0.59	16.80	21.86	30.27	42.55	61.69	66.21	70.48	78.75	90.71
10	0.60	10.92	17.19	25.95	37.54	54.76	63.04	69.06	76.26	84.44

Animal Number	Days		
	78	85	90
1	108.89	117.79	123.37
2	102.44	112.07	117.48
3	100.92	109.33	115.87
4	101.57	113.33	117.87
5	101.83	110.65	116.65
6	94.01	107.95	115.82
7	100.88	108.37	114.28
8	98.82	104.75	113.88
9	95.85	103.92	110.39
10	89.01	95.54	102.05

Individual Animal Body Weight Gain (%)

Group: G2 (Low dose: 10.3 mg/kg B.wt./day) Sex: Male

Animal Number	Days									
	8	15	22	29	36	43	50	57	64	71
21	1.96	17.28	22.54	29.41	46.03	77.44	81.36	88.22	99.98	108.12
22	0.92	15.38	22.05	30.41	50.32	69.22	79.79	89.63	98.66	107.55
23	0.93	13.48	19.62	26.75	47.65	67.25	76.28	82.23	91.57	103.56
24	0.62	13.93	19.45	26.86	46.34	64.86	74.09	78.01	94.47	99.71
25	0.90	17.59	21.47	29.47	48.14	66.53	72.21	81.78	91.32	103.66
26	0.81	14.55	20.95	26.46	44.79	61.28	71.36	75.94	90.60	100.24
27	1.11	17.54	23.30	28.65	45.12	65.44	72.38	76.82	86.35	97.26
28	0.47	17.11	21.62	26.12	43.24	63.04	68.02	73.23	89.15	93.68
29	0.92	15.74	21.69	30.91	43.91	63.94	69.60	73.70	82.60	92.51
30	0.67	14.49	18.42	24.56	39.47	56.13	64.02	71.59	82.44	91.21

Animal Number	Days		
	78	85	90
21	116.64	120.56	133.83
22	114.40	121.01	126.35
23	109.42	115.03	120.99
24	103.64	111.65	116.85
25	109.55	117.57	122.18
26	107.06	110.61	115.34
27	105.71	111.06	116.92
28	102.55	106.97	112.59
29	102.62	107.29	112.95
30	99.60	102.61	110.30

Individual Animal Body Weight Gain (%)

Group: G3 (Mid Dose: 20.6 mg/kg B.wt./day) Sex: Male

Animal Number	Days									
	8	15	22	29	36	43	50	57	64	71
41	0.96	20.19	26.91	34.25	52.52	71.95	77.87	84.60	101.87	107.33
42	1.60	21.44	26.91	34.60	53.49	72.10	79.44	89.05	101.89	109.05
43	0.32	17.51	22.56	30.78	53.52	72.47	79.69	84.78	95.81	108.12
44	0.58	19.44	25.58	31.75	51.09	67.98	73.13	79.61	93.46	98.61
45	0.44	17.30	22.11	31.77	45.59	62.17	70.46	77.31	91.65	102.68
46	1.12	17.11	24.09	34.66	45.44	66.50	70.74	78.39	93.24	99.03
47	0.34	17.98	24.05	32.83	43.20	65.08	70.56	72.96	83.80	97.59
48	0.57	17.11	23.89	32.43	44.13	61.22	66.65	70.64	81.07	96.94
49	1.77	15.03	19.46	30.94	43.36	57.97	66.04	70.78	85.80	93.36
50	1.55	12.64	19.10	29.60	42.71	58.49	63.54	71.21	82.59	88.82

Animal Number	Days		
	78	85	90
41	114.40	121.12	125.94
42	115.36	125.48	130.60
43	116.65	123.28	129.41
44	110.75	114.97	121.13
45	108.54	116.54	120.99
46	107.33	116.29	122.78
47	106.43	110.86	116.55
48	105.89	110.79	117.27
49	99.66	103.31	109.25
50	98.77	105.36	110.03

Individual Animal Body Weight Gain (%)

Group: G4 (High Dose: 41.2 mg/kg B.wt./day) Sex: Male

Animal Number	Days									
	8	15	22	29	36	43	50	57	64	71
61	0.96	24.99	28.84	34.58	49.99	73.02	78.83	84.60	100.59	104.79
62	1.55	22.85	28.75	35.83	52.33	70.32	78.58	85.24	99.03	104.74
63	0.93	19.80	24.50	33.64	51.39	70.08	77.53	82.82	96.23	105.56
64	1.21	20.87	25.71	37.38	48.24	69.14	77.21	81.78	96.12	102.44
65	0.34	16.91	24.88	37.12	46.98	68.73	75.81	80.73	91.05	100.69
66	0.61	16.87	24.17	36.93	47.17	63.39	69.85	75.50	92.04	99.03
67	1.21	18.57	24.10	34.99	45.94	62.38	71.04	79.28	92.45	101.74
68	0.93	18.76	24.89	34.40	48.72	64.52	74.80	80.78	90.73	98.74
69	1.08	15.62	21.80	31.47	41.21	58.95	64.15	70.89	80.92	91.63
70	0.87	11.66	22.10	29.10	41.48	56.11	63.58	70.48	80.25	91.59

Animal Number	Days		
	78	85	90
61	119.04	126.54	130.40
62	115.22	122.93	127.46
63	109.33	119.60	126.32
64	113.96	119.78	127.58
65	109.42	116.89	124.59
66	107.03	116.89	125.17
67	108.45	116.63	118.76
68	104.53	112.71	116.87
69	100.01	106.68	112.69
70	98.01	104.99	112.25

Individual Animal Body Weight Gain (%)

Group: G5 (Recovery Vehicle: 0 mg/kg B.wt./day) Sex: Male

Animal Number	Days								
	8	15	22	29	36	43	50	57	64
81	1.65	5.78	12.35	21.64	31.09	50.40	56.71	58.67	66.39
82	1.26	7.13	13.22	22.31	32.20	47.56	54.78	61.58	69.02
83	1.75	6.21	12.89	22.42	31.53	46.49	51.14	56.94	71.80
84	0.67	6.84	11.27	19.89	32.37	48.42	54.56	59.54	72.08
85	0.81	7.47	12.19	19.50	33.33	49.59	54.16	58.14	74.59

Animal Number	Days					
	71	78	85	92	99	104
81	73.54	82.79	90.06	94.27	100.03	105.06
82	73.43	87.46	94.20	97.50	102.62	107.49
83	79.43	83.81	93.15	95.67	101.01	106.71
84	78.21	87.38	93.63	96.62	103.71	107.28
85	78.84	86.51	92.38	96.22	103.43	107.37

Group: G6 (Recovery High Dose: 41.2 mg/kg B.wt./day) Sex: Male

Animal Number	Days								
	8	15	22	29	36	43	50	57	64
91	1.68	9.23	17.33	25.17	32.77	51.23	56.29	64.29	76.53
92	1.92	9.32	16.64	22.50	33.30	51.66	58.30	62.09	73.80
93	1.40	6.87	15.41	18.41	31.89	49.37	53.85	63.65	73.37
94	1.24	4.58	9.91	20.32	31.04	46.06	52.84	59.34	74.40
95	1.31	5.80	9.68	20.16	30.64	49.16	52.92	57.03	69.53

Animal Number	Days					
	71	78	85	92	99	104
91	84.56	87.38	96.62	100.94	106.91	112.91
92	79.15	90.79	98.75	100.92	108.52	113.38
93	80.51	87.55	93.32	97.13	103.58	107.77
94	76.41	83.99	90.65	95.91	102.61	106.80
95	77.65	84.28	89.50	92.96	97.78	102.39

Individual Animal Body Weight Gain (%)

Group: G1 (Vehicle: 0 mg/kg B.wt./day) Sex: Female

Animal Number	Days									
	8	15	22	29	36	43	50	57	64	71
11	2.94	7.84	17.17	22.54	39.83	47.04	58.02	62.69	69.59	82.31
12	1.26	8.44	13.95	20.59	41.49	44.95	56.68	63.33	70.58	82.32
13	0.98	7.02	15.72	22.83	40.47	46.42	59.58	67.84	74.41	87.79
14	0.94	12.86	19.46	24.70	39.79	45.27	60.03	64.14	75.46	81.12
15	1.87	10.28	20.56	26.16	37.38	46.07	57.82	65.06	73.90	85.94
16	2.70	10.18	17.09	22.80	33.33	41.43	51.33	56.75	68.66	77.14
17	1.81	12.42	16.95	24.20	33.25	39.61	51.88	58.82	71.86	80.18
18	1.79	10.98	16.06	24.08	27.05	40.35	51.75	54.62	60.79	71.42
19	0.68	11.32	20.61	25.62	26.01	39.55	48.41	54.17	61.83	71.85
20	1.26	8.44	13.51	17.88	28.03	34.47	45.99	53.36	57.05	65.90

Animal Number	Days		
	78	85	90
11	88.84	97.04	105.86
12	89.79	95.27	102.54
13	96.49	104.03	109.80
14	92.44	104.26	112.46
15	94.37	104.23	109.91
16	89.15	95.87	100.88
17	89.03	99.30	104.66
18	85.01	96.40	102.62
19	82.55	93.22	101.87
20	82.07	88.28	95.58

Individual Animal Body Weight Gain (%)

Group: G2 (Low dose: 10.3 mg/kg B.wt./day) Sex: Female

Animal Number	Days									
	8	15	22	29	36	43	50	57	64	71
31	1.20	7.10	16.75	24.48	37.02	44.71	54.36	59.21	67.41	82.36
32	0.04	7.84	16.53	23.82	35.58	49.57	60.05	65.10	71.33	78.97
33	1.55	6.48	15.74	22.22	29.61	46.26	54.80	62.03	69.71	78.90
34	0.95	9.39	15.30	21.14	33.65	43.15	50.68	61.60	70.36	76.78
35	0.72	10.23	21.44	28.27	37.46	41.47	51.58	59.42	69.03	76.38
36	0.44	10.47	18.28	23.26	36.95	42.74	54.27	59.07	73.14	81.66
37	0.00	9.48	16.21	23.09	35.71	41.43	50.83	58.94	67.31	75.66
38	1.10	9.50	17.58	23.87	36.61	41.50	50.80	54.39	61.53	72.90
39	1.58	7.58	13.83	22.27	29.22	36.61	47.11	50.30	59.37	69.48
40	1.73	6.35	12.11	16.75	27.99	33.44	51.34	50.07	58.42	70.05

Animal Number	Days		
	78	85	90
31	92.84	99.29	109.05
32	89.35	99.08	108.18
33	94.40	103.56	109.82
34	91.12	97.78	109.93
35	90.55	100.76	110.94
36	88.61	97.42	105.42
37	88.06	97.06	104.49
38	88.47	94.18	105.22
39	84.94	91.56	96.60
40	83.88	90.33	95.88

Individual Animal Body Weight Gain (%)

Group: G3 (Mid Dose: 20.6 mg/kg B.wt./day) Sex: Female

Animal Number	Days									
	8	15	22	29	36	43	50	57	64	71
51	1.87	7.03	22.60	26.16	43.16	46.72	57.59	63.75	73.37	82.83
52	1.83	10.16	18.89	23.44	46.24	51.91	60.48	67.63	75.23	88.02
53	1.92	10.51	22.34	28.63	43.43	48.65	59.26	64.20	70.08	78.99
54	3.03	8.14	19.35	26.60	40.75	41.28	50.45	64.80	71.14	78.88
55	0.64	9.45	14.94	20.94	43.63	43.60	53.68	59.18	69.83	79.89
56	1.82	9.08	17.27	23.63	33.83	42.72	51.81	57.44	65.43	74.53
57	1.73	9.26	18.04	23.18	35.90	42.64	54.07	59.14	66.47	79.82
58	0.89	8.01	16.95	22.32	35.71	41.63	49.99	56.20	64.27	76.65
59	0.57	7.14	13.84	16.18	38.07	41.58	47.51	51.92	63.89	72.68
60	0.15	6.66	13.16	18.39	34.62	36.67	45.37	51.92	58.43	69.74

Animal Number	Days		
	78	85	90
51	96.22	102.45	110.66
52	100.01	106.86	115.20
53	95.70	103.49	114.74
54	93.23	100.68	110.87
55	96.69	101.35	106.19
56	90.87	98.75	106.01
57	88.50	97.98	107.54
58	87.46	94.50	101.77
59	89.26	92.87	96.72
60	90.16	94.57	98.95

Individual Animal Body Weight Gain (%)

Group: G4 (High Dose: 41.2 mg/kg B.wt./day) Sex: Female

Animal Number	Days									
	8	15	22	29	36	43	50	57	64	71
71	1.72	13.13	23.23	28.33	45.73	52.07	60.31	64.97	70.95	80.26
72	1.25	12.99	22.07	28.66	47.84	52.73	60.02	67.61	75.65	85.16
73	0.93	12.12	20.06	24.16	44.44	50.27	55.55	61.59	68.99	80.54
74	0.31	12.59	20.50	27.82	46.34	51.90	56.93	63.33	72.58	81.82
75	0.51	11.25	19.67	24.62	38.64	53.21	59.62	61.10	68.74	79.35
76	1.64	10.71	20.36	25.23	39.42	45.16	49.73	57.90	69.36	79.88
77	1.81	11.70	22.72	26.97	39.99	52.32	61.82	59.97	65.83	77.47
78	1.66	10.43	22.14	27.53	39.18	48.19	54.94	60.76	66.15	79.29
79	0.67	7.65	20.67	25.73	38.18	47.04	48.81	53.19	63.30	70.70
80	0.68	5.97	20.57	25.17	36.39	45.02	52.47	57.10	63.90	70.78

Animal Number	Days		
	78	85	90
71	86.94	97.72	104.98
72	97.58	103.90	113.29
73	96.47	102.32	111.09
74	93.05	102.40	108.79
75	98.40	100.33	103.57
76	98.94	104.38	108.19
77	90.89	98.75	104.19
78	92.66	96.65	103.86
79	91.39	95.47	100.75
80	80.04	89.50	93.80

Individual Animal Body Weight Gain (%)

Group: G5 (Recovery Vehicle: 0 mg/kg B.wt./day) Sex: Female

Animal Number	Days								
	8	15	22	29	36	43	50	57	64
86	1.48	6.38	13.76	18.66	29.30	37.48	43.73	48.93	55.18
87	1.28	6.98	12.72	19.49	28.23	36.59	45.38	49.98	51.92
88	1.44	6.48	12.95	17.39	25.10	33.58	44.88	51.47	55.67
89	0.81	7.17	12.19	20.74	24.39	35.77	42.27	46.23	51.64
90	0.89	6.00	12.01	18.05	24.44	33.84	44.52	48.56	56.59

Animal Number	Days					
	71	78	85	92	99	104
86	60.91	74.55	80.03	83.41	87.94	91.60
87	62.15	76.30	83.95	85.88	92.49	95.19
88	66.76	76.48	82.42	84.99	89.83	92.29
89	61.40	78.56	81.29	83.72	88.03	94.12
90	67.72	75.24	80.30	82.36	86.73	92.24

Group: G6 (Recovery High Dose: 41.2 mg/kg B.wt./day) Sex: Female

Animal Number	Days								
	8	15	22	29	36	43	50	57	64
96	2.12	7.50	15.00	21.66	30.86	36.85	45.83	49.97	54.99
97	1.64	6.25	13.91	20.95	26.75	37.70	44.26	48.51	54.88
98	0.03	5.38	12.23	18.89	29.55	34.46	43.84	48.10	52.50
99	1.05	4.92	12.09	19.32	27.39	35.45	44.35	48.38	55.25
100	0.53	5.50	10.91	18.62	26.44	33.27	39.82	43.51	43.83

Animal Number	Days					
	71	78	85	92	99	104
96	65.71	78.32	86.35	88.81	93.37	100.81
97	61.17	72.12	80.67	83.50	89.16	93.75
98	60.30	75.62	81.38	83.77	89.84	93.48
99	62.25	73.89	77.12	82.35	89.15	93.61
100	59.48	70.23	75.81	80.43	86.00	88.41

Individual Animal Food Consumption (g/day/rat)

Sex: Male

Group	Animal Number	Cage Number	Weeks 1	2	3	4	5	6	7	8
G1 (Vehicle: 0 mg/kg B.wt./day)	1	1	19.10	19.07	19.14	19.09	19.14	19.09	19.16	18.91
	2									
	3									
	4	2	28.38	28.51	28.37	28.51	28.53	28.61	28.63	28.53
	5									
	6	3	19.14	19.10	19.07	19.09	19.15	19.15	19.14	18.89
	7									
	8									
	9	4	28.56	28.51	28.52	28.59	28.50	28.62	28.65	28.71
	10									
G2 (Low Dose: 10.3 mg/kg B.wt./day)	21	5	19.20	19.15	19.16	19.14	19.13	19.15	19.18	18.91
	22									
	23									
	24	6	28.64	28.52	28.57	28.62	28.56	28.65	28.66	28.13
	25									
	26	7	19.14	19.09	19.10	19.12	19.11	19.19	19.11	19.11
	27									
	28									
	29	8	28.50	28.58	28.59	28.59	28.59	28.67	28.63	28.34
	30									

Individual Animal Food Consumption (g/day/rat)

Sex: Male

Group	Animal Number	Cage Number	Weeks							
			1	2	3	4	5	6	7	8
G3 (Mid Dose: 20.6 mg/kg B.wt./day)	41	9	19.10	19.10	19.16	19.14	19.15	19.20	19.15	18.84
	42									
	43									
	44	10	28.59	28.57	28.58	28.52	28.37	28.71	28.55	28.09
	45									
	46	11	19.08	19.15	19.10	19.09	19.08	19.12	19.18	18.77
	47									
	48									
	49	12	28.57	28.58	28.58	28.61	28.55	28.77	28.68	28.37
	50									
G4 (High Dose: 41.2 mg/kg B.wt./day)	61	13	19.30	19.24	19.24	19.10	19.10	19.10	19.20	18.70
	62									
	63									
	64	14	28.35	28.49	28.52	28.52	28.55	28.71	28.65	27.09
	65									
	66	15	19.25	19.22	19.17	19.10	19.11	19.10	19.18	18.62
	67									
	68									
	69	16	28.48	28.61	28.59	28.56	28.59	28.63	28.62	27.82
	70									

Individual Animal Food Consumption (g/day/rat)

Sex: Male

Group	Animal Number	Cage Number	Weeks							
			1	2	3	4	5	6	7	8
G5 (Recovery Vehicle: 0 mg/kg B.wt./day)	81	17	19.11	19.13	19.13	19.11	19.09	19.13	19.22	19.11
	82									
	83									
	84	18	28.65	28.62	28.60	28.58	28.59	28.67	28.63	28.29
	85									
G6 (Recovery High Dose: 41.2 mg/kg B.wt./day)	91	19	19.08	19.07	19.11	19.11	19.10	19.14	19.24	18.90
	92									
	93									
	94	20	28.59	28.65	28.68	28.73	28.67	28.70	28.73	28.19
	95									

Individual Animal Food Consumption (g/day/rat)

Sex: Male

Group	Animal Number	Cage Number	Weeks				
			9	10	11	12	13
G1 (Vehicle: 0 mg/kg B.wt./day)	1	1	11.72	19.02	11.93	21.43	16.73
	2						
	3						
	4	2	17.25	27.71	17.93	31.98	24.59
	5						
	6	3	11.90	18.89	11.95	21.38	16.68
	7						
	8						
	9	4	17.42	27.97	17.43	31.79	24.40
	10						
G2 (Low Dose: 10.3 mg/kg B.wt./day)	21	5	12.67	19.07	12.24	21.47	16.61
	22						
	23						
	24	6	18.63	27.82	18.05	31.97	24.61
	25						
	26	7	11.87	18.91	12.34	21.52	16.69
	27						
	28						
	29	8	17.51	28.18	18.36	31.85	24.50
	30						

Individual Animal Food Consumption (g/day/rat)

Sex: Male

<table>
<tr><th rowspan="2">Group</th><th rowspan="2">Animal Number</th><th rowspan="2">Cage Number</th><th colspan="5">Weeks</th></tr>
<tr><th>9</th><th>10</th><th>11</th><th>12</th><th>13</th></tr>
<tr><td rowspan="10">G3 (Mid Dose: 20.6 mg/kg B.wt./day)</td><td>41</td><td rowspan="3">9</td><td rowspan="3">11.90</td><td rowspan="3">18.75</td><td rowspan="3">12.31</td><td rowspan="3">21.39</td><td rowspan="3">16.21</td></tr>
<tr><td>42</td></tr>
<tr><td>43</td></tr>
<tr><td>44</td><td rowspan="2">10</td><td rowspan="2">17.34</td><td rowspan="2">27.96</td><td rowspan="2">18.21</td><td rowspan="2">31.76</td><td rowspan="2">24.15</td></tr>
<tr><td>45</td></tr>
<tr><td>46</td><td rowspan="3">11</td><td rowspan="3">11.23</td><td rowspan="3">19.40</td><td rowspan="3">11.94</td><td rowspan="3">21.24</td><td rowspan="3">16.75</td></tr>
<tr><td>47</td></tr>
<tr><td>48</td></tr>
<tr><td>49</td><td rowspan="2">12</td><td rowspan="2">17.21</td><td rowspan="2">28.25</td><td rowspan="2">17.61</td><td rowspan="2">31.73</td><td rowspan="2">24.48</td></tr>
<tr><td>50</td></tr>
<tr><td rowspan="10">G4 (High Dose: 41.2 mg/kg B.wt./day)</td><td>61</td><td rowspan="3">13</td><td rowspan="3">11.58</td><td rowspan="3">19.14</td><td rowspan="3">11.87</td><td rowspan="3">21.50</td><td rowspan="3">16.66</td></tr>
<tr><td>62</td></tr>
<tr><td>63</td></tr>
<tr><td>64</td><td rowspan="2">14</td><td rowspan="2">17.19</td><td rowspan="2">27.92</td><td rowspan="2">17.37</td><td rowspan="2">31.92</td><td rowspan="2">24.37</td></tr>
<tr><td>65</td></tr>
<tr><td>66</td><td rowspan="3">15</td><td rowspan="3">11.86</td><td rowspan="3">19.01</td><td rowspan="3">11.92</td><td rowspan="3">21.28</td><td rowspan="3">16.68</td></tr>
<tr><td>67</td></tr>
<tr><td>68</td></tr>
<tr><td>69</td><td rowspan="2">16</td><td rowspan="2">17.43</td><td rowspan="2">28.10</td><td rowspan="2">17.52</td><td rowspan="2">31.73</td><td rowspan="2">25.01</td></tr>
<tr><td>70</td></tr>
</table>

Individual Animal Food Consumption (g/day/rat)

Sex: Male

Group	Animal Number	Cage Number	Weeks						
			9	10	11	12	13	14	15
G5 (Recovery Vehicle: 0 mg/kg B.wt./day)	81	17	11.88	18.94	11.77	21.29	12.35	12.43	17.35
	82								
	83								
	84	18	17.32	27.93	17.60	31.72	17.60	18.07	24.90
	85								
G6 (Recovery High Dose: 41.2 mg/kg B.wt./day)	91	19	11.50	19.01	11.85	21.42	12.19	12.45	17.32
	92								
	93								
	94	20	17.16	28.10	17.58	31.77	17.64	17.90	24.91
	95								

Individual Animal Food Consumption (g/day/rat)

Sex: Female

Group	Animal Number	Cage Number	Weeks							
			1	2	3	4	5	6	7	8
G1 (Vehicle: 0 mg/kg B.wt./day)	11	21	19.12	19.07	19.05	19.04	19.05	19.07	19.06	19.08
	12									
	13									
	14	22	28.20	28.17	28.11	28.22	28.16	28.60	28.39	28.73
	15									
	16	23	18.99	18.92	18.92	18.92	18.90	18.96	18.88	19.03
	17									
	18									
	19	24	28.15	28.08	28.33	28.30	28.40	28.52	27.95	28.65
	20									
G2 (Low Dose: 10.3 mg/kg B.wt./day)	31	25	19.07	19.01	18.82	18.91	18.89	18.84	18.95	18.99
	32									
	33									
	34	26	28.23	28.14	28.15	28.18	28.17	28.29	28.08	28.37
	35									
	36	27	19.01	18.99	19.04	18.91	18.86	18.94	19.01	19.25
	37									
	38									
	39	28	28.56	28.56	28.50	28.25	28.34	28.60	27.86	28.62
	40									

Individual Animal Food Consumption (g/day/rat)

Sex: Female

Group	Animal Number	Cage Number	Weeks							
			1	2	3	4	5	6	7	8
G3 (Mid Dose: 20.6 mg/kg B.wt./day)	51	29	19.05	19.00	18.86	18.84	19.04	19.14	19.09	19.01
	52									
	53									
	54	30	28.57	28.58	28.34	28.30	28.47	28.54	28.41	28.28
	55									
	56	31	19.00	19.00	19.06	19.04	19.04	19.05	18.91	19.19
	57									
	58									
	59	32	28.21	28.27	28.61	28.55	28.57	28.70	27.68	28.15
	60									
G4 (High Dose: 41.2 mg/kg B.wt./day)	71	33	19.01	18.91	19.02	18.91	18.92	19.01	19.02	19.00
	72									
	73									
	74	34	28.14	28.34	28.36	28.22	28.50	28.54	28.35	28.35
	75									
	76	35	19.06	19.01	18.84	18.73	18.84	18.96	18.92	19.11
	77									
	78									
	79	36	28.09	28.23	28.20	27.86	27.92	28.49	28.23	28.35
	80									

Individual Animal Food Consumption (g/day/rat)

Sex: Female

Group	Animal Number	Cage Number	Weeks							
			1	2	3	4	5	6	7	8
G5 (Recovery Vehicle: 0 mg/kg B.wt./day)	86	37	19.06	19.02	18.78	18.73	19.01	19.03	19.02	19.04
	87									
	88									
	89	38	28.48	28.33	28.22	27.95	27.90	28.62	28.39	28.05
	90									
G6 (Recovery High Dose: 41.2 mg/kg B.wt./day)	96	39	19.05	18.80	18.74	19.00	19.03	18.95	19.09	18.94
	97									
	98									
	99	40	28.48	28.33	27.95	28.36	27.94	28.43	28.23	27.97
	100									

Individual Animal Food Consumption (g/day/rat)

Sex: Female

Group	Animal Number	Cage Number	Weeks				
			9	10	11	12	13
G1 (Vehicle: 0 mg/kg B.wt./day)	11	21	11.72	21.15	11.96	21.31	16.70
	12						
	13						
	14	22	17.20	31.51	17.56	31.87	24.86
	15						
	16	23	11.64	21.03	11.85	21.40	16.77
	17						
	18						
	19	24	17.07	31.21	17.43	31.79	24.99
	20						
G2 (Low Dose: 10.3 mg/kg B.wt./day)	31	25	11.71	20.99	11.89	21.29	16.88
	32						
	33						
	34	26	17.07	31.15	17.45	31.77	24.61
	35						
	36	27	11.68	20.92	11.76	21.41	16.77
	37						
	38						
	39	28	17.02	31.26	17.33	31.67	24.69
	40						

Individual Animal Food Consumption (g/day/rat)

Sex: Female

Group	Animal Number	Cage Number	Weeks 9	10	11	12	13
G3 (Mid Dose: 20.6 mg/kg B.wt./day)	51	29	11.46	21.04	11.64	21.50	16.40
	52						
	53						
	54	30	17.07	31.15	17.22	32.11	24.26
	55						
	56	31	9.48	21.02	11.93	21.54	16.71
	57						
	58						
	59	32	13.97	31.28	17.46	31.92	24.71
	60						
G4 (High Dose: 41.2 mg/kg B.wt./day)	71	33	9.73	20.92	11.79	21.49	16.80
	72						
	73						
	74	34	15.10	31.06	17.38	32.00	24.86
	75						
	76	35	9.97	20.90	11.50	21.27	16.73
	77						
	78						
	79	36	16.15	31.14	17.09	31.66	24.61
	80						

Individual Animal Food Consumption (g/day/rat)

Sex: Female

Group	Animal Number	Cage Number	Weeks						
			9	10	11	12	13	14	15
G5 (Recovery Vehicle: 0 mg/kg B.wt./day)	86	37	10.50	20.83	11.55	21.25	12.39	0.07	17.33
	87								
	88								
	89	38	15.93	31.15	17.19	31.75	17.65	0.16	25.55
	90								
G6 (Recovery High Dose: 41.2 mg/kg B.wt./day)	96	39	10.29	20.93	11.77	21.31	12.34	0.29	17.11
	97								
	98								
	99	40	15.05	31.02	17.55	31.68	17.21	0.00	25.07
	100								
