# Supplementary material for: 90-day oral toxicity study of a salmon nasal cartilage extract containing undenatured collagen and proteoglycan in Sprague-Dawley rats
Source: PLoS One. 2026 Jan 23;21(1):e0340675. doi: 10.1371/journal.pone.0340675 (PMC12829970; doi:10.1371/journal.pone.0340675)
Supplement: S4 Data set — (PDF) [file pone.0340675.s004.pdf]

Individual Animal Hematology Parameters

Group: G1 (Vehicle: 0 mg/kg B.wt./day) Sex: Male

Animal Number	Parameters							
	RBC (10^{12}/L)	WBC (10^9/L)	HGB (g/dL)	HCT (%)	PLT (10^3/uL)	Neu** (%)	Lymp** (%)	Eosi** (%)
1	10.79	13.61	15.6	50.7	890	5.17	71.74	0.34
2	10.42	13.49	16.7	50.3	745	1.51	77.76	2.82
3	10.92	12.73	16.5	52.9	862	8.20	64.37	3.54
4	10.22	5.84	14.2	46.7	921	18.68	64.13	3.64
5	9.55	7.84	14.6	47.1	1291	19.86	56.43	8.26
6	10.48	5.73	15.8	52.8	601	12.06	70.83	2.05
7	11.39	9.43	14.9	51.8	748	16.77	69.67	2.57
8	9.53	5.70	14.1	47.6	1137	20.63	61.54	1.35
9	10.83	7.16	15.9	53.6	1012	18.84	67.03	2.05
10	10.42	6.15	14.1	47.7	945	15.52	60.65	1.66

Animal Number	Parameters							
	Mono** (%)	Baso** (%)	Neu* (10^9/L)	Lymp* (10^9/L)	Eosi* (10^9/L)	Mono* (10^9/L)	Baso* (10^9/L)	Retic (%)
1	22.71	0.04	0.706	9.763	0.046	3.090	0.005	15.5
2	17.91	0.00	0.205	10.489	0.380	2.416	0.000	15.3
3	23.77	0.12	1.046	8.194	0.450	3.025	0.015	15.7
4	13.37	0.18	1.093	3.745	0.212	0.780	0.010	17.1
5	15.35	0.10	1.55	4.424	0.647	1.203	0.007	17.1
6	14.84	0.22	0.693	4.058	0.117	0.850	0.012	14.7
7	10.89	0.10	1.584	6.569	0.242	1.026	0.009	15.1
8	16.35	0.13	1.179	3.507	0.076	0.931	0.007	13.7
9	11.92	0.16	1.351	4.799	0.146	0.853	0.011	13.4
10	21.87	0.30	0.956	3.729	0.102	1.345	0.018	13.0

Note: * Represents absolute Leucocytes Counts, ** Represents Relative Leucocytes Counts.

Individual Animal Hematology Parameters

Group: G2 (Low Dose: 10.3 mg/kg B.wt./day) Sex: Male

Animal Number	Parameters							
	RBC (10^{12}/L)	WBC (10^{9}/L)	HGB (g/dL)	HCT (%)	PLT (10^{3}/uL)	Neu** (%)	Lymp** (%)	Eosi** (%)
21	9.66	7.89	14.8	47.6	1375	6.50	59.54	0.69
22	10.45	9.66	15.4	52.1	844	12.55	67.91	4.56
23	9.81	3.96	14.5	48.4	868	24.77	53.53	5.14
24	11.80	10.37	16.4	55.6	1102	11.01	66.99	4.30
25	8.85	1.49	13.7	42.3	1046	18.37	62.89	12.36
26	11.01	9.79	16.0	52.5	992	15.56	63.56	7.94
27	9.66	9.43	14.7	48.5	810	15.59	58.76	7.12
28	10.02	8.73	15.6	52.1	986	15.17	65.64	6.87
29	11.16	14.90	16.1	52.7	710	13.88	70.32	4.60
30	10.55	9.64	15.6	52.0	863	21.61	60.19	4.24

Animal Number	Parameters							
	Mono** (%)	Baso** (%)	Neu* (10^{9}/L)	Lymp* (10^{9}/L)	Eosi* (10^{9}/L)	Mono* (10^{9}/L)	Baso* (10^{9}/L)	Retic (%)
21	33.25	0.02	0.515	4.697	0.054	2.623	0.001	0.0
22	14.46	0.52	1.214	6.560	0.440	1.396	0.050	0.0
23	16.29	0.27	0.983	2.119	0.203	0.645	0.010	12.2
24	17.54	0.16	1.145	6.946	0.445	1.818	0.016	11.7
25	6.39	0.00	0.274	0.937	0.184	0.095	0.000	11.3
26	12.42	0.52	1.526	6.222	0.777	1.215	0.050	14.6
27	18.11	0.42	1.472	5.541	0.671	1.707	0.039	9.8
28	11.92	0.40	1.327	5.730	0.599	1.040	0.034	8.9
29	10.98	0.22	2.070	10.477	0.685	1.636	0.032	8.2
30	13.56	0.40	2.085	5.802	0.408	1.307	0.038	7.7

Note: * Represents absolute Leucocytes Counts, ** Represents Relative Leucocytes Counts.

Individual Animal Hematology Parameters

Group: G3 (Mid Dose: 20.6 mg/kg B.wt./day) Sex: Male

Animal Number	Parameters							
	RBC (10^{12}/L)	WBC (10^9/L)	HGB (g/dL)	HCT (%)	PLT (10^3/uL)	Neu** (%)	Lymp** (%)	Eosi** (%)
41	10.17	11.47	15.1	50.3	706	16.82	63.18	8.03
42	9.16	6.61	13.5	43.9	686	8.95	59.51	1.47
43	9.80	9.04	13.4	50.9	993	15.31	60.18	7.94
44	9.69	5.98	15.1	48.9	982	13.01	47.62	10.43
45	11.06	9.63	16.1	54.3	1236	15.34	61.14	6.11
46	10.56	6.49	15.7	52.6	894	21.42	56.04	8.02
47	10.14	8.80	15.9	52.5	831	16.32	58.61	7.24
48	10.50	6.24	15.9	51.7	784	8.32	67.01	3.38
49	10.43	17.37	16.0	53.1	931	6.49	76.04	0.71
50	10.89	12.60	13.1	51.0	934	6.98	72.95	0.65

Animal Number	Parameters							
	Mono** (%)	Baso** (%)	Neu* (10^9/L)	Lymp* (10^9/L)	Eosi* (10^9/L)	Mono* (10^9/L)	Baso* (10^9/L)	Retic (%)
41	11.71	0.26	1.931	7.246	0.921	1.343	0.029	8.0
42	30.01	0.06	0.594	3.933	0.097	1.983	0.003	7.9
43	15.99	0.58	1.386	5.440	0.717	1.445	0.052	8.0
44	28.76	0.18	0.781	2.847	0.623	1.719	0.010	6.6
45	17.31	0.10	1.480	5.887	0.588	1.666	0.009	7.3
46	14.44	0.08	1.392	3.636	0.520	0.937	0.005	8.6
47	17.60	0.23	1.438	5.157	0.637	1.548	0.020	8.4
48	21.27	0.02	0.521	4.181	0.210	1.327	0.001	6.3
49	16.72	0.04	1.129	13.208	0.123	2.904	0.006	8.3
50	19.34	0.08	0.882	9.191	0.081	2.436	0.010	7.2

Note: * Represents absolute Leucocytes Counts, ** Represents Relative Leucocytes Counts.

Individual Animal Hematology Parameters

Group: G4 (High Dose: 41.2 mg/kg B.wt./day) Sex: Male

Animal Number	Parameters							
	RBC (10^{12}/L)	WBC (10^9/L)	HGB (g/dL)	HCT (%)	PLT (10^3/uL)	Neu** (%)	Lymp** (%)	Eosi** (%)
61	11.08	10.49	15.3	51.6	994	32.48	45.62	7.60
62	11.16	12.83	16.1	54.2	962	17.65	63.48	6.22
63	9.81	17.00	14.8	46.8	894	12.65	68.15	5.49
64	10.15	7.59	15.4	50.5	584	11.06	69.36	7.73
65	10.11	9.20	15.7	51.0	1158	11.96	65.87	7.88
66	10.48	3.84	15.2	50.7	760	17.56	53.61	10.33
67	10.46	2.81	14.8	49.3	1094	13.71	52.18	2.72
68	10.09	14.08	16.4	52.1	1071	18.50	19.96	46.74
69	10.84	10.21	15.7	52.4	1090	21.16	41.27	26.36
70	10.08	11.32	15.0	50.6	1047	8.33	35.58	35.56

Animal Number	Parameters							
	Mono** (%)	Baso** (%)	Neu* (10^9/L)	Lymp* (10^9/L)	Eosi* (10^9/L)	Mono* (10^9/L)	Baso* (10^9/L)	Retic (%)
61	13.80	0.50	3.409	4.785	0.797	1.447	0.052	8.3
62	12.59	0.06	2.266	8.144	0.798	1.615	0.007	7.5
63	13.57	0.14	2.153	11.585	0.933	2.306	0.023	7.7
64	11.77	0.08	0.841	5.264	0.586	0.893	0.006	6.1
65	14.15	0.14	1.103	6.060	0.724	1.301	0.012	7.8
66	18.25	0.25	0.677	2.058	0.396	0.700	0.009	6.5
67	31.33	0.06	0.387	1.466	0.076	0.880	0.001	6.6
68	14.48	0.32	2.607	2.810	6.580	2.038	0.045	6.8
69	11.13	0.08	2.162	4.213	2.691	1.136	0.008	6.8
70	19.77	0.76	0.945	4.027	4.025	2.237	0.086	6.0

Note: * Represents absolute Leucocytes Counts, ** Represents Relative Leucocytes Counts.

Individual Animal Hematology Parameters

Group: G5 (Recovery Vehicle: 0 mg/kg B.wt./day) Sex: Male

Animal Number	Parameters							
	RBC (10^{12}/L)	WBC (10^9/L)	HGB (g/dL)	HCT (%)	PLT (10^3/uL)	Neu** (%)	Lymp** (%)	Eosi** (%)
81	10.92	5.06	17.4	54.9	903	18.72	59.22	6.57
82	10.93	4.47	16.7	55.3	921	23.72	58.34	5.38
83	10.71	5.63	16.4	54.1	957	22.52	58.44	4.31
84	9.91	5.54	16.2	52.0	1147	34.86	45.79	7.36
85	10.48	5.47	15.9	52.5	482	19.67	58.62	6.32

Animal Number	Parameters							
	Mono** (%)	Baso** (%)	Neu* (10^9/L)	Lymp* (10^9/L)	Eosi* (10^9/L)	Mono* (10^9/L)	Baso* (10^9/L)	Retic (%)
81	15.45	0.04	0.949	2.996	0.332	0.781	0.002	7.1
82	12.41	0.15	1.063	2.607	0.240	0.554	0.006	12.7
83	14.59	0.14	1.270	3.290	0.242	0.821	0.007	10.5
84	11.74	0.25	1.934	2.536	0.407	0.650	0.013	8.1
85	15.21	0.18	1.079	3.206	0.345	0.831	0.009	7.9

Group: G6 (Recovery High Dose: 41.2 mg/kg B.wt./day) Sex: Male

Animal Number	Parameters							
	RBC (10^{12}/L)	WBC (10^9/L)	HGB (g/dL)	HCT (%)	PLT (10^3/uL)	Neu** (%)	Lymp** (%)	Eosi** (%)
91	10.73	8.48	15.6	49.4	708	11.56	64.32	4.51
92	11.35	3.06	17.9	59.1	1014	20.61	52.53	6.16
93	11.35	11.93	16.2	52.4	826	9.55	62.10	6.31
94	10.59	6.19	15.4	51.8	938	15.93	61.96	7.16
95	10.88	9.09	16.1	54.2	868	13.43	62.22	8.58

Animal Number	Parameters							
	Mono** (%)	Baso** (%)	Neu* (10^9/L)	Lymp* (10^9/L)	Eosi* (10^9/L)	Mono* (10^9/L)	Baso* (10^9/L)	Retic (%)
91	19.55	0.06	0.982	5.454	0.382	1.657	0.005	8.1
92	20.58	0.12	0.633	1.607	0.188	0.629	0.003	7.1
93	21.94	0.10	1.142	7.408	0.752	2.617	0.011	8.1
94	14.67	0.28	0.987	3.835	0.443	0.908	0.017	7.0
95	15.39	0.38	1.224	5.655	0.779	1.398	0.034	7.0

Note: * Represents absolute Leucocytes Counts, ** Represents Relative Leucocytes Counts.

Individual Animal Hematology Parameters

Group: G1 (Vehicle: 0 mg/kg B.wt./day) Sex: Female

Animal Number	Parameters							
	RBC (10^{12}/L)	WBC (10^9/L)	HGB (g/dL)	HCT (%)	PLT (10^3/uL)	Neu** (%)	Lymp** (%)	Eosi** (%)
11	7.83	8.59	13.3	37.3	448	9.65	47.76	29.24
12	7.65	7.44	13.0	40.2	975	12.89	41.15	33.59
13	9.41	3.89	14.2	46.0	1138	17.84	40.54	29.22
14	9.94	6.83	15.8	51.4	1442	15.62	50.65	17.10
15	8.38	4.96	12.6	42.2	289	22.62	48.28	18.87
16	9.54	5.40	14.7	48.5	1266	20.97	54.54	11.56
17	9.05	11.31	13.2	43.4	853	10.22	59.73	14.04
18	9.70	5.26	15.8	51.3	944	19.29	44.30	17.41
19	7.85	6.98	11.9	37.3	571	9.63	60.84	12.58
20	9.55	5.25	14.9	49.1	1122	11.32	48.63	10.94

Animal Number	Parameters							
	Mono** (%)	Baso** (%)	Neu* (10^9/L)	Lymp* (10^9/L)	Eosi* (10^9/L)	Mono* (10^9/L)	Baso* (10^9/L)	Retic (%)
11	13.00	0.35	0.831	4.102	2.511	1.116	0.030	6.0
12	12.10	0.27	0.96	3.061	2.499	0.900	0.020	5.8
13	12.20	0.20	0.696	1.577	1.136	0.474	0.007	6.8
14	16.15	0.48	1.069	3.459	1.167	1.103	0.032	5.7
15	9.91	0.32	1.125	2.394	0.935	0.491	0.015	5.8
16	12.68	0.25	1.134	2.945	0.624	0.684	0.013	6.2
17	15.69	0.32	1.158	6.755	1.587	1.774	0.036	5.9
18	18.71	0.29	1.016	2.330	0.915	0.984	0.015	5.3
19	16.61	0.34	0.674	4.246	0.878	1.159	0.023	8.7
20	28.84	0.27	0.595	2.553	0.574	1.514	0.014	6.8

Note: * Represents absolute Leucocytes Counts, ** Represents Relative Leucocytes Counts.

Individual Animal Hematology Parameters

Group: G2 (Low Dose: 10.3 mg/kg B.wt./day) Sex: Female

Animal Number	Parameters							
	RBC (10^{12}/L)	WBC (10^9/L)	HGB (g/dL)	HCT (%)	PLT (10^3/uL)	Neu** (%)	Lymp** (%)	Eosi** (%)
31	7.52	6.34	13.4	39.4	1010	11.12	58.15	15.52
32	9.69	3.34	14.7	47.3	1223	17.20	49.80	18.30
33	9.97	6.98	16.0	51.8	1508	16.44	56.77	13.44
34	8.92	4.12	14.3	45.2	425	21.10	50.52	18.10
35	9.44	5.93	15.2	48.0	1277	20.37	55.32	12.16
36	9.13	11.37	13.6	44.0	860	10.31	64.17	9.57
37	9.59	5.26	15.7	50.7	987	17.16	47.52	19.30
38	10.17	4.75	16.5	52.4	1081	22.91	53.96	11.13
39	10.02	9.71	14.3	46.8	917	33.71	49.11	6.95
40	5.66	7.49	9.1	26.6	568	15.33	22.02	14.60

Animal Number	Parameters							
	Mono** (%)	Baso** (%)	Neu* (10^9/L)	Lymp* (10^9/L)	Eosi* (10^9/L)	Mono* (10^9/L)	Baso* (10^9/L)	Retic (%)
31	15.07	0.14	0.708	3.686	0.983	0.955	0.008	6.7
32	14.20	0.50	0.576	1.663	0.611	0.474	0.016	6.6
33	13.01	0.34	1.149	3.962	0.938	0.908	0.023	7.0
34	9.79	0.49	0.871	2.081	0.745	0.403	0.020	6.0
35	11.75	0.40	1.210	3.280	0.721	0.696	0.023	6.7
36	15.71	0.24	1.173	7.296	1.088	1.786	0.027	5.7
37	15.77	0.25	0.904	2.499	1.015	0.829	0.013	5.7
38	11.39	0.61	1.090	2.563	0.528	0.541	0.028	5.4
39	10.09	0.14	3.276	4.768	0.674	0.979	0.013	6.0
40	47.83	0.22	1.150	1.649	1.093	3.582	0.016	5.9

Note: * Represents absolute Leucocytes Counts, ** Represents Relative Leucocytes Counts.

Individual Animal Hematology Parameters

Group: G3 (Mid Dose: 20.6 mg/kg B.wt./day) Sex: Female

Animal Number	Parameters							
	RBC (10^{12}/L)	WBC (10^9/L)	HGB (g/dL)	HCT (%)	PLT (10^3/uL)	Neu** (%)	Lymp** (%)	Eosi** (%)
51	7.58	6.86	13.2	39.9	1030	10.87	59.96	12.61
52	9.76	11.87	15.1	49.1	992	9.45	70.52	1.89
53	8.93	7.72	14.6	46.7	746	17.98	60.46	5.26
54	10.28	12.25	15.7	50.8	675	18.38	60.35	5.69
55	9.05	4.45	14.9	46.8	980	19.75	53.29	9.19
56	9.43	3.19	14.4	46.0	1255	19.72	55.75	9.51
57	9.71	7.44	15.9	50.4	1508	15.98	61.58	9.86
58	6.12	1.17	10.2	31.4	1519	27.75	47.28	17.87
59	9.19	5.51	15.4	47.4	1436	20.50	61.73	5.38
60	8.95	11.64	13.6	43.2	967	5.97	74.86	1.55

Animal Number	Parameters							
	Mono** (%)	Baso** (%)	Neu* (10^9/L)	Lymp* (10^9/L)	Eosi* (10^9/L)	Mono* (10^9/L)	Baso* (10^9/L)	Retic (%)
51	16.36	0.20	0.747	4.113	0.865	1.122	0.013	5.8
52	17.92	0.22	1.123	8.370	0.224	2.127	0.026	5.5
53	16.22	0.08	1.389	4.667	0.406	1.252	0.006	5.4
54	15.48	0.10	2.253	7.392	0.697	1.896	0.012	5.7
55	17.31	0.46	0.881	2.371	0.408	0.770	0.020	5.8
56	14.59	0.43	0.631	1.778	0.303	0.465	0.013	5.6
57	12.17	0.41	1.191	4.581	0.733	0.905	0.030	5.4
58	7.10	0.00	0.325	0.553	0.209	0.083	0.000	6.3
59	11.95	0.44	1.131	3.401	0.296	0.658	0.024	5.4
60	17.60	0.02	0.697	8.713	0.180	2.048	0.002	5.8

Note: * Represents absolute Leucocytes Counts, ** Represents Relative Leucocytes Counts.

Individual Animal Hematology Parameters

Group: G4 (High Dose: 41.2 mg/kg B.wt./day) Sex: Female

Animal Number	Parameters							
	RBC (10^{12}/L)	WBC (10^9/L)	HGB (g/dL)	HCT (%)	PLT (10^3/uL)	Neu** (%)	Lymp** (%)	Eosi** (%)
71	9.35	4.92	16.1	49.8	983	1.87	61.46	6.82
72	9.60	4.76	15.5	49.5	1162	21.94	57.42	8.26
73	10.15	10.13	14.7	47.4	1006	31.12	51.59	5.02
74	7.05	0.79	11.3	33.9	653	12.02	47.89	26.98
75	7.24	6.33	13.0	38.4	1025	6.96	63.90	9.24
76	9.86	11.36	15.5	49.8	1060	7.40	74.52	0.97
77	8.92	7.82	14.7	46.2	865	19.92	60.35	3.57
78	10.50	12.86	15.3	51.8	435	17.71	60.00	6.37
79	9.23	4.47	14.8	47.6	1074	16.39	57.67	6.28
80	9.40	3.19	14.5	45.7	1279	13.61	65.79	4.07

Animal Number	Parameters							
	Mono** (%)	Baso** (%)	Neu* (10^9/L)	Lymp* (10^9/L)	Eosi* (10^9/L)	Mono* (10^9/L)	Baso* (10^9/L)	Retic (%)
71	29.81	0.04	0.095	3.023	0.335	1.466	0.001	5.6
72	11.98	0.40	1.045	2.733	0.393	0.570	0.019	5.6
73	12.11	0.16	3.154	5.226	0.508	1.226	0.016	6.2
74	13.11	0.00	0.096	0.378	0.213	0.103	0.000	4.8
75	19.74	0.16	0.443	4.044	0.584	1.249	0.010	5.3
76	17.07	0.04	0.842	8.465	0.110	1.939	0.004	6.0
77	15.93	0.23	1.260	4.719	0.279	1.245	0.017	5.7
78	15.74	0.18	2.278	7.716	0.819	2.024	0.023	6.1
79	19.51	0.15	0.735	2.577	0.280	0.872	0.006	5.7
80	16.47	0.06	0.437	2.098	0.129	0.525	0.001	5.3

Note: * Represents absolute Leucocytes Counts, ** Represents Relative Leucocytes Counts.

Individual Animal Hematology Parameters

Group: G5 (Recovery Vehicle: 0 mg/kg B.wt./day) Sex: Female

Animal Number	Parameters							
	RBC (10^{12}/L)	WBC (10^9/L)	HGB (g/dL)	HCT (%)	PLT (10^3/uL)	Neu** (%)	Lymp** (%)	Eosi** (%)
86	9.75	4.41	15.8	49.2	1084	20.86	57.07	10.41
87	7.57	1.79	12.1	39.1	657	17.79	55.63	13.02
88	10.35	8.08	16.6	52.9	1048	19.72	58.27	2.11
89	9.50	7.98	15.0	48.6	940	13.58	67.10	1.22
90	10.36	5.40	15.7	52.6	1141	20.41	53.20	13.67

Animal Number	Parameters							
	Mono** (%)	Baso** (%)	Neu* (10^9/L)	Lymp* (10^9/L)	Eosi* (10^9/L)	Mono* (10^9/L)	Baso* (10^9/L)	Retic (%)
86	11.26	0.40	0.922	2.516	0.459	0.496	0.017	7.8
87	13.35	0.21	0.321	0.995	0.233	0.238	0.003	7.2
88	19.72	0.18	1.595	4.708	0.170	1.593	0.014	7.8
89	17.91	0.19	1.085	5.354	0.097	1.429	0.015	7.2
90	12.54	0.18	1.104	2.872	0.738	0.677	0.009	7.5

Group: G6 (Recovery High Dose: 41.2 mg/kg B.wt./day) Sex: Male

Animal Number	Parameters							
	RBC (10^{12}/L)	WBC (10^9/L)	HGB (g/dL)	HCT (%)	PLT (10^3/uL)	Neu** (%)	Lymp** (%)	Eosi** (%)
96	5.72	0.59	9.3	29.0	607	31.56	46.07	7.02
97	10.60	9.59	16.9	54.4	1138	22.68	60.90	1.81
98	9.66	7.91	15.4	49.4	966	14.78	67.45	1.53
99	8.85	3.79	15.2	46.9	829	16.86	55.82	8.50
100	9.12	4.72	15.2	48.7	836	15.60	58.02	5.52

Animal Number	Parameters							
	Mono** (%)	Baso** (%)	Neu* (10^9/L)	Lymp* (10^9/L)	Eosi* (10^9/L)	Mono* (10^9/L)	Baso* (10^9/L)	Retic (%)
96	15.35	0.00	0.188	0.271	0.041	0.090	0.000	7.0
97	14.39	0.22	2.176	5.840	0.173	1.380	0.021	7.0
98	16.20	0.04	1.170	5.335	0.121	1.281	0.003	6.4
99	18.49	0.33	0.641	2.115	0.322	0.700	0.012	6.9
100	20.61	0.25	0.739	2.738	0.260	0.972	0.011	7.3

Note: * Represents absolute Leucocytes Counts, ** Represents Relative Leucocytes Counts.

Individual Animal Coagulation Parameters

Sex: Male

Group	Animal Number	Parameters	
		PT (Sec)	APTT (Sec)
G1 (Vehicle: 0 mg/kg B.wt./day)	1	12.3	32.8
	2	13.2	29.1
	3	14.8	31.0
	4	12.8	31.7
	5	14.7	30.3
	6	14.1	28.8
	7	15.1	28.6
	8	14.8	28.2
	9	13.9	27.4
	10	14.9	28.9
G2 (Low Dose: 10.3 mg/kg B.wt./day)	21	15.0	30.2
	22	16.6	31.7
	23	15.8	28.9
	24	15.8	30.5
	25	16.5	30.6
	26	16.3	31.0
	27	14.2	29.3
	28	14.9	31.1
	29	15.4	29.4
	30	15.3	29.3

Individual Animal Coagulation Parameters

Sex: Male

Group	Animal Number	Parameters	
		PT (Sec)	APTT (Sec)
G3 (Mid Dose: 20.6 mg/kg B.wt./day)	41	15.3	34.9
	42	15.3	32.3
	43	16.0	20.5
	44	14.8	28.1
	45	18.3	25.3
	46	17.4	26.0
	47	16.6	27.8
	48	16.4	28.5
	49	14.2	26.7
	50	14.4	28.2
G4 (High Dose: 41.2 mg/kg B.wt./day)	61	15.2	26.4
	62	15.4	28.2
	63	15.7	27.2
	64	13.7	26.0
	65	13.2	25.4
	66	14.9	25.4
	67	14.4	34.6
	68	13.6	33.1
	69	13.4	31.2
	70	14.7	30.0

Individual Animal Coagulation Parameters

Sex: Male

Group	Animal Number	Parameters	
		PT (Sec)	APTT (Sec)
G5 (Recovery Vehicle: 0 mg/kg B.wt./day)	81	12.2	30.9
	82	11.9	30.0
	83	11.4	28.1
	84	12.0	31.7
	85	12.7	28.0
G6 (Recovery High Dose: 41.2 mg/kg B.wt./day)	91	12.7	29.8
	92	13.7	31.1
	93	14.0	32.6
	94	13.5	33.0
	95	13.6	33.4

Individual Animal Coagulation Parameters

Sex: Female

Group	Animal Number	Parameters	
		PT (Sec)	APTT (Sec)
G1 (Vehicle: 0 mg/kg B.wt./day)	11	13.5	31.6
	12	13.4	31.8
	13	14.3	30.8
	14	13.4	31.6
	15	13.9	32.0
	16	12.3	31.5
	17	13.2	31.3
	18	11.1	31.3
	19	12.8	31.0
	20	13.6	30.2
G2 (Low Dose: 10.3 mg/kg B.wt./day)	31	14.0	30.3
	32	14.5	30.3
	33	14.9	30.3
	34	14.7	30.3
	35	14.7	30.0
	36	14.7	30.4
	37	14.8	30.4
	38	15.1	30.0
	39	14.9	29.7
	40	13.7	29.4

Individual Animal Coagulation Parameters

Sex: Female

Group	Animal Number	Parameters	
		PT (Sec)	APTT (Sec)
G3 (Mid Dose: 20.6 mg/kg B.wt./day)	51	17.3	32.6
	52	17.1	32.2
	53	17.0	32.2
	54	16.8	33.1
	55	15.4	31.0
	56	14.7	32.1
	57	12.6	32.8
	58	13.9	31.5
	59	12.8	28.9
	60	10.2	28.6
G4 (High Dose: 41.2 mg/kg B.wt./day)	71	12.8	28.5
	72	14.2	27.8
	73	14.0	29.3
	74	14.2	30.4
	75	14.6	30.3
	76	15.5	30.3
	77	15.5	28.1
	78	15.2	27.8
	79	15.0	28.9
	80	14.6	29.8

Individual Animal Coagulation Parameters

Sex: Female

Group	Animal Number	Parameters	
		PT (Sec)	APTT (Sec)
G5 (Recovery Vehicle: 0 mg/kg B.wt./day)	86	13.3	30.6
	87	13.9	30.4
	88	14.2	31.2
	89	14.4	31.9
	90	13.0	28.9
G6 (Recovery High Dose: 41.2 mg/kg B.wt./day)	96	12.9	28.7
	97	13.8	31.1
	98	14.3	29.1
	99	15.0	30.3
	100	16.6	30.0
