# Supplementary material for: 90-day oral toxicity study of a salmon nasal cartilage extract containing undenatured collagen and proteoglycan in Sprague-Dawley rats
Source: PLoS One. 2026 Jan 23;21(1):e0340675. doi: 10.1371/journal.pone.0340675 (PMC12829970; doi:10.1371/journal.pone.0340675)
Supplement: S5 Data set — (PDF) [file pone.0340675.s005.pdf]

Individual Animal Clinical Chemistry Parameters

Group: G1 (Vehicle: 0 mg/kg B.wt./day) Sex: Male

Animal Number	GLU (mg/dL)	TP (g/L)	ALT (U/L)	AST (U/L)	ALP (IU/L)	BIL (mg/dL)	BUN (mg/dL)	CRE (mg/dL)	TRG (mg/dL)	CHO (mg/dL)
1	110.4	6.86	45.0	87.3	66.7	0.06	10.7	0.97	96.3	47.4
2	94.6	6.36	44.3	82.6	74.0	0.07	11.0	1.00	23.7	56.5
3	104.7	6.19	72.6	81.6	92.4	0.08	10.3	0.92	56.3	34.6
4	103.8	5.95	88.3	83.1	77.7	0.09	10.2	0.99	95.3	58.3
5	135.4	6.21	64.8	74.1	82.8	0.08	10.1	0.88	96.3	79.2
6	100.6	6.63	58.2	62.5	73.0	0.09	11.6	0.81	91.5	58.6
7	118.2	6.24	58.0	68.2	81.9	0.07	10.2	1.38	62.0	67.7
8	92.1	6.14	44.8	83.4	95.4	0.08	10.4	1.11	62.6	58.9
9	118.6	6.28	47.5	84.1	74.6	0.07	11.4	1.27	44.7	72.0
10	127.6	5.78	50.6	81.0	78.8	0.06	9.9	0.87	88.3	44.6

Animal Number	Na (mmol/L)	K (mmol/L)	P (mg/dL)	ALB (mg/dL)	Ca (mg/dL)	T3 (ng/mL)	T4 (ng/mL)	TSH (uIU/mL)	HDL (mg/dL)	LDL (mg/dL)
1	138.4	4.08	6.12	3.02	10.58	6	27	1	41.3	30.8
2	139.5	4.02	5.62	3.29	12.02	6	27	0	41.5	32.2
3	139.2	4.68	7.14	3.03	10.2	6	91	0	38.4	31.2
4	138.4	5.37	6.96	3.22	10.9	7	91	2	40.5	30.8
5	137.9	5.14	6.88	3.20	11.68	6	59	2	41.8	30.48
6	137.7	5.34	6.63	3.62	11.63	5	88	2	41.3	30.2
7	138.5	4.91	6.58	3.42	10.71	6	97	2	41.8	31.9
8	137.4	5.09	7.03	3.05	11.5	7	100	2	41.0	31.1
9	138.6	4.98	6.54	3.29	11.5	5	81	3	41.1	31.8
10	138.0	4.94	6.30	3.14	11.03	6	103	2	41.5	31.0

Individual Animal Clinical Chemistry Parameters

Group: G2 (Low Dose: 10.3 mg/kg B.wt./day) Sex: Male

Animal Number	GLU (mg/dL)	TP (g/L)	ALT (U/L)	AST (U/L)	ALP (IU/L)	BIL (mg/dL)	BUN (mg/dL)	CRE (mg/dL)	TRG (mg/dL)	CHO (mg/dL)
21	120.4	5.95	60.3	80.3	92.0	0.08	10.3	0.92	66.5	46.5
22	140.2	6.06	47.9	89.2	72.0	0.06	9.8	0.94	45.3	67.1
23	106.7	6.29	45.5	89.7	99.0	0.08	9.8	0.98	50.5	53.3
24	118.2	6.03	43.8	81.4	83.2	0.08	10.5	0.77	60.0	45.5
25	109.2	6.01	57.9	89.9	77.2	0.07	11.4	0.90	79.3	77.9
26	131.0	6.04	56.7	61.5	79.6	0.09	10.6	0.88	46.5	54.2
27	130.1	6.03	45.1	77.9	72.0	0.08	9.9	0.96	62.4	42.2
28	165.8	6.45	42.6	74.9	76.9	0.08	10.0	0.93	89.3	53.0
29	121.4	6.05	47.4	72.8	78.7	0.09	10.2	0.81	58.7	73.3
30	126.7	6.43	46.0	75.9	72.2	0.08	10.3	1.05	88.3	56.7

Animal Number	Na (mmol/L)	K (mmol/L)	P (mg/dL)	ALB (mg/dL)	Ca (mg/dL)	T3 (ng/mL)	T4 (ng/mL)	TSH (uIU/mL)	HDL (mg/dL)	LDL (mg/dL)
21	138.5	4.84	6.66	3.21	11.5	6	94	1	40.5	30.3
22	138.2	4.74	6.41	3.37	10.65	5	100	1	41.2	31.1
23	140.1	5.18	6.39	3.33	10.9	5	56	2	41.2	30.8
24	138.8	4.85	6.16	3.02	10.4	6	34	2	41.2	31.2
25	139.0	5.02	6.62	3.20	11.66	8	43	2	37.0	30.4
26	138.8	4.89	6.57	3.22	11.5	6	75	2	41.6	30.4
27	138.9	5.38	6.90	3.21	11.5	6	53	2	41.4	30.1
28	138.2	5.82	6.34	3.34	11.4	6	59	2	41.9	31.5
29	137.7	4.93	5.98	3.28	10.5	6	37	2	41.9	30.2
30	138.5	4.76	7.14	3.29	11.5	6	84	3	41.6	30.8

Individual Animal Clinical Chemistry Parameters

Group: G3 (Mid Dose: 20.6 mg/kg B.wt./day) Sex: Male

Animal Number	GLU (mg/dL)	TP (g/L)	ALT (U/L)	AST (U/L)	ALP (IU/L)	BIL (mg/dL)	BUN (mg/dL)	CRE (mg/dL)	TRG (mg/dL)	CHO (mg/dL)
41	108.7	5.50	46.9	62.8	83.5	0.08	9.4	1.89	91.6	57.3
42	126.9	6.21	47.6	74.8	76.1	0.08	11.3	0.85	20.7	52.7
43	150.3	6.21	39.2	85.8	80.7	0.07	9.9	0.92	33.6	49.0
44	133.9	5.78	42.1	89.5	83.1	0.09	9.9	0.85	91.6	47.0
45	122.6	6.25	48.1	72.9	78.2	0.08	10.0	0.91	18.9	78.7
46	129.4	6.07	45.7	72.3	89.8	0.09	10.1	0.85	65.3	39.2
47	154.9	6.07	43.2	82.3	85.5	0.08	10.6	1.02	76.5	38.4
48	128.8	6.07	40.0	65.6	92.1	0.08	9.9	0.92	85.6	64.4
49	162.9	5.94	45.0	87.6	82.7	0.08	10.2	0.98	83.0	47.9
50	136.6	6.16	44.3	72.6	83.0	0.09	10.6	1.23	66.6	63.6

Animal Number	Na (mmol/L)	K (mmol/L)	P (mg/dL)	ALB (mg/dL)	Ca (mg/dL)	T3 (ng/mL)	T4 (ng/mL)	TSH (uIU/mL)	HDL (mg/dL)	LDL (mg/dL)
41	138.5	4.52	6.36	3.22	10.05	7	103	2	41.2	29.5
42	138.8	4.76	6.92	3.58	10.11	6	43	2	41.2	32.0
43	139.8	4.75	6.93	3.39	10.6	6	53	2	41.0	30.1
44	138.7	4.18	7.02	3.38	11.2	7	56	2	41.0	30.6
45	140.2	4.71	6.87	3.54	10.22	7	62	2	41.1	32.9
46	138.6	4.98	5.99	3.49	10.5	6	53	2	41.5	31.6
47	139.9	4.99	6.49	3.66	10.5	6	59	2	41.7	29.2
48	137.0	4.77	6.79	3.50	10.5	7	46	2	42.3	30.7
49	139.7	4.79	6.82	3.59	11.2	6	65	2	41.3	30.9
50	139.0	4.58	6.13	3.49	10.5	6	46	2	41.7	30.1

Individual Animal Clinical Chemistry Parameters

Group: G4 (High Dose: 41.2 mg/kg B.wt./day) Sex: Male

Animal Number	GLU (mg/dL)	TP (g/L)	ALT (U/L)	AST (U/L)	ALP (IU/L)	BIL (mg/dL)	BUN (mg/dL)	CRE (mg/dL)	TRG (mg/dL)	CHO (mg/dL)
61	88.9	5.96	43.1	77.4	72.7	0.09	11.0	0.81	45.6	45.1
62	126.8	5.89	51.7	66.0	66.2	0.09	11.2	0.87	78.3	49.9
63	122.7	5.75	46.4	82.3	92.1	0.09	10.3	0.95	73.0	40.6
64	102.0	6.05	43.8	79.3	77.8	0.07	12.1	0.86	59.2	47.7
65	101.0	6.20	40.2	62.0	62.3	0.08	9.6	0.82	56.7	64.4
66	177.2	6.67	49.7	77.2	70.5	0.07	9.7	1.04	83.2	55.3
67	115.9	5.72	50.1	80.7	75.7	0.07	10.7	0.91	71.2	60.4
68	150.8	6.39	47.4	89.7	75.3	0.07	9.9	0.40	54.9	42.6
69	125.8	6.20	45.4	86.4	73.3	0.08	10.0	1.04	42.0	68.3
70	183.5	6.26	35.7	59.0	93.5	0.08	9.9	0.92	66.3	37.3

Animal Number	Na (mmol/L)	K (mmol/L)	P (mg/dL)	ALB (mg/dL)	Ca (mg/dL)	T3 (ng/mL)	T4 (ng/mL)	TSH (uIU/mL)	HDL (mg/dL)	LDL (mg/dL)
61	137.8	5.22	6.30	3.52	10.8	6	46	2	41.8	32.4
62	138.3	4.68	6.95	3.15	10.5	7	62	2	41.9	30.5
63	139.3	4.85	8.00	3.44	11.2	6	53	2	40.6	29.4
64	138.0	4.86	7.83	3.57	11.5	6	72	3	42.5	32.1
65	139.8	4.65	7.86	3.54	12.5	8	103	2	41.7	30.1
66	139.3	6.45	7.35	3.76	11.3	6	81	2	41.0	31.1
67	139.6	4.60	6.92	3.52	10.5	6	94	2	41.6	31.7
68	138.7	4.46	6.94	3.61	12.5	7	53	2	41.2	31.8
69	139.6	4.68	6.30	3.55	11.5	7	34	3	41.6	31.7
70	138.4	4.49	6.73	3.55	11.5	6	24	2	40.4	31.1

Individual Animal Clinical Chemistry Parameters

Group: G5 (Recovery Vehicle: 0 mg/kg B.wt./day) Sex: Male

Animal Number	GLU (mg/dL)	TP (g/L)	ALT (U/L)	AST (U/L)	ALP (IU/L)	BIL (mg/dL)	BUN (mg/dL)	CRE (mg/dL)	TRG (mg/dL)	CHO (mg/dL)
81	99.0	7.28	33.8	56.6	105.7	0.31	10.3	0.87	57.9	66.6
82	92.5	7.03	29.9	55.1	101.1	0.37	10.9	0.72	49.2	63.2
83	93.4	6.52	34.8	52.5	94.4	0.25	9.4	0.65	53.4	68.9
84	97.7	5.23	34.6	41.2	99.5	0.28	10.9	0.62	49.8	57.6
85	100.6	7.98	29.3	43.0	104.5	0.22	10.0	0.53	40.1	55.1

Animal Number	Na (mmol/L)	K (mmol/L)	P (mg/dL)	ALB (mg/dL)	Ca (mg/dL)	T3 (ng/mL)	T4 (ng/mL)	TSH (uIU/mL)	HDL (mg/dL)	LDL (mg/dL)
81	140.6	4.61	6.11	3.98	11.91	7	99	1	40.9	32.2
82	140.3	4.92	6.47	4.15	11.00	7	90	1	34.0	24.9
83	140.1	4.72	6.11	4.13	10.00	8	106	1	39.5	36.5
84	139.8	4.76	6.50	3.94	11.29	7	106	2	44.2	39.2
85	138.5	5.20	7.01	3.96	10.09	9	99	2	48.8	35.1

Group: G6 (Recovery High Dose: 41.2 mg/kg B.wt./day) Sex: Male

Animal Number	GLU (mg/dL)	TP (g/L)	ALT (U/L)	AST (U/L)	ALP (IU/L)	BIL (mg/dL)	BUN (mg/dL)	CRE (mg/dL)	TRG (mg/dL)	CHO (mg/dL)
91	92.6	7.51	44.5	53.8	101.7	0.26	9.9	0.57	54.2	69.3
92	95.3	8.26	42.7	50.4	93.8	0.34	9.5	0.50	101.5	66.1
93	97.0	8.26	40.8	53.3	94.1	0.26	23.5	0.68	58.9	67.4
94	99.2	8.15	33.9	46.2	109.9	0.35	10.8	0.59	51.3	66.9
95	96.3	7.52	40.6	48.1	107.0	0.28	10.6	0.51	53.5	69.6

Animal Number	Na (mmol/L)	K (mmol/L)	P (mg/dL)	ALB (mg/dL)	Ca (mg/dL)	T3 (ng/mL)	T4 (ng/mL)	TSH (uIU/mL)	HDL (mg/dL)	LDL (mg/dL)
91	141.3	6.36	6.45	3.97	8.80	7	103	2	41.0	33.2
92	141.5	6.60	6.29	4.17	12.00	7	90	2	38.0	37.6
93	138.1	4.95	5.83	4.14	11.29	8	106	2	41.1	28.6
94	138.9	5.17	5.51	4.17	11.21	7	106	2	38.1	30.1
95	139.2	5.18	5.73	3.98	9.78	7	96	2	34.9	27.6

Individual Animal Clinical Chemistry Parameters

Group: G1 (Vehicle: 0 mg/kg B.wt./day) Sex: Female

Animal Number	GLU (mg/dL)	TP (g/L)	ALT (U/L)	AST (U/L)	ALP (IU/L)	BIL (mg/dL)	BUN (mg/dL)	CRE (mg/dL)	TRG (mg/dL)	CHO (mg/dL)
11	91.0	6.41	41.0	57.3	75.0	0.09	10.0	1.13	78.3	76.5
12	91.8	6.80	47.4	86.7	80.9	0.07	10.6	1.19	78.2	89.1
13	96.0	6.80	51.6	83.5	81.2	0.09	10.0	1.02	45.6	47.3
14	74.7	6.97	51.0	83.9	87.7	0.06	10.3	1.11	78.9	56.7
15	92.9	6.89	51.0	61.9	88.0	0.07	10.5	1.11	78.6	70.3
16	106.0	6.71	40.2	64.0	78.0	0.08	11.8	1.00	71.3	54.2
17	74.3	7.24	49.8	85.4	67.5	0.09	11.8	1.24	73.6	47.2
18	115.3	7.22	47.0	81.2	85.8	0.08	12.3	1.03	70.3	56.8
19	100.3	6.73	32.9	91.8	79.9	0.06	10.4	1.18	80.3	69.8
20	117.4	6.50	41.1	60.8	69.2	0.07	11.5	1.38	69.3	42.4

Animal Number	Na (mmol/L)	K (mmol/L)	P (mg/dL)	ALB (mg/dL)	Ca (mg/dL)	T3 (ng/mL)	T4 (ng/mL)	TSH (uIU/mL)	HDL (mg/dL)	LDL (mg/dL)
11	138.5	5.07	6.72	3.58	10.8	9	89	2	41.5	31.1
12	137.8	3.91	6.23	3.61	11.2	9	95	1	41.7	31.7
13	139.1	4.80	5.52	3.44	11.5	7	95	1	31.4	31.6
14	138.0	4.99	6.10	3.76	11.5	10	86	2	41.1	29.5
15	137.5	4.58	6.06	3.71	11.8	5	77	1	41.8	32.3
16	138.2	4.44	5.52	3.71	11.1	9	55	1	40.5	31.5
17	138.0	4.63	6.42	3.70	11.5	8	89	2	41.3	30.5
18	139.3	4.76	6.53	3.66	10.2	10	80	2	40.5	29.9
19	138.9	4.60	6.10	3.59	11.5	8	71	2	41.7	31.8
20	139.0	4.40	5.81	3.61	11.9	6	77	2	41.3	27.5

Individual Animal Clinical Chemistry Parameters

Group: G2 (Low Dose: 10.3 mg/kg B.wt./day) Sex: Female

Animal Number	GLU (mg/dL)	TP (g/L)	ALT (U/L)	AST (U/L)	ALP (IU/L)	BIL (mg/dL)	BUN (mg/dL)	CRE (mg/dL)	TRG (mg/dL)	CHO (mg/dL)
31	138.8	5.30	42.5	80.8	74.2	0.07	10.9	1.15	72.3	54.7
32	106.2	6.70	46.8	84.3	96.7	0.05	10.3	1.36	58.6	55.6
33	109.6	6.75	46.6	82.2	83.6	0.09	9.8	1.37	85.6	87.4
34	111.0	6.72	46.1	67.1	95.4	0.05	11.0	1.04	69.9	65.2
35	86.3	6.93	46.3	66.5	72.3	0.08	12.0	1.11	24.5	54.5
36	83.5	7.36	43.7	73.8	94.2	0.08	11.8	1.10	57.2	65.3
37	95.6	6.91	41.6	64.8	87.0	0.08	11.1	1.11	78.9	45.7
38	88.4	7.03	68.4	83.0	65.8	0.06	10.4	1.18	62.3	89.7
39	95.2	6.89	68.9	76.7	77.0	0.08	10.2	1.20	72.8	55.9
40	123.0	6.54	54.8	92.1	87.6	0.08	9.9	0.97	72.3	44.2

Animal Number	Na (mmol/L)	K (mmol/L)	P (mg/dL)	ALB (mg/dL)	Ca (mg/dL)	T3 (ng/mL)	T4 (ng/mL)	TSH (uIU/mL)	HDL (mg/dL)	LDL (mg/dL)
31	138.4	4.10	6.06	3.42	10.5	7	68	2	41.9	29.1
32	138.0	4.09	6.48	3.70	11.4	9	89	2	41.6	29.2
33	138.3	4.94	6.27	3.68	11.8	9	92	2	41.8	29.9
34	139.3	4.28	5.72	3.82	12.4	9	83	2	41.8	28.0
35	138.2	4.33	5.30	3.69	11.2	8	83	2	41.6	30.7
36	138.3	4.67	6.11	3.62	10.6	8	83	1	41.3	29.7
37	139.5	4.38	6.55	3.75	11.5	7	52	2	40.1	31.9
38	140.4	3.98	6.02	3.55	11.8	9	89	2	40.4	28.2
39	140.4	4.45	5.95	3.61	10.6	5	89	1	41.0	30.1
40	137.5	4.64	5.55	3.70	10.5	7	65	2	41.3	30.3

Individual Animal Clinical Chemistry Parameters

Group: G3 (Mid Dose: 20.6 mg/kg B.wt./day) Sex: Female

Animal Number	GLU (mg/dL)	TP (g/L)	ALT (U/L)	AST (U/L)	ALP (IU/L)	BIL (mg/dL)	BUN (mg/dL)	CRE (mg/dL)	TRG (mg/dL)	CHO (mg/dL)
51	86.6	6.15	42.5	77.8	60.3	0.09	10.7	0.78	71.5	66.5
52	111.4	5.18	44.2	89.3	76.8	0.06	9.9	0.92	53.6	89.8
53	72.0	5.85	46.3	51.0	67.7	0.08	10.8	0.91	91.5	83.3
54	92.6	5.98	42.1	86.9	58.6	0.07	20.6	0.87	77.3	95.5
55	105.9	6.11	44.5	68.0	96.9	0.08	10.1	0.85	78.9	78.5
56	119.7	5.82	43.7	87.3	74.5	0.09	10.6	0.87	77.5	79.1
57	110.5	6.74	47.4	65.4	98.0	0.08	11.5	0.77	73.8	71.5
58	117.1	7.09	42.0	86.9	90.9	0.07	11.1	0.79	82.3	83.9
59	106.1	5.85	42.5	80.9	77.9	0.08	10.6	0.83	69.3	71.0
60	78.8	6.12	46.2	61.7	94.8	0.07	9.7	0.72	70.2	87.2

Animal Number	Na (mmol/L)	K (mmol/L)	P (mg/dL)	ALB (mg/dL)	Ca (mg/dL)	T3 (ng/mL)	T4 (ng/mL)	TSH (uIU/mL)	HDL (mg/dL)	LDL (mg/dL)
51	136.9	4.16	5.38	3.35	10.5	5	68	2	41.2	28.4
52	139.6	4.47	6.45	3.32	11.8	9	74	1	40.4	31.5
53	138.6	4.85	6.14	3.29	11.6	7	83	2	41.6	31.7
54	138.8	4.92	6.46	3.31	11.5	5	65	2	42.2	29.3
55	138.8	4.83	6.40	3.39	10.9	8	68	2	41.8	29.7
56	137.8	4.82	6.11	3.41	11.6	7	74	2	41.6	31.0
57	138.9	4.01	6.49	3.06	11.2	8	95	2	41.6	29.1
58	138.7	4.82	6.33	3.51	11.8	7	49	2	42.7	31.4
59	138.5	4.63	5.51	3.11	11.6	7	77	2	40.6	30.4
60	138.9	4.76	5.64	3.36	12.6	9	65	1	40.7	32.3

Individual Animal Clinical Chemistry Parameters

Group: G4 (High Dose: 41.2 mg/kg B.wt./day) Sex: Female

Animal Number	GLU (mg/dL)	TP (g/L)	ALT (U/L)	AST (U/L)	ALP (IU/L)	BIL (mg/dL)	BUN (mg/dL)	CRE (mg/dL)	TRG (mg/dL)	CHO (mg/dL)
71	119.5	6.16	44.3	65.9	95.2	0.06	10.7	0.72	70.5	72.2
72	76.6	5.32	46.8	88.1	88.9	0.07	10.6	0.81	61.8	79.1
73	84.1	6.05	42.1	80.7	62.3	0.09	10.3	0.69	20.3	72.1
74	116.6	6.90	44.6	82.6	79.1	0.08	11.8	0.72	73.6	96.2
75	104.2	5.75	42.7	80.3	67.3	0.09	10.2	0.75	73.6	85.6
76	96.8	6.46	46.1	66.4	76.6	0.07	11.9	0.78	96.3	67.6
77	97.1	6.49	45.7	81.6	63.1	0.08	11.6	0.74	97.0	76.2
78	109.5	6.29	46.3	98.1	78.0	0.09	10.4	0.80	80.6	80.8
79	115.0	6.09	45.6	80.9	84.6	0.09	11.3	0.73	58.1	86.4
80	99.4	6.30	47.7	97.8	93.1	0.08	11.3	0.82	98.1	89.1

Animal Number	Na (mmol/L)	K (mmol/L)	P (mg/dL)	ALB (mg/dL)	Ca (mg/dL)	T3 (ng/mL)	T4 (ng/mL)	TSH (uIU/mL)	HDL (mg/dL)	LDL (mg/dL)
71	138.5	3.99	6.09	3.41	12.1	9	89	2	41.9	29.8
72	138.4	4.49	6.45	3.16	12.8	8	77	1	41.2	30.8
73	139.3	4.61	6.17	3.26	11.8	9	71	2	36.5	28.4
74	138.4	4.76	5.86	3.69	10.9	7	49	2	41.2	29.4
75	138.1	4.81	6.52	3.39	11.8	7	62	2	41.6	30.4
76	139.6	5.09	5.75	3.42	12.1	6	46	1	42.0	28.4
77	140.0	5.99	5.35	3.27	12.8	5	98	2	32.5	28.9
78	137.7	4.97	5.66	3.69	11.5	6	68	2	41.5	30.4
79	138.1	4.95	6.23	3.43	12.9	7	83	2	41.4	29.5
80	139.9	5.05	6.18	3.57	11.2	7	74	2	41.2	29.0

Individual Animal Clinical Chemistry Parameters

Group: G5 (Recovery Vehicle: 0 mg/kg B.wt./day) Sex: Female

Animal Number	GLU (mg/dL)	TP (g/L)	ALT (U/L)	AST (U/L)	ALP (IU/L)	BIL (mg/dL)	BUN (mg/dL)	CRE (mg/dL)	TRG (mg/dL)	CHO (mg/dL)
86	96.8	7.65	39.9	57.2	99.2	0.31	11.2	0.63	49.0	52.0
87	98.3	7.02	48.2	56.3	92.4	0.29	11.1	0.69	46.5	53.9
88	93.8	6.58	39.3	46.2	101.1	0.43	10.2	0.55	42.7	52.5
89	99.8	6.00	48.4	53.5	94.8	0.30	12.0	0.56	49.4	57.7
90	96.1	6.91	42.4	52.7	90.1	0.27	11.8	0.67	56.4	60.3

Animal Number	Na (mmol/L)	K (mmol/L)	P (mg/dL)	ALB (mg/dL)	Ca (mg/dL)	T3 (ng/mL)	T4 (ng/mL)	TSH (uIU/mL)	HDL (mg/dL)	LDL (mg/dL)
86	137.6	5.23	6.24	4.38	10.00	9	87	2	25.3	21.8
87	136.5	4.59	5.72	4.88	9.29	9	96	1	47.6	30.1
88	138.7	4.55	5.05	4.63	11.19	10	81	1	43.2	23.6
89	138.3	4.46	5.63	5.10	12.00	10	87	2	46.6	32.0
90	139.1	4.83	5.56	4.58	11.87	10	90	2	26.6	18.7

Group: G6 (Recovery High Dose: 41.2 mg/kg B.wt./day) Sex: Female

Animal Number	GLU (mg/dL)	TP (g/L)	ALT (U/L)	AST (U/L)	ALP (IU/L)	BIL (mg/dL)	BUN (mg/dL)	CRE (mg/dL)	TRG (mg/dL)	CHO (mg/dL)
96	97.7	7.21	53.3	66.0	96.7	0.30	10.6	0.53	43.9	62.4
97	93.0	6.59	58.8	62.5	102.3	0.31	10.6	0.57	37.0	46.3
98	99.1	6.44	52.8	63.1	96.9	0.40	9.9	0.78	53.0	61.6
99	90.3	6.55	41.6	50.4	99.9	0.44	26.5	0.72	45.2	54.5
100	93.5	6.89	39.2	44.8	98.2	0.26	9.6	0.67	52.9	61.4

Animal Number	Na (mmol/L)	K (mmol/L)	P (mg/dL)	ALB (mg/dL)	Ca (mg/dL)	T3 (ng/mL)	T4 (ng/mL)	TSH (uIU/mL)	HDL (mg/dL)	LDL (mg/dL)
96	138.6	4.35	5.82	5.06	11.29	9	84	2	47.9	25.3
97	138.6	4.86	5.64	4.36	11.94	9	99	2	47.7	34.5
98	138.3	4.79	5.37	5.34	12.87	10	96	2	50.6	28.5
99	139.3	4.70	5.02	4.39	10.34	9	90	2	48.7	38.2
100	137.7	4.53	4.87	5.33	12.11	9	90	2	42.5	30.3

Individual Absolute Organ Weights (g)

Group: G1 (Vehicle: 0 mg/kg B.wt./day) Sex: Male

Animal Number	Adrenals*	Kidneys*	Liver	Heart	Brain	Spleen	Epididymis*
1	0.0533	1.8740	8.2121	1.0151	1.8224	0.7489	14862
2	0.0628	3.0948	13.4319	1.2448	1.9759	1.2277	1.5709
3	0.0494	2.5130	9.6171	1.1961	1.9963	0.6814	1.4125
4	0.0638	2.6645	10.9239	1.1908	1.8208	0.8549	1.2303
5	0.0629	2.2684	9.9541	1.0409	1.9004	0.6278	1.4351
6	0.0615	2.9567	10.8109	1.1632	1.9052	0.8738	1.3275
7	0.0567	3.1730	13.1187	1.4157	1.9721	1.3439	1.4994
8	0.0612	2.9103	9.5905	1.1413	1.9009	0.8079	1.3508
9	0.0570	2.7659	13.6432	1.1280	1.8894	0.8127	1.5907
10	0.0628	3.1587	14.5046	1.2275	1.8882	0.9991	1.3051

Animal Number	Testes*	Thymus	Thyroid Gland	Seminal Vesicle	Prostate + Seminal Vesicles with Coagulating Glands	Prostate	Pituitary Gland
1	2.9831	0.4340	0.0021	0.4578	1.0781	0.6401	0.0141
2	2.8853	0.2987	0.0027	0.4725	1.0783	0.6311	0.0132
3	2.8775	0.2936	0.0023	0.4703	1.0784	0.6431	0.0114
4	3.2427	0.4709	0.0021	0.4699	1.0786	0.6443	0.0131
5	2.9057	0.4215	0.0026	0.4725	1.0774	0.6445	0.0130
6	3.0625	0.3806	0.0025	0.4712	1.0771	0.6409	0.0121
7	3.1913	0.3764	0.0027	0.4825	1.0739	0.6431	0.0112
8	2.8732	0.2335	0.0024	0.4769	1.0743	0.6413	0.0121
9	2.9809	0.4820	0.0026	0.4802	1.0707	0.6407	0.0114
10	2.9571	0.6947	0.0025	0.4512	1.0731	0.6431	0.0117

Note: * Paired organs were weighed together.

Individual Absolute Organ Weights (g)

Group: G2 (Low Dose: 10.3 mg/kg B.wt./day) Sex: Male

Animal Number	Adrenals*	Kidneys*	Liver	Heart	Brain	Spleen	Epididymis*
21	0.0564	2.9243	11.9422	1.2153	1.8343	0.5210	2.2127
22	0.0538	3.1182	10.9833	1.2704	1.9787	0.5721	1.0997
23	0.0634	3.1655	14.3628	1.2080	2.1982	0.5156	1.1336
24	0.0507	2.7173	10.1003	1.1869	1.7799	0.4863	1.4040
25	0.0673	3.7958	17.6384	1.3704	1.6879	0.9302	1.3851
26	0.0780	2.8164	11.3435	1.1083	1.6883	0.5684	1.4232
27	0.0885	2.8710	12.5095	1.2078	1.7845	0.6575	1.5083
28	0.0577	2.9548	13.7875	1.3750	1.7307	0.7532	1.2925
29	0.0604	2.7909	12.8705	1.3601	1.8731	0.6705	1.6421
30	0.0613	2.6972	17.7315	1.0275	1.6403	0.7864	1.1060

Animal Number	Testes*	Thymus	Thyroid Gland	Seminal Vesicle	Prostate + Seminal Vesicles with Coagulating Glands	Prostate	Pituitary Gland
21	3.4960	0.3602	0.0024	0.4175	1.0754	0.6391	0.0131
22	2.1789	0.4571	0.0023	0.4162	1.0749	0.6439	0.0132
23	3.0742	0.3246	0.0023	0.4821	1.0731	0.6413	0.0134
24	2.7839	0.2693	0.0024	0.4725	1.0739	0.6454	0.0140
25	2.5081	0.6268	0.0029	0.4665	1.0746	0.6491	0.0141
26	2.2572	0.6895	0.0031	0.4721	1.0744	0.7011	0.0136
27	3.5891	0.5117	0.0025	0.4578	1.0731	0.6931	0.0133
28	2.6567	0.4282	0.0024	0.4215	1.0743	0.6937	0.0131
29	2.7565	0.4305	0.0021	0.4265	1.0754	0.6739	0.0130
30	3.1348	0.5925	0.0021	0.4287	1.0731	0.6811	0.0124

Note: * Paired organs were weighed together.

Individual Absolute Organ Weights (g)

Group: G3 (Mid Dose: 20.6 mg/kg B.wt./day) Sex: Male

Animal Number	Adrenals*	Kidneys*	Liver	Heart	Brain	Spleen	Epididymis*
41	0.0579	2.5329	11.9805	1.1578	1.6994	0.6073	1.3872
42	0.0528	2.6321	11.7831	1.1802	1.7782	0.7825	1.4852
43	0.0501	2.6545	11.4297	1.0417	1.9119	0.5802	1.4827
44	0..0605	2.5702	12.7892	1.1572	1.3215	0.7419	1.2988
45	0.0537	2.7825	13.8701	1.1208	1.5690	0.6502	1.4760
46	0.0621	3.3669	14.2881	1.1130	1.8251	0.6429	1.3572
47	0.0598	2.8079	13.8707	1.3451	1.7734	0.6570	1.4672
48	0.0572	2.7332	10.7551	1.0143	1.6401	0.6963	1.4541
49	0.0635	2.7082	11.9805	1.2725	1.7825	0.6521	1.4821
50	0.0568	2.4485	10.8536	0.9996	2.0322	0.7478	1.2469

Animal Number	Testes*	Thymus	Thyroid Gland	Seminal Vesicle	Prostate + Seminal Vesicles with Coagulating Glands	Prostate	Pituitary Gland
41	3.0105	0.3492	0.0029	0.4587	1.0731	0.6841	0.0131
42	2.8115	0.4821	0.0026	0.4725	1.0749	0.6954	0.0134
43	3.0175	0.2076	0.0024	0.4698	1.0737	0.6191	0.0141
44	3.0572	0.4538	0.0021	0.4821	1.0746	0.6301	0.0140
45	2.8924	0.4531	0.0026	0.4579	1.0761	0.6191	0.0129
46	2.9725	0.5601	0.0027	0.4705	1.0731	0.6319	0.0124
47	2.9605	0.6732	0.0031	0.4698	1.0739	0.6139	0.0126
48	3.1496	0.4592	0.0029	0.4702	1.0727	0.6419	0.0129
49	2.8905	0.5731	0.0027	0.4565	1.0729	0.6354	0.0123
50	2.8722	0.6287	0.0021	0.4752	1.0741	0.6401	0.0119

Note: * Paired organs were weighed together.

Individual Absolute Organ Weights (g)

Group: G4 (High Dose: 41.2 mg/kg B.wt./day) Sex: Male

Animal Number	Adrenals*	Kidneys*	Liver	Heart	Brain	Spleen	Epididymis*
61	0.0622	3.7219	17.6763	1.4060	2.1574	1.5304	1.5125
62	0.0652	2.6521	15.7535	1.3801	1.9752	0.8731	1.4275
63	0.0615	2.7605	14.7605	1.2592	1.8708	0.9567	1.4525
64	0.0592	2.6903	12.5850	0.9636	1.6921	0.8889	1.3432
65	0.0631	3.4362	15.6321	1.2274	1.1658	0.8509	1.4118
66	0.0598	3.4055	17.2777	1.1564	1.7508	0.8737	1.3982
67	0.0756	2.4503	12.7386	1.1548	1.8123	0.6871	1.2734
68	0.0598	2.5678	13.7342	1.1367	1.8059	0.8349	1.4585
69	0.0608	2.8515	14.1592	1.1755	1.8751	0.7521	1.3032
70	0.0701	3.2772	13.3435	1.3769	1.8561	0.7864	1.3851

Animal Number	Testes*	Thymus	Thyroid Gland	Seminal Vesicle	Prostate + Seminal Vesicles with Coagulating Glands	Prostate	Pituitary Gland
61	3.6352	0.4155	0.0029	0.4851	1.0743	0.6919	0.0134
62	3.1251	0.5172	0.0026	0.4256	1.0741	0.6907	0.0131
63	3.2671	0.4581	0.0025	0.4725	1.0739	0.6091	0.0129
64	3.0846	0.5081	0.0021	0.4658	1.0724	0.6154	0.0141
65	2.9518	0.6346	0.0027	0.4721	1.0739	0.6201	0.0131
66	3.1905	0.4891	0.0026	0.4898	1.0743	0.6154	0.0136
67	3.0678	0.3565	0.0029	0.4257	1.0719	0.6093	0.0141
68	3.0521	0.4828	0.0027	0.4689	1.0724	0.6191	0.0131
69	2.9621	0.5095	0.0026	0.4725	1.0716	0.6311	0.0134
70	3.2820	0.8765	0.0024	0.4587	1.0721	0.6195	0.0129

Note: * Paired organs were weighed together.

Individual Absolute Organ Weights (g)

Group: G5 (Recovery Vehicle: 0 mg/kg B.wt./day) Sex: Male

Animal Number	Adrenals*	Kidneys*	Liver	Heart	Brain	Spleen	Epididymis*
81	0.0719	3.6292	14.9174	1.3061	2.3912	0.9747	1.3468
82	0.0772	4.0976	15.9742	1.3947	2.0436	0.8126	1.4507
83	0.0679	2.9812	10.8288	1.2826	1.9494	0.8749	1.4235
84	0.0734	2.9961	11.8942	1.2075	2.0826	0.9028	1.5575
85	0.0783	3.2825	11.9091	1.3216	1.9335	0.7533	1.3486

Animal Number	Testes*	Thymus	Thyroid Gland	Seminal Vesicle	Prostate + Seminal Vesicles with Coagulating Glands	Prostate	Pituitary Gland
81	3.1224	0.4465	0.0026	0.4698	1.0739	0.6515	0.0121
82	3.0338	0.4835	0.0030	0.4787	1.0716	0.6391	0.0141
83	2.9826	0.5436	0.0024	0.4587	1.0724	0.6451	0.0129
84	3.1301	0.4705	0.0023	0.4578	1.0754	0.6311	0.0131
85	2.9483	0.3376	0.0025	0.4687	1.0716	0.6541	0.0140

Group: G6 (Recovery High Dose: 41.2 mg/kg B.wt./day) Sex: Male

Animal Number	Adrenals*	Kidneys*	Liver	Heart	Brain	Spleen	Epididymis*
91	0.0820	3.3275	11.7497	1.1336	1.8650	0.6053	1.4203
92	0.0626	3.7810	10.9051	1.2764	2.0407	0.8467	1.5368
93	0.0749	2.5628	12.2808	1.1516	1.8849	0.7608	1.4651
94	0.0714	3.5082	10.3699	1.2926	1.9303	0.7512	1.4271
95	0.0777	3.8802	10.9818	1.1836	1.9457	0.8907	1.4045

Animal Number	Testes*	Thymus	Thyroid Gland	Seminal Vesicle	Prostate + Seminal Vesicles with Coagulating Glands	Prostate	Pituitary Gland
91	3.1534	0.5807	0.0027	0.4257	1.0731	0.6911	0.0139
92	3.0590	0.3588	0.0025	0.4725	1.0754	0.6319	0.0127
93	2.9578	0.4775	0.0026	0.4821	1.0716	0.6511	0.0131
94	2.8507	0.4406	0.0024	0.4623	1.0729	0.6911	0.0141
95	3.1513	0.4389	0.0025	0.4287	1.0731	0.6391	0.0131

Note: * Paired organs were weighed together.

Individual Absolute Organ Weights (g)

Group: G1 (Vehicle: 0 mg/kg B.wt./day) Sex: Female

Animal Number	Adrenals*	Kidneys*	Liver	Heart	Brain	Spleen
11	0.0778	1.5866	8.9789	0.7979	1.5160	0.6721
12	0.0850	1.7195	7.5157	0.8817	1.7302	0.6692
13	0.0825	1.6288	8.4485	0.7588	1.6170	0.8630
14	0.0723	1.5831	8.7351	0.8890	1.5567	0.5963
15	0.0831	1.5642	7.5645	0.7050	1.7705	0.8732
16	0.0748	1.6763	7.5752	0.8515	1.5104	0.5335
17	0.0743	1.6732	7.8963	0.6543	1.3673	0.7543
18	0.0563	1.7531	8.5672	0.8721	1.4389	0.7564
19	0.0949	2.0705	7.8379	0.9230	1.7517	0.6287
20	0.0843	1.8531	7.8757	0.9031	1.6052	0.6893

Animal Number	Ovaries*	Uterus with Cervix	Thymus	Thyroid Gland	Pituitary Glands
11	0.1408	0.6397	0.2906	0.0027	0.0139
12	0.1159	0.8043	0.2529	0.0025	0.0140
13	0.1467	0.5256	0.4255	0.0026	0.0127
14	0.1123	0.5503	0.5981	0.0024	0.0141
15	0.1756	0.7560	0.3059	0.0023	0.0137
16	0.1354	0.9678	0.3078	0.0025	0.0139
17	0.1802	0.7449	0.3780	0.0027	0.0141
18	0.1567	0.8573	0.4531	0.0026	0.0138
19	0.1447	0.4206	0.4470	0.0024	0.0137
20	0.1354	0.5732	0.3521	0.0023	0.0141

Note: * Paired organs were weighed together.

Individual Absolute Organ Weights (g)

Group: G2 (Low Dose: 10.3 mg/kg B.wt./day) Sex: Female

Animal Number	Adrenals*	Kidneys*	Liver	Heart	Brain	Spleen
31	0.0893	1.7664	7.8336	0.9164	1.5605	0.6556
32	0.0741	1.8995	6.9172	0.9486	1.8860	0.5011
33	0.0729	1.7551	7.0819	0.8782	1.8960	0.5661
34	0.0881	1.5630	7.8735	0.8235	1.9903	0.3970
35	0.0609	1.6931	8.2567	0.8439	1.7831	0.4562
36	0.0790	1.6903	8.5630	0.8835	1.6221	0.3973
37	0.0737	1.5989	6.9648	0.8224	1.6115	0.4340
38	0.0724	1.5432	8.6732	0.8080	1.7632	0.5562
39	0.0753	1.7503	9.0105	0.9312	1.7532	0.4810
40	0.0693	1.7321	8.8031	0.7503	1.7531	0.4205

Animal Number	Ovaries*	Uterus with Cervix	Thymus	Thyroid Gland	Pituitary Glands
31	0.1691	0.6229	0.6390	0.0031	0.0137
32	0.1473	0.7547	0.3678	0.0027	0.0141
33	0.1532	0.5550	0.3231	0.0026	0.0140
34	0.1601	0.5801	0.4808	0.0026	0.0137
35	0.1355	0.6642	0.5531	0.0025	0.0129
36	0.1393	0.6705	0.5832	0.0024	0.0133
37	0.1207	0.4878	0.4671	0.0023	0.0140
38	0.1568	0.5698	0.5524	0.0024	0.0141
39	0.1232	0.7531	0.6562	0.0025	0.0131
40	0.1905	0.5632	0.5328	0.0027	0.0133

Note: * Paired organs were weighed together.

Individual Absolute Organ Weights (g)

Group: G3 (Mid Dose: 20.6 mg/kg B.wt./day)

Sex: Female

Animal Number	Adrenals*	Kidneys*	Liver	Heart	Brain	Spleen
51	0.0631	2.2799	10.4664	1.0472	1.7712	1.0596
52	0.0721	1.5601	7.5209	0.9095	1.7267	0.5147
53	0.1164	2.0492	9.4743	0.9496	1.7956	0.6484
54	0.0774	1.5769	7.2602	0.8167	1.8841	0.6068
55	0.0720	1.9821	7.5513	0.7976	1.3242	0.6626
56	0.0811	2.1105	7.5321	1.3651	1.8931	0.7351
57	0.0832	2.3210	7.5621	1.2210	1.8051	0.7821
58	0.0756	1.9801	8.8081	1.2531	1.9031	0.5531
59	0.0693	2.1523	8.0118	1.0657	1.9073	0.6033
60	0.0940	1.9297	7.8682	0.9349	1.8015	0.6455

Animal Number	Ovaries*	Uterus with Cervix	Thymus	Thyroid Gland	Pituitary Glands
51	0.1569	0.4732	0.7829	0.0034	0.0131
52	0.1276	0.5369	0.4075	0.0027	0.0133
53	0.1812	0.2758	0.5311	0.0031	0.0134
54	0.1508	0.7855	0.4941	0.0030	0.0137
55	0.1321	0.5212	0.5321	0.0027	0.0141
56	0.1321	0.5231	0.5532	0.0026	0.0140
57	0.1632	0.5621	0.4110	0.0029	0.0141
58	0.1792	0.4505	0.3982	0.0031	0.0143
59	0.1821	0.4995	0.4051	0.0027	0.0139
60	0.1021	0.5018	0.2729	0.0026	0.0139

Note: * Paired organs were weighed together.

Individual Absolute Organ Weights (g)

Group: G4 (High Dose: 41.2 mg/kg B.wt./day) Sex: Male

Animal Number	Adrenals*	Kidneys*	Liver	Heart	Brain	Spleen
71	0.0721	1.7319	8.2744	0.8134	1.2578	0.6856
72	0.0752	2.2684	7.9904	0.8850	1.6114	1.0314
73	0.0733	1.8082	8.0567	0.9313	1.5919	0.5675
74	0.0629	2.6284	13.2617	0.7844	1.7321	0.5950
75	0.0573	1.6846	7.3144	0.8729	1.6741	0.9341
76	0.0531	2.1348	8.9101	1.1316	1.7021	0.6694
77	0.0751	1.9753	8.5321	0.8701	1.5823	0.7511
78	0.0672	2.0571	8.7082	0.9504	1.6547	0.6280
79	0.0609	1.6532	7.9953	0.8429	1.5790	0.6846
80	0.0731	1.8037	7.6382	0.9291	1.5221	0.6247

Animal Number	Ovaries*	Uterus with Cervix	Thymus	Thyroid Gland	Pituitary Glands
71	0.1592	0.3081	0.2739	0.0027	0.0137
72	0.1318	0.6649	0.2726	0.0028	0.0136
73	0.1905	0.4785	0.6029	0.0027	0.0141
74	0.1618	0.4457	0.4873	0.0026	0.0140
75	0.1440	0.8601	0.4696	0.0025	0.0139
76	0.1521	0.7052	0.4100	0.0021	0.0138
77	0.1571	0.5121	0.4231	0.0023	0.0139
78	0.1651	0.5808	0.4825	0.0029	0.0141
79	0.1531	0.4316	0.4120	0.0032	0.0143
80	0.1309	0.7006	0.4856	0.0030	0.0144

Note: * Paired organs were weighed together.

Individual Absolute Organ Weights (g)

Group: G5 (Recovery Vehicle: 0 mg/kg B.wt./day) Sex: Female

Animal Number	Adrenals*	Kidneys*	Liver	Heart	Brain	Spleen
86	0.0903	1.9941	7.8879	0.9494	2.0051	0.7201
87	0.0895	2.2778	7.8832	0.9901	2.1039	0.7083
88	0.0942	1.7689	6.4766	0.8162	1.6472	0.8902
89	0.0552	1.4820	4.9682	0.7352	1.9938	0.9541
90	0.0733	1.8614	4.8731	0.9325	1.6527	0.7034

Animal Number	Ovaries*	Uterus with Cervix	Thymus	Thyroid Gland	Pituitary Gland
86	0.1186	0.6001	0.3979	0.0028	0.0133
87	0.1683	1.1728	0.3188	0.0027	0.0134
88	0.1537	0.9857	0.1979	0.0026	0.0136
89	0.1058	0.4358	0.2187	0.0029	0.0137
90	0.1483	0.9766	0.1809	0.0031	0.0139

Group: G6 (Recovery High Dose: 41.2 mg/kg B.wt./day) Sex: Female

Animal Number	Adrenals*	Kidneys*	Liver	Heart	Brain	Spleen
96	0.0892	1.7821	6.7231	0.9721	2.0085	0.8054
97	0.0908	1.9834	7.8423	0.8213	1.5989	0.7083
98	0.0869	1.8941	8.7322	0.7912	1.7648	0.6415
99	0.0984	1.7892	7.9862	0.8321	1.6074	0.6873
100	0.0748	1.9772	8.9469	0.8649	1.8939	0.7109

Animal Number	Ovaries*	Uterus and Cervix	Thymus	Thyroid Gland	Pituitary Gland
96	0.1109	0.6018	0.1731	0.0028	0.0140
97	0.1301	0.9676	0.1821	0.0027	0.0140
98	0.1092	0.6184	0.1764	0.0026	0.0135
99	0.1408	0.7633	0.1982	0.0025	0.0136
100	0.1084	0.6456	0.1849	0.0031	0.0137

Note: * Paired organs were weighed together.

Individual Relative Organ Weights (%)

Group: G1 (Vehicle: 0 mg/kg B.wt./day) Sex: Male

Animal Number	Fasted Body weight (g)	Adrenals*	Kidneys*	Liver	Heart	Brain	Spleen	Epididymis*
1	224.11	0.0238	0.8362	3.6643	0.4529	0.8132	0.3342	0.6632
2	229.01	0.0274	1.3514	5.8652	0.5436	0.8628	0.5361	0.6860
3	230.16	0.0215	1.0918	4.1784	0.5197	0.8674	0.2961	0.6137
4	234.51	0.0272	1.1362	4.6582	0.5078	0.7764	0.3645	0.5246
5	233.19	0.0270	0.9728	4.2687	0.4464	0.8150	0.2692	0.6154
6	236.61	0.0260	1.2496	4.5691	0.4916	0.8052	0.3693	0.5610
7	235.03	0.0241	1.3500	5.5817	0.6023	0.8391	0.5718	0.6380
8	236.61	0.0259	1.2300	4.0533	0.4824	0.8034	0.3414	0.5709
9	234.15	0.0243	1.1813	5.8267	0.4817	0.8069	0.3471	0.6794
10	234.59	0.0268	1.3465	6.1830	0.5233	0.8049	0.4259	0.5563

Animal Number	Testes*	Thymus	Thyroid Gland	Seminal Vesicle	Prostate + Seminal Vesicles with Coagulating Glands	Prostate	Pituitary Gland
1	1.3311	0.1937	0.0009	0.2043	0.4811	0.2856	0.0063
2	1.2599	0.1304	0.0012	0.2063	0.4709	0.2756	0.0058
3	1.2502	0.1276	0.0010	0.2043	0.4685	0.2794	0.0050
4	1.3828	0.2008	0.0009	0.2004	0.4599	0.2747	0.0056
5	1.2461	0.1808	0.0011	0.2026	0.4620	0.2764	0.0056
6	1.2943	0.1609	0.0011	0.1991	0.4552	0.2709	0.0051
7	1.3578	0.1601	0.0011	0.2053	0.4569	0.2736	0.0048
8	1.2143	0.0987	0.0010	0.2016	0.4540	0.2710	0.0051
9	1.2731	0.2059	0.0011	0.2051	0.4573	0.2736	0.0049
10	1.2605	0.2961	0.0011	0.1923	0.4574	0.2741	0.0050

Note: * Paired organs were weighed together.

Individual Relative Organ Weights (%)

Group: G2 (Low Dose: 10.3 mg/kg B.wt./day) Sex: Male

Animal Number	Fasted Body weight (g)	Adrenals*	Kidneys*	Liver	Heart	Brain	Spleen	Epididymis*
21	238.51	0.0236	1.2261	5.0070	0.5095	0.7691	0.2184	0.9277
22	235.40	0.0229	1.3246	4.6658	0.5397	0.8406	0.2430	0.4672
23	236.47	0.0268	1.3386	6.0738	0.5108	0.9296	0.2180	0.4794
24	234.11	0.0217	1.1607	4.3143	0.5070	0.7603	0.2077	0.5997
25	239.59	0.0281	1.5843	7.3619	0.5720	0.7045	0.3882	0.5781
26	235.01	0.0332	1.1984	4.8268	0.4716	0.7184	0.2419	0.6056
27	238.17	0.0372	1.2054	5.2523	0.5071	0.7493	0.2761	0.6333
28	235.59	0.0245	1.2542	5.8523	0.5836	0.7346	0.3197	0.5486
29	238.51	0.0253	1.1701	5.3962	0.5702	0.7833	0.2811	0.6885
30	239.74	0.0256	1.1251	7.3961	0.4286	0.6842	0.3280	0.4613

Animal Number	Testes*	Thymus	Thyroid Gland	Seminal Vesicle	Prostate + Seminal Vesicles with Coagulating Glands	Prostate	Pituitary Gland
21	1.4658	0.1510	0.0010	0.1750	0.4509	0.2680	0.0055
22	0.9256	0.1942	0.0010	0.1768	0.4566	0.2735	0.0056
23	1.3000	0.1373	0.0010	0.2039	0.4538	0.2712	0.0057
24	1.1891	0.1150	0.0010	0.2018	0.4587	0.2757	0.0060
25	1.0468	0.2616	0.0012	0.1947	0.4485	0.2709	0.0059
26	0.9605	0.2934	0.0013	0.2009	0.4572	0.2983	0.0058
27	1.5069	0.2148	0.0010	0.1922	0.4506	0.2910	0.0056
28	1.1277	0.1818	0.0010	0.1789	0.4560	0.2945	0.0056
29	1.1557	0.1805	0.0009	0.1788	0.4509	0.2825	0.0055
30	1.3076	0.2471	0.0009	0.1788	0.4476	0.2841	0.0052

Note: * Paired organs were weighed together.

Individual Relative Organ Weights (%)

Group: G3 (Mid Dose: 20.6 mg/kg B.wt./day) Sex: Male

Animal Number	Fasted Body weight (g)	Adrenals*	Kidneys*	Liver	Heart	Brain	Spleen	Epididymis*
41	234.91	0.0246	1.0782	5.1000	0.4929	0.7234	0.2585	0.5905
42	239.81	0.0220	1.0976	4.9135	0.4921	0.7415	0.3263	0.6193
43	242.11	0.0207	1.0964	4.7209	0.4303	0.7897	0.2396	0.6124
44	238.85	0.0253	1.0761	5.3545	0.4845	0.5533	0.3106	0.5438
45	239.81	0.0224	1.1603	5.7838	0.4674	0.6543	0.2711	0.6155
46	243.19	0.0255	1.3845	5.8753	0.4577	0.7505	0.2644	0.5581
47	237.41	0.0252	1.1827	5.8425	0.5666	0.7470	0.2767	0.6180
48	241.11	0.0237	1.1336	4.4607	0.4207	0.6802	0.2888	0.6031
49	236.47	0.0269	1.1453	5.0664	0.5381	0.7538	0.2758	0.6268
50	239.84	0.0237	1.0209	4.5254	0.4168	0.8473	0.3118	0.5199

Animal Number	Testes*	Thymus	Thyroid Gland	Seminal Vesicle	Prostate + Seminal Vesicles with Coagulating Glands	Prostate	Pituitary Gland
41	1.2816	0.1487	0.0012	0.1953	0.4568	0.2912	0.0056
42	1.1724	0.2010	0.0011	0.1970	0.4482	0.2900	0.0056
43	1.2463	0.0857	0.0010	0.1940	0.4435	0.2557	0.0058
44	1.2800	0.1900	0.0009	0.2018	0.4499	0.2638	0.0059
45	1.2061	0.1889	0.0011	0.1909	0.4487	0.2582	0.0054
46	1.2223	0.2303	0.0011	0.1935	0.4413	0.2598	0.0051
47	1.2470	0.2836	0.0013	0.1979	0.4523	0.2586	0.0053
48	1.3063	0.1905	0.0012	0.1950	0.4449	0.2662	0.0054
49	1.2224	0.2424	0.0011	0.1930	0.4537	0.2687	0.0052
50	1.1975	0.2621	0.0009	0.1981	0.4478	0.2669	0.0050

Note: * Paired organs were weighed together.

Individual Relative Organ Weights (%)

Group: G4 (High Dose: 41.2 mg/kg B.wt./day) Sex: Male

Animal Number	Fasted Body weight (g)	Adrenals*	Kidneys*	Liver	Heart	Brain	Spleen	Epididymis*
61	239.63	0.0260	1.5532	7.3765	0.5867	0.9003	0.6387	0.6312
62	238.83	0.0273	1.1105	6.5961	0.5779	0.8270	0.3656	0.5977
63	242.21	0.0254	1.1397	6.0941	0.5199	0.7724	0.3950	0.5997
64	243.54	0.0243	1.1047	5.1675	0.3957	0.6948	0.3650	0.5515
65	244.01	0.0259	1.4082	6.4063	0.5030	0.4778	0.3487	0.5786
66	246.61	0.0242	1.3809	7.0061	04689	0.7099	0.3543	0.5670
67	239.84	0.0315	1.0216	5.3113	0.4815	0.7556	0.2765	0.5309
68	238.57	0.0251	1.0763	5.7569	0.4765	0.7570	0.3500	0.6114
69	241.01	0.0252	1.1831	5.8749	0.4877	0.7780	0.3121	0.5407
70	243.31	0.0288	1.3469	5.4842	0.5659	0.7629	0.3232	0.5693

Animal Number	Testes*	Thymus	Thyroid Gland	Seminal Vesicle	Prostate + Seminal Vesicles with Coagulating Glands	Prostate	Pituitary Gland
61	1.5170	0.1734	0.0012	0.2024	0.4483	0.2887	0.0056
62	1.3085	0.2166	0.0011	0.1782	0.4497	0.2892	0.0055
63	1.3489	0.1891	0.0010	0.1951	0.4434	0.2515	0.0053
64	1.2666	0.2086	0.0009	0.1913	0.4403	0.2527	0.0058
65	1.2097	0.2601	0.0011	0.1935	0.4401	0.2541	0.0054
66	1.2937	0.1983	0.0011	0.1986	0.4356	0.2495	0.0055
67	1.2791	0.1486	0.0012	0.1775	0.4469	0.2540	0.0059
68	1.2793	0.2024	0.0011	0.1965	0.4495	0.2595	0.0055
69	1.2290	0.2114	0.0011	0.1960	0.4446	0.2619	0.0056
70	1.3489	0.3602	0.0010	0.1885	0.4406	0.2546	0.0053

Note: * Paired organs were weighed together.

Individual Relative Organ Weights (%)

Group: G5 (Recovery Vehicle: 0 mg/kg B.wt./day) Sex: Male

Animal Number	Fasted Body weight (g)	Adrenals*	Kidneys*	Liver	Heart	Brain	Spleen	Epididymis*
81	247.41	0.0291	1.4669	6.0294	0.5279	0.9665	0.3940	0.5444
82	251.01	0.0308	1.6324	6.3640	0.5556	0.8142	0.3237	0.5779
83	252.11	0.0269	1.1825	4.2953	0.5087	0.7732	0.3470	0.5646
84	254.06	0.0289	1.1793	4.6817	0.4753	0.8197	0.3553	0.6130
85	255.03	0.0307	1.2871	4.6697	0.5182	0.7581	0.2954	0.5288

Animal Number	Testes*	Thymus	Thyroid Gland	Seminal Vesicle	Prostate + Seminal Vesicles with Coagulating Glands	Prostate	Pituitary Gland
81	1.2620	0.1805	0.0011	0.1899	0.4341	0.2633	0.0049
82	1.2086	0.1926	0.0012	0.1907	0.4269	0.2546	0.0056
83	1.1831	0.2156	0.0010	0.1819	0.4254	0.2559	0.0051
84	1.2320	0.1852	0.0009	0.1802	0.4233	0.2484	0.0052
85	1.1561	0.1324	0.0010	0.1838	0.4202	0.2565	0.0055

Group: G6 (Recovery High Dose: 41.2 mg/kg B.wt./day) Sex: Male

Animal Number	Fasted Body weight (g)	Adrenals*	Kidneys*	Liver	Heart	Brain	Spleen	Epididymis*
91	252.19	0.0325	1.3194	4.6591	0.4495	0.7395	0.2400	0.5632
92	254.11	0.0246	1.4879	4.2915	0.5023	0.8031	0.3332	0.6048
93	251.01	0.0298	1.0210	4.8926	0.4588	0.7509	0.3031	0.5837
94	254.09	0.0281	1.3807	4.0812	0.5087	0.7597	0.2956	0.5617
95	250.91	0.0310	1.5465	4.3768	0.4717	0.7755	0.3550	0.5598

Animal Number	Testes*	Thymus	Thyroid Gland	Seminal Vesicle	Prostate + Seminal Vesicles with Coagulating Glands	Prostate	Pituitary Gland
91	1.2504	0.2303	0.0011	0.1688	0.4255	0.2740	0.0055
92	1.2038	0.1412	0.0010	0.1859	0.4232	0.2487	0.0050
93	1.1784	0.1902	0.0010	0.1921	0.4269	0.2594	0.0052
94	1.1219	0.1734	0.0009	0.1819	0.4223	0.2720	0.0055
95	1.2559	0.1749	0.0010	0.1709	0.4277	0.2547	0.0052

Note: * Paired organs were weighed together.

Individual Relative Organ Weights (%)

Group: G1 (Vehicle: 0 mg/kg B.wt./day) Sex: Female

Animal Number	Fasted Body weight (g)	Adrenals*	Kidneys*	Liver	Heart	Brain	Spleen
11	208.11	0.0374	0.7624	4.3145	0.3834	0.7285	0.3230
12	213.31	0.0398	0.8061	3.5234	0.4133	0.8111	0.3137
13	219.54	0.0376	0.7419	3.8483	0.3456	0.7365	0.3931
14	225.16	0.0321	0.7031	3.8795	0.3948	0.6914	0.2648
15	224.63	0.0370	0.6963	3.3675	0.3138	0.7882	0.3887
16	222.54	0.0336	0.7533	3.4040	0.3826	0.6787	0.2397
17	225.71	0.0329	0.7413	3.4984	0.2899	0.6058	0.3342
18	226.91	0.0248	0.7726	3.7726	0.3843	0.6341	0.3333
19	228.53	0.0415	0.9060	3.4297	0.4039	0.7665	02751
20	224.01	0.0376	0.8272	3.5158	0.4032	0.7166	0.3077

Animal Number	Ovaries*	Uterus with Cervix	Thymus	Thyroid Gland	Pituitary Gland
11	0.0677	0.3074	0.1396	0.0013	0.0067
12	0.0543	0.3771	0.1186	0.0012	0.0066
13	0.0668	0.2394	0.1938	0.0012	0.0058
14	0.0499	0.2444	0.2656	0.0011	0.0063
15	0.0782	0.3366	0.1362	0.0010	0.0061
16	0.0608	0.4349	0.1382	0.0011	0.0062
17	0.0798	0.3300	0.1675	0.0012	0.0062
18	0.0691	0.3778	0.1997	0.0011	0.0061
19	0.0633	0.1840	0.1956	0.0011	0.0060
20	0.0604	0.2559	0.1572	0.0010	0.0063

Note: * Paired organs were weighed together.

Individual Relative Organ Weights (%)

Group: G2 (Low Dose: 10.3 mg/kg B.wt./day) Sex: Female

Animal Number	Fasted Body weight (g)	Adrenals*	Kidneys*	Liver	Heart	Brain	Spleen
31	216.67	0.0412	0.8152	3.6155	0.4229	0.7202	0.3026
32	218.54	0.0339	0.8692	3.1652	0.4341	0.8630	0.2293
33	226.61	0.0322	0.7745	3.1251	0.3875	0.8367	0.2498
34	228.74	0.0385	0.6833	3.4421	0.3600	0.8701	0.1736
35	229.64	0.0265	0.7373	3.5955	0.3675	0.7765	0.1987
36	225.01	0.0351	0.7512	3.8056	0.3926	0.7209	0.1766
37	227.02	0.0325	0.7043	3.0679	0.3623	0.7098	0.1912
38	228.64	0.0317	0.6749	3.7934	0.3534	0.7712	0.2433
39	224.51	0.0335	0.7796	4.0134	0.4148	0.7809	0.2142
40	226.53	0.0306	0.7646	3.8861	0.3312	0.7739	0.1856

Animal Number	Ovaries*	Uterus with Cervix	Thymus	Thyroid Gland	Pituitary Gland
31	0.0780	0.2875	0.2949	0.0014	0.0063
32	0.0674	0.3453	0.1683	0.0012	0.0065
33	0.0676	0.2449	0.1426	0.0011	0.0062
34	0.0700	0.2536	0.2102	0.0011	0.0060
35	0.0590	0.2892	0.2409	0.0011	0.0056
36	0.0619	0.2980	0.2592	0.0011	0.0059
37	0.0532	0.2149	0.2058	0.0010	0.0062
38	0.0686	0.2492	0.2416	0.0010	0.0062
39	0.0549	0.3354	0.2923	0.0011	0.0058
40	0.0841	0.2486	0.2352	0.0012	0.0059

Note: * Paired organs were weighed together.

Individual Relative Organ Weights (%)

Group: G3 (Mid Dose: 20.6 mg/kg B.wt./day) Sex: Female

Animal Number	Fasted Body weight (g)	Adrenals*	Kidneys*	Liver	Heart	Brain	Spleen
51	225.41	0.0280	1.0114	4.6433	0.4646	0.7858	0.4701
52	226.64	0.0318	0.6884	3.3184	0.4013	0.7619	0.2271
53	227.54	0.0512	0.9006	4.1638	0.4173	0.7891	0.2850
54	229.87	0.0337	0.6860	3.1584	0.3553	0.8196	0.2640
55	225.40	0.0319	0.8794	3.3502	0.3539	0.5875	0.2940
56	226.64	0.0358	0.9312	3.3234	0.6023	0.8353	0.3243
57	228.39	0.0364	1.0162	3.3110	0.5346	0.7904	0.3424
58	225.19	0.0336	0.8793	3.9114	0.5565	0.8451	0.2456
59	223.54	0.0310	0.9627	3.5841	0.4767	0.8532	0.2699
60	228.51	0.0411	0.8445	3.4433	0.4091	0.7884	0.2825

Animal Number	Ovaries*	Uterus with Cervix	Thymus	Thyroid Gland	Pituitary Gland
51	0.0969	0.2099	0.3473	0.0015	0.0058
52	0.0563	0.2369	0.1798	0.0012	0.0059
53	0.0796	0.1212	0.2334	0.0014	0.0059
54	0.0656	0.3417	0.2149	0.0013	0.0060
55	0.0586	0.2312	0.2361	0.0012	0.0063
56	0.0583	0.2308	0.2441	0.0011	0.0062
57	0.0715	0.2461	0.1800	0.0013	0.0062
58	0.0796	0.2001	0.1768	0.0014	0.0064
59	0.0815	0.2234	0.1812	0.0012	0.0062
60	0.0447	0.2196	0.1194	0.0011	0.0061

Note: * Paired organs were weighed together.

Individual Relative Organ Weights (%)

Group: G4 (High Dose: 41.2 mg/kg B.wt./day) Sex: Female

Animal Number	Fasted Body weight (g)	Adrenals*	Kidneys*	Liver	Heart	Brain	Spleen
71	215.61	0.0334	0.8033	3.8377	0.3773	0.5834	0.3180
72	226.51	0.0332	1.0015	3.5276	0.3907	0.7114	0.4553
73	228.01	0.0321	0.7930	3.5335	0.4084	0.6982	0.2489
74	225.41	0.0279	1.1661	5.8834	0.3480	0.7684	0.2640
75	223.20	0.0257	0.7547	3.2771	0.3911	0.7500	0.4185
76	228.41	0.0232	0.9346	3.9009	0.4954	0.7452	0.2931
77	224.61	0.0334	0.8794	3.7986	0.3874	0.7045	0.3344
78	228.01	0.0295	0.9022	3.8192	0.4168	0.7257	0.2754
79	229.36	0.0266	0.7208	3.4859	0.3675	0.6884	0.2985
80	224.51	0.0326	0.8034	3.4022	0.4138	0.6780	0.2783

Animal Number	Ovaries*	Uterus with Cervix	Thymus	Thyroid Gland	Pituitary Gland
71	0.0738	0.1429	0.1270	0.0013	0.0064
72	0.0582	0.2935	0.1203	0.0012	0.0060
73	0.0835	0.2099	0.2644	0.0012	0.0062
74	0.0718	0.1977	0.2162	0.0012	0.0062
75	0.0645	0.3853	0.2104	0.0011	0.0062
76	0.0666	0.3087	0.1795	0.0009	0.0060
77	0.0699	0.2280	0.1884	0.0010	0.0062
78	0.0724	0.2547	0.2116	0.0013	0.0062
79	0.0668	0.1882	0.1796	0.0014	0.0062
80	0.0583	0.3121	0.2163	0.0013	0.0064

Note: * Paired organs were weighed together.

Individual Relative Organ Weights (%)

Group: G5 (Recovery Vehicle: 0 mg/kg B.wt./day) Sex: Female

Animal Number	Fasted Body weight (g)	Adrenals*	Kidneys*	Liver	Heart	Brain	Spleen
86	233.09	0.039	0.856	3.384	0.407	0.860	0.309
87	238.54	0.038	0.955	3.305	0.415	0.882	0.297
88	237.11	0.040	0.746	2.731	0.344	0.695	0.375
89	238.71	0.023	0.621	2.081	0.308	0.835	0.400
90	239.16	0.031	0.778	2.038	0.390	0.691	0.294

Animal Number	Ovaries*	Uterus with Cervix	Thymus	Thyroid Gland	Pituitary Gland
86	0.051	0.257	0.171	0.001	0.006
87	0.071	0.492	0.134	0.001	0.006
88	0.065	0.416	0.083	0.001	0.006
89	0.044	0.183	0.092	0.001	0.006
90	0.062	0.408	0.076	0.001	0.006

Group: G6 (Recovery High Dose: 41.2 mg/kg B.wt./day) Sex: Female

Animal Number	Fasted Body weight (g)	Adrenals*	Kidneys*	Liver	Heart	Brain	Spleen
96	240.51	0.037	0.741	2.795	0.404	0.835	0.335
97	236.19	0.038	0.840	3.320	0.348	0.677	0.300
98	235.19	0.037	0.805	3.713	0.336	0.750	0.273
99	240.01	0.041	0.745	3.327	0.347	0.670	0.286
100	235.09	0.032	0.841	3.806	0.368	0.806	0.302

Animal Number	Ovaries*	Uterus with Cervix	Thymus	Thyroid Gland	Pituitary Gland
96	0.046	0.250	0.072	0.001	0.006
97	0.055	0.410	0.077	0.001	0.006
98	0.046	0.263	0.075	0.001	0.006
99	0.059	0.318	0.083	0.001	0.006
100	0.046	0.275	0.079	0.001	0.006

Note: * Paired organs were weighed together.
